# Genome mining for natural product biosynthetic gene clusters in the Subsection V cyanobacteria

**DOI:** 10.1186/s12864-015-1855-z

**Published:** 2015-09-03

**Authors:** Melinda L. Micallef, Paul M. D’Agostino, Deepti Sharma, Rajesh Viswanathan, Michelle C. Moffitt

**Affiliations:** School of Science and Health, University of Western Sydney, Locked Bag 1797, Penrith, NSW 2751 Australia; School of Biotechnology and Biomolecular Sciences, University of New South Wales, Kensington, NSW 2052 Australia; Department of Chemistry, Case Western Reserve University, 2740 Millis Science Center, Adelbert Road, Cleveland, OH 44106 USA

## Abstract

**Background:**

Cyanobacteria are well known for the production of a range of secondary metabolites. Whilst recent genome sequencing projects has led to an increase in the number of publically available cyanobacterial genomes, the secondary metabolite potential of many of these organisms remains elusive. Our study focused on the 11 publically available Subsection V cyanobacterial genomes, together with the draft genomes of *Westiella intricata* UH strain HT-29-1 and *Hapalosiphon welwitschii* UH strain IC-52-3, for their genetic potential to produce secondary metabolites. The Subsection V cyanobacterial genomes analysed in this study are reported to produce a diverse range of natural products, including the hapalindole-family of compounds, microcystin, hapalosin, mycosporine-like amino acids and hydrocarbons.

**Results:**

A putative gene cluster for the cyclic depsipeptide hapalosin, known to reverse *P*-glycoprotein multiple drug resistance, was identified within three Subsection V cyanobacterial genomes, including the producing cyanobacterium *H. welwitschii* UH strain IC-52-3. A number of orphan NRPS/PKS gene clusters and ribosomally-synthesised and post translationally-modified peptide gene clusters (including cyanobactin, microviridin and bacteriocin gene clusters) were identified. Furthermore, gene clusters encoding the biosynthesis of mycosporine-like amino acids, scytonemin, hydrocarbons and terpenes were also identified and compared.

**Conclusions:**

Genome mining has revealed the diversity, abundance and complex nature of the secondary metabolite potential of the Subsection V cyanobacteria. This bioinformatic study has identified novel biosynthetic enzymes which have not been associated with gene clusters of known classes of natural products, suggesting that these cyanobacteria potentially produce structurally novel secondary metabolites.

**Electronic supplementary material:**

The online version of this article (doi:10.1186/s12864-015-1855-z) contains supplementary material, which is available to authorized users.

## Background

The phylum cyanobacteria consists of photosynthetic bacteria that are known to survive in a range of environments, and exhibit diverse morphology. The Subsection V cyanobacteria morphologically appear as true-branching filaments capable of forming heterocysts (specialised N_2_ fixing cells), akinetes (cyst-like resting cells) and hormogonia (differentiated motile trichomes), making them one of the most morphologically advanced groups of cyanobacteria [1].

Cyanobacteria are prolific producers of secondary metabolites [2–4]. In particular, the Subsection V cyanobacteria are well known for the production of the hapalindole-family of compounds, a group of structurally related indole alkaloids consisting of hapalindoles, welwitindolinones and fisherindoles which display a broad range of bioactivities [5, 6]. Other metabolites isolated from Subsection V cyanobacteria include the hepatotoxin microcystin [7–9], the cyclic peptide, hapalosin [10, 11], hydrocarbons [12], fischerellin A and B [13–15], the cyclic peptide westiellamide [16], the aromatic compounds ambigol A, B, C and 2,4-dichlorobenzoic acid [17, 18], the alkaloid tjipanazole D [18], the depsipeptide stigonemapeptin [19], the hexapeptide hapalocyclamide [20], the antimicrobial compound parsiguine [21], and the long chain polyunsaturated fatty acid γ-Linolenic acid [22].

The majority of cyanobacterial natural products are non-ribosomal peptides, polyketides or hybrid peptide-polyketide compounds [23–26]. Nonribosomal peptides are biosynthesised by nonribosomal peptide synthetases (NRPS), multifunctional enzyme complexes which assemble either proteinogenic or nonproteinogenic amino acids into the final peptide structure in an assembly line fashion [27]. Similarly, polyketides are biosynthesised by polyketide synthases (PKS), which assemble polyketides from acyl-CoA in a sequential manner [27]. Each NRPS or PKS module contains a series of domains; a minimum NRPS module consists of condensation (C) domain for catalysing peptide bond formation, an adenylation (A) domain for selection of the substrate, and a peptide carrier protein (PCP) domain. The amino acid selected and incorporated by the A domain can be predicted through the ten critical amino acids comprising the A domain binding pocket. Similarly to the NRPS modules, a minimum PKS module consists of a ketosynthase (KS) domain, an acyltransferase (AT) domain and an acyl carrier protein (ACP) domain, respectively. However, additional auxiliary domains, also known as tailoring domains, may also be present within each module, which creates structural diversity within the encoded natural product. Examples of NRPS auxiliary domains include epimerisation (E) domains, *N*-methyltransferase (NM) domains and heterocyclisation domains. PKS auxiliary domains include the *β*-ketoreductase (KR) domain, a dehydrogenase (DH) domain, enoyl-reductase (ER) domain, *O*-methyltransferase (MT) domains and *C*-methyltransferase (CM) domains [28, 29]. Furthermore, theioesterase (TE) domains and reduction domains are encoded in the final module for chain termination and release of the polypeptide or polyketide natural product. The genes encoding natural product biosynthesis are generally clustered together on the genome [30, 31], aiding in structural prediction of the metabolite based on bioinformatics.

Another major class of cyanobacterial natural products are ribosomally-synthesised and post translationally-modified peptides, known as RiPPs, which are biosynthesised by post-ribosomal peptide synthesis (PRPS) [32]. The RiPP is encoded within the core peptide/region of the structural gene, which is then post-translationally modified into the final RiPP [32–35]. Other cyanobacterial natural products include the UV-absorbing compounds (mycosporine-like amino acids (MAAs) and scytonemin), terpenes and hydrocarbons.

The discovery of the diverse range and sources of these natural products has been aided by recent genome sequencing efforts. Prior to 2013, the Subsection V cyanobacteria were significantly under-represented compared to the other cyanobacterial subsections in terms of the number of sequenced genomes [36]. Two recent cyanobacterial sequencing projects aimed at increasing the number of Subsection V genomes led to a significant increase in the number of publically available genomes [1, 37]. Currently, there are 11 Subsection V cyanobacterial genomes publically available (Table 1), specifically *Fischerella* sp. PCC 9339, *Fischerella* sp. PCC 9431, *Fischerella* sp. JSC-11, *Fischerella* sp. PCC 9605, *Fischerella muscicola* PCC 7414, *Fischerella muscicola* SAG 1427–1, *Fischerella thermalis* PCC 7521, *Mastigocladopsis repens* PCC 10914, *Chlorogloeopsis fritschii* PCC 6912, *Chlorogloeopsis* sp. PCC 9212 and *Mastigocoleus testarum* BC008.

**Table 1:** Comparison of sequenced Subsection V cyanobacterial genomes

Cyanobacterial genome	Genome size (Mb)	GC content (%)	CDS	Number of scaffolds	Environmental data	Reference
*Fischerella* sp. PCC 9339	8.01	40.12	6,720	13	N/A	[37]
*Fischerella* sp. PCC 9431	7.17	40.17	6,104	8	N/A	[37]
FS JSC-11	5.38	41.05	4,671	34	N/A	N/A
*Fischerella* sp. PCC 9605	8.08	42.60	7,060	12	Limestone, Jerucham, Har Rahama, Israel	[37]
*F. muscicola* PCC 7414	6.90	41.26	6,060	270	Thermal springs, N/A	[1]
*F. muscicola* SAG 1427-1	7.36	40.32	6,533	524	Soil, Allahabad, India	[1]
*F. thermalis* PCC 7521	5.44	41.02	4,660	141	Thermal springs, Yellowstone National Park, USA	[1]
*M. repens* PCC 10914	6.46	43.52	5,846	3	Soil, Tarragona, Spain	N/A
*M. testarum* BC008	15.87	48.21	13,458	975	N/A	N/A
*C. fritschii* PCC 6912	7.75	41.48	6,836	161	Soil, Allahabad, India	[1]
*Chlorogloeopsis* sp. PCC 9212	7.65	41.50	6,717	188	Thermal springs, Orense, Spain	[1]
*W. intricata* UH strain HT-29-1	7.05	40.13	6,016	220	Soil, Moen Island, Truk Atoll, Caroline Islands	This study
*H. welwitschii* UH strain IC-52-3	7.27	40.21	6,139	169	Freshwater, Australian Institute of Marine Sciences, Queensland	This study
Average:	7.72	41.66	6,678	209.08		

Previous genome mining has reported a preliminary overview of NRPS/PKS, PRPS and terpene genes from the five Subsection V cyanobacterial genomes sequenced by Shih et al. [37]. Additionally an in-depth analysis of NRPS/PKS gene cluster families, showing that the percentage of genome devoted to these gene clusters is higher in the Subsection V than other cyanobacterial subsections, has recently been reported by Calteau et al. [38].

Recently, the welwitindolinone (*wel*) gene cluster and the ambiguine (*amb*) gene cluster were identified from *H. welwitschii* UTEX B1830 (also known as *Fischerella* sp. PCC 9431) and *F. ambigua* UTEX 1903, respectively [39, 40]. Furthermore, the hapalindole (*hpi*), *wel* and *amb* gene clusters were also identified from the publically available *Fischerella* sp. PCC 9339, *Fischerella* sp. PCC 9431 and *F. muscicola* SAG 1427–1 genomes, and the recently sequenced *W. intricata* UH strain HT-29-1, *H. welwitschii* UH strain IC-52-3, *Fischerella* sp. ATCC 43239 and *F. ambigua* UTEX 1903 genomes [41]. The other biosynthetic gene clusters reported from the Subsection V cyanobacteria are a microcystin (*mcy*) gene cluster from *Fischerella* sp. PCC 9339 [37], MAA (*mys*) gene clusters from *C. fritschii* PCC 6912 [42], *Fischerella* sp. PCC 9339 [37] and *Fischerella* sp. PCC 9431 [38], and the fatty acyl ACP reductase (FAAR) and aldehyde deformylating oxygenase (ADO) pathway for hydrocarbon biosynthesis in all the Subsection V cyanobacterial genomes [12, 43].

It was the aim of this study to provide a complete overview of the diversity and distribution of secondary metabolite biosynthesis for all 11 publically available Subsection V cyanobacteria, in addition to two genomes sequenced by our research group, *W. intricata* UH strain HT-29-1 and *H. welwitschii* UH strain IC-52-3. We present both known and orphan gene clusters belonging to the NRPS/PKS, PRPS (specifically cyanobactin, microvirdin and bacteriocin gene clusters), UV-absorbing (MAA and scytonemin), hydrocarbon and terpene classes of natural products. This is the first study that includes identification and analysis of gene clusters from all natural product structural classes in the genomes of Subsection V cyanobacteria. Based on our analysis, we propose that the Subsection V cyanobacteria have the potential to produce a number of novel metabolites for which their structure and bioactivities have not yet been identified.

## Methods

### Genome data

The genomes of *W. intricata* UH strain HT-29-1 and *H. welwitschii* UH strain IC-52-3 were obtained from genomic DNA (gDNA) extracted, sequenced, assembled and annotated as described in Micallef et al. [41]. Briefly, cyanobacterial cultures of *W. intricata* UH strain HT-29-1and *H. welwitschii* UH strain IC-52-3 were obtained from the University of Hawaii cyanobacterial culture collection. gDNA was extracted as described in Morin et al. [44] and additional polysaccharides were removed as described in Wilson [45]. gDNA was sequenced, assembled and annotated by BGI (Beijing Genome Institute, China) using Illumina sequencing technology and Glimmer v3.0. The 11 publically available genomes were obtained from the Joint Genome Institute (JGI) Integrated Microbial Genomes (IMG) database and the National Centre for Biotechnology Information (NCBI).

### PCR and sequencing reactions

PCR was used to identify the A-KR didomain of the hapalosin gene cluster in *H. welwitschii* UH strain IC-52-3 and to close any gaps in the nucleotide sequence of orphan NRPS/PKS gene clusters from *W. intricata* UH strain HT-29-1 and *H. welwitschii* UH strain IC-52-3 genomes. Comparison of orphan NRPS/PKS gene clusters identified from these genomes were compared, and any potential gaps in the nucleotide sequences (N’s) were identified and targeted for sequencing. A 50 μL PCR reaction mixture contained 10 pmol of specific forward and reverse primer or 50 pmol of degenerate primers (Additional file 1) (Geneworks, Australia), 1 × PCR Buffer (KAPA Biosystems), 2.5 mM MgCl_2_, 1 pmol dNTPs (Fisher Biotec), 1 U of KapaTaq polymerase (KAPA Biosystems) and 50 ng of gDNA template. *Pfu* DNA polymerase (Sigma) was used in addition to KapaTaq at a ratio of 1:10 (v/v). Hotstart PCR was performed by first heating the samples to 95 °C. Thermal cycling was then performed with a 5 min denaturation cycle at 95 °C, followed by 30 cycles of 95 °C for 30 s, 55 °C for 30 s and 72 °C for 1 min per 1 kb. Thermal cycling was concluded with a final extension at 72 °C for 7 min. PCR products were visualized in 1 % agarose gels in TAE buffer and single bands were gel extracted and purified using the QIAquick spin gel extraction kit (QIAGEN). Single sequencing reactions were submitted to the Ramaciotti Centre for Genomics at the University of New South Wales.

### Bioinformatic software

All nucleotide sequences obtained from Illumina genome sequencing, annotated open reading frames, sequencing results from PCR products, and nucleotide sequences of *W. intricata* UH strain HT-29-1 and *H. welwitschii* UH strain IC-52-3 genomes were organised and visualised using Geneious Version 6.1.7 created by Biomatters (available from http://www.geneious.com/). The 11 publically available Subsection V cyanobacterial genomes were downloaded from either the NCBI repository or the DOE Joint Genome Institute (JGI) server and visualised using Geneious Version 6.1.7.

Alignments of nucleotide sequences of individual genes and gene clusters were performed using Geneious alignment with default settings. For protein alignments, Clustal Omega (Version 1.2.1) was used with default settings, except the order of the aligned sequences was changed from aligned to input [46].

### Genome mining of Subsection V cyanobacteria

Putative secondary metabolite gene clusters were originally identified using antiSMASH version 2.0 [47] with default settings. Annotations were refined manually using CDsearch and BLASTp (Basic Local Alignment Search Tool) to identify conserved domains [48, 49]. Each biosynthetic gene cluster was first categorised into the type of natural product encoded within the gene cluster. Comparative genomics identified homologous clusters in the genomes of the Subsection V cyanobacteria, and their gene organisation was compared. Homologous gene clusters in multiple genomes were also identified using the COG homology search tool in IMG JGI. NRPS/PKS gene clusters were analysed to determine domain structure and those reported have standard domain organization [27] as seen in the majority of other cyanobacterial NRPS/PKS gene clusters. Gene clusters located at the edge of contigs, encoding incomplete modules or containing gaps (N’s) in nucleotide sequence were considered to be incomplete, unless stated otherwise.

### Bioinformatic analysis of NRPS/PKS gene clusters

The domain organisation of NRPS and PKS gene clusters identified by antiSMASH was further analysed using the NRPS/PKS database [50]. The A domain substrate specificity for NRPS enzymes was predicted using NRPSpredictor2 [51]. Furthermore, NaPDoS was used to identify C and KS domains [52].

### Bioinformatic analysis of PRPS gene clusters

Cyanobactin, microvirdin and bacteriocin biosynthetic gene clusters were first identified using antiSMASH [47]. Then, BLASTp [49] was used to determine potential false positives (cyanobactin and microviridin) or additional gene clusters (bacteriocin). In order to identify putative bacteriocin HetP-type and DUF37-type precursors in the Subsection V cyanobacterial genomes, BLASTp was utilised using previously identified precursors (Ava_0198 was used to identify HetP-type precursors and Ava_4222 was used to identify DUF37-type precursors). Protein alignments of precursor peptides were performed using Clustal Omega [46]. N11P and NHLP-type precursors were identified using antiSMASH. The bacteriocin gene clusters were then manually divided into the seven groups previously described by Wang et al. [34]. The seven groups are separated based on the presence of specific genes and domains. There are two types of ABC transporter genes which encode C39 peptidases in bacteriocin gene clusters. The short type, which contain a C39 peptidase, an ABC transmembrane and an ATP-binding cassette domain, are found in groups III, IV and V. The long type, which contain an extra N-terminal nucleotide binding domain (CAP_ED), are found in group I, II and VI. The group V bacteriocin gene clusters also contain a gene encoding a bimodular protein containing only two CAP_ED domains. Groups IV and V also encode an additional ABC transporter with only an ABC transmembrane and an ATP-binding cassette domain. The group VII bacteriocin gene clusters contain a unique ABC transporter gene, which appears to be a fusion of a short and long type ABC transporter without the C39 peptidase domain in the long type. A HlyD protein, containing a type_1_hlyD domain, is found in every group, whilst a SurA protein, containing a rotamase domain, is found in groups I, II and VI. Bacteriocin gene clusters which could not be classified into these groups were labelled as unclassified.

### Bioinformatic analysis of mycosporine-like amino acid gene clusters

To identify the *mys* gene cluster encoding MAA biosynthesis in the Subsection V cyanobacterial genomes, PCC9339DRAFT_04157 (encoding 3DHQS) of the previously identified MAA gene cluster in *Fischerella* sp. PCC 9339 [37] was used to performed a BLASTp search in the JGI/IMG database. Any positive matches were then manually searched for the essential *O*-MT, ATP-Grasp and NRPS-like/ATP-ligase enzymes downstream of the 3DHQS gene.

### Bioinformatic analysis of hydrocarbon, terpenes and alkaloid gene clusters

The hydrocarbon biosynthetic gene cluster was identified using known genes (HT291_00281 and HT291_02280) encoding the FAAR/ADO pathway in the Subsection V cyanobacteria [12]. The sulfotransferase domain, characteristic of the olefin synthase (OLS) pathway, was used in BLASTp analysis. Terpene biosynthetic gene clusters were identified using antiSMASH, and grouped based on the presence of common genes, and compared to known terpene and squalene biosynthetic gene clusters.

### Nucleotide sequence accession numbers

The draft genomes of *W. intricata* UH strain HT-29-1 and *H. welwitschii* UH strain IC-52-3 are available from the US DOE JGI IMG server under the Taxon ID 2529292565 https://img.jgi.doe.gov/cgi-bin/mer/main.cgi?section=TaxonDetail&page=taxonDetail&taxon_oid=2529292565 and 2529292566 https://img.jgi.doe.gov/cgi-bin/mer/main.cgi?section=TaxonDetail&page=taxonDetail&taxon_oid=2529292566, respectively.

## Results and discussion

### Genome characteristics of *Westiella intricata* UH strain HT-29-1 and *Hapalosiphon welwitschii* UH strain IC-52-3

*W. intricata* UH strain HT-29-1 and *H. welwitschii* UH strain IC-52-3 were chosen as candidates for analysis of secondary metabolite gene clusters, based on their potential to produce natural products of interest, specifically the hapalindole-type of natural products and hapalosin. Therefore, we conducted draft genome sequence analysis of *W. intricata* UH strain HT-29-1 and *H. welwitschii* UH strain IC-52-3 not only to improve the genomic coverage of the Subsection V cyanobacteria, but also to identify and compare the secondary metabolite potential of these cyanobacteria. The genome sequence of *W. intricata* UH strain HT-29-1 was assembled into 220 scaffolds encoding 6,086 coding sequences (CDS), whilst that of *H. welwitschii* UH strain IC-52-3 was assembled into 169 scaffolds encoding 6,209 CDS. The genome size of *W. intricata* UH strain HT-29-1 was determined to be 7.05 Mb, whilst that of *H. welwitschii* UH strain IC-52-3 was slightly larger at 7.27 Mb. The GC content for *W. intricata* UH strain HT-29-1 was 40.13 %, whilst that of *H. welwitschii* UH strain IC-52-3 was highly similar with a GC content of 40.21 %. The sizes of the Subsection V cyanobacterial genomes sequenced range from 5.38 to 15.87 Mb, with an average of 7.9 Mb. The genome size of *W. intricata* UH strain HT-29-1 and *H. welwitschii* UH strain IC-52-3 is slightly below this average, but well within the range of the Subsection V cyanobacterial genomes. Similarly, the number of CDS from the Subsection V cyanobacteria range from 4,671 to 11,113. Both *W. intricata* UH strain HT-29-1 and *H. welwitschii* UH strain IC-52-3 encode just below the average number of CDS, but within the range of the currently available genome sequences.

A survey of 102 housekeeping genes, previously identified as nearly universal in bacteria [53], were all identified in both *W. intricata* UH strain HT-29-1 and *H. welwitschii* UH strain IC-52-3 draft genomes (Additional file 2). The identification of all 102 housekeeping genes within both cyanobacterial genomes suggests a near complete genome. The largest percentage of functionally categorised genes in both *W. intricata* UH strain HT-29-1 and *H. welwitschii* UH strain IC-52-3 (based on cluster of orthologous groups categories, COGs) appears to be those involved in cell wall/membrane/envelope biogenesis (7 %), amino acid transport and metabolism (6.7 %) and signal transduction mechanisms (6 %) (Additional file 2).

### Secondary metabolite biosynthetic gene clusters

A diverse range of secondary metabolite biosynthetic gene clusters were identified from the genomes of the Subsection V cyanobacteria. These range from NRPS/PKS biosynthetic gene clusters, including a putative hapalosin (*hap*) gene cluster and RiPPs, including microviridin, cyanobactin and bacteriocin gene clusters. Additional gene clusters identified from the Subsection V cyanobacteria include the UV-absorbing compounds (MAA and scytonemin), hydrocarbons, terpenes, and the alkaloids (including the hapalindoles and welwitindolinones). Each Subsection V cyanobacterium dedicates between 0.87–3.5 % of the genome to secondary metabolite biosynthesis (Table 2) (however, this may be an underestimation as orphan NRPS/PKS gene clusters which appeared to be incomplete were not included in this calculation). *W. intricata* UH strain HT-29-1 and *Fischerella* sp. PCC 9339 dedicate the highest percentage of their genome to secondary metabolite biosynthesis (~3.5 %), followed closely by *H. welwitschii* UH strain IC-52-3 and *Fischerella* sp. PCC 9431 (~3.3 %). However, *M. testarum* BC008 dedicate the least percentage of its genome to secondary metabolite biosynthesis (~0.87 %) (Table 2).

**Table 2:** Size and percentage of secondary metabolite biosynthetic gene clusters identified from the Subsection V cyanobacteria

Subsection V cyanobacterium	Type of cyanobacterial gene cluster and size (kb)^Ω^											Percentage of genome
	NRPS/PKS/Hybrid			RiPPs			UV-absorbing compounds				Alkaloids	
	Hapalosin	Microcystin	Orphan NRPS PKS (number of orphan clusters)	Microviridin	Cyanobactin	Bacteriocin	MAA	Scytonemin	Hydrocarbon	Terpene	Hapalindoles/ welwitindolinones	
*W. intricata* UH strain HT-29-1	25.6	-	42.2 (1)	-	11.7	82.5	7.4	-	2.3	19	59.3	3.5
*H. welwitschii* UH strain IC-52-3	25.6	-	56.6 (2)	-	-	71.2	7.4	-	2.3	19	55.8	3.3
*Fischerella* sp. PCC 9431	25.8	-	57.5 (2)	-	-	66.5	7.4	-	2.3	19	57.1	3.3
*Fischerella* sp. PCC 9339	-	67.5	75.5 (4)	8.8	-	65.7	6.6	-	2.2	13.6	44.9	3.5
*F. muscicola* SAG 1427-1	-	-		-	-	57.6	6.5	-	2.2	17.6	25.1	1.5
*C. fritschii* PCC 6912	-	-	55.4 (2)	-	-	56.9	8.1	-	1.9	7.9	-	1.7
*Chlorogloeopsis* sp. PCC 9212	-	-	55.4 (2)	-	-	56.9	8.1	-	1.9	7.9	-	1.7
*M. testarum* BC008	-	-	43.1 (2)	-	-	74.1	8.3	-	2.0	9.8	-	0.87
*M. repens* PCC 10914	-	-	63.3 (4)	-	-	40.8	-	32.9	1.9	6.6	-	2.3
*Fischerella* sp. PCC 9605	-	-	38.3 (3)	-	-	103.2	-	-	2.0	13.9	-	2.0
*F. muscicola* PCC 7414	-	-	-	-	-	52.7	-	-	2.2	13.6	-	1.0
*Fischerella* sp. JSC-11	-	-	-	-	-	56.3	-	-	2.2	13.5	-	1.3
*F. thermalis* PCC 7521	-	-	-	-	-	45.7	-	-	2.2	13.5	-	1.1

^Ω^Total size of all genes clusters within each category

### NRPS/PKS biosynthetic gene clusters with a known product

#### Hapalosin gene cluster

**Fig. 1:**
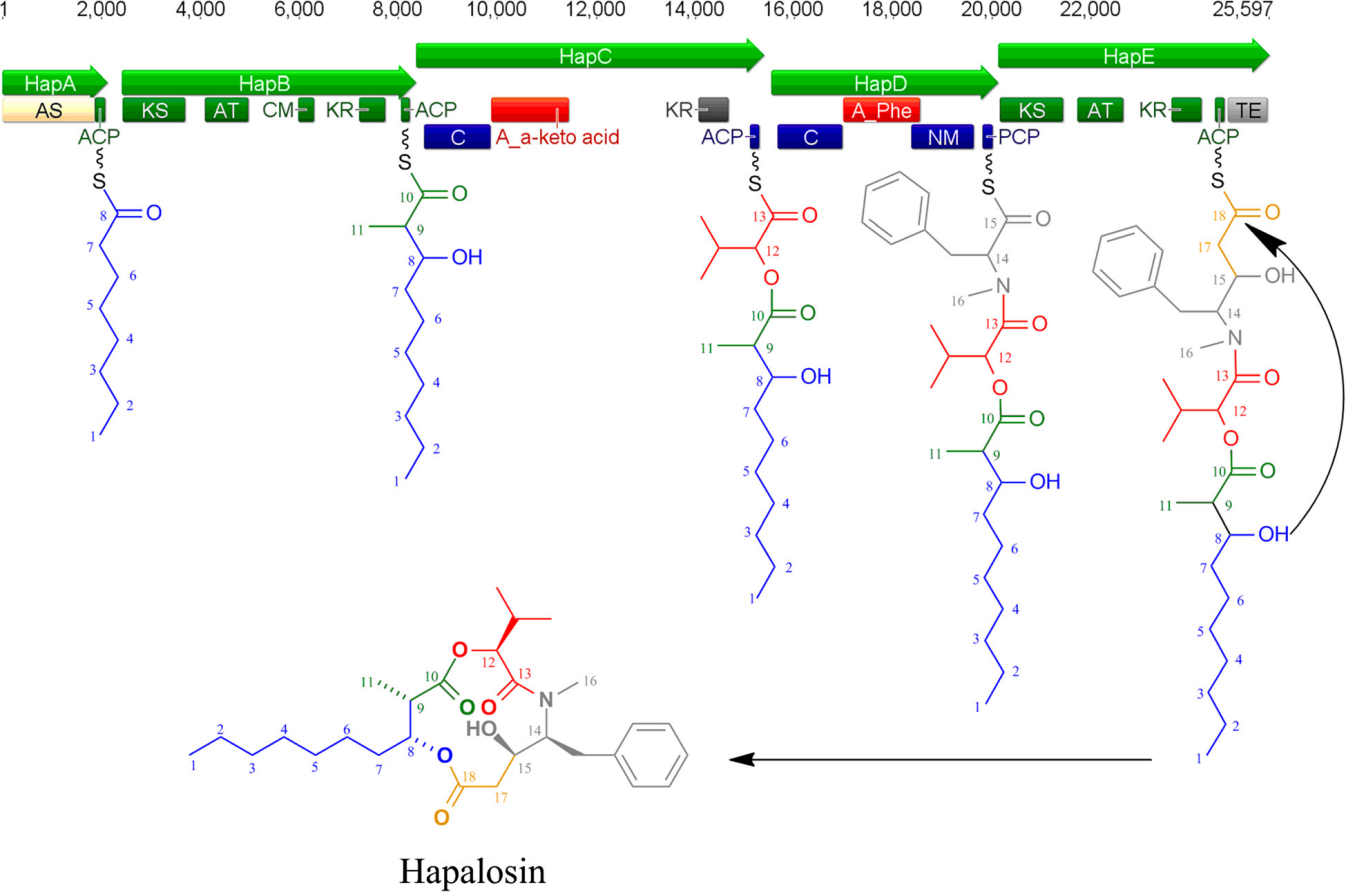
Predicted *hap* biosynthetic gene cluster, including domain organisation and biosynthetic pathway of hapalosin. The *hap* gene cluster is proposed to encode an initiation module with an AS domain for selection of octanoic acid as the starter unit, two NRPS modules and two PKS modules

A candidate gene cluster for the biosynthesis of hapalosin was identified in three Subsection V cyanobacterial genomes, specifically *H. welwitschii* UH strain IC-52-3, *W. intricata* UH strain HT-29-1 and *Fischerella* sp. PCC 9431, although hapalosin has only been reported from *H. welwitschii* UH strain IC-52-3 [10]. Prior to genome sequencing, a degenerate primer approach (Additional file 1) targeting conserved regions of A-KR didomains in *H. welwitschii* UH strain IC-52-3 [54] was utilised as bait to identify the *hap* gene cluster within the *H. welwitschii* UH strain IC-52-3 genome, and then within the remaining Subsection V cyanobacterial genomes. Thus, the complete putative *hap* biosynthetic gene cluster was identified within *H. welwitschii* UH strain IC-52-3, *W. intricata* UH strain HT-29-1 and *Fischerella* sp. PCC 9431 genomes on a single scaffold. The gene cluster is proposed to be ~25.6 kb in length, comprising five genes, *hapA-E* (Additional file 3). Overall, there is greater than 99.2 % amino acid sequence similarity between the three gene clusters, suggesting the proteins are homologous and likely perform the same function in all strains. The overall genetic architecture and domain organisation is consistent with the proposed biosynthesis of hapalosin (Fig. 1).

*hapA* encodes a protein with the domain organisation AS-ACP, which displays high similarity to HctA and JamA from the hectochlorin (*hct*) and jamaicamide (*jam*) biosynthetic gene clusters, respectively [55, 56]. HctA is proposed to activate free hexanoic acid for initiation of hectochlorin biosynthesis, and JamA has been shown to activate 5-hexanoic acid [55, 56]. Based on the structure of hapalosin, HapA is likely to activate free octanoic acid forming C1-C8 (Fig. 1).

*hapB,* encoding a PKS module with the domain organisation KS-AT-CM-KR-ACP, likely incorporates malonyl-CoA to produce C9-C10 of hapalosin, followed by methylation of C9 to produce C11 *via* the CM domain, and reduction of the carbonyl group of C8 to a hydroxyl group by the KR domain. Next, *hapC* displays high sequence similarity to HctE and HctF of the *hct* gene cluster, NpnA of the nostophycin gene cluster and CrpD of the cryptophycin gene cluster [55, 57, 58]. HctE, HctF, NpnA and CrpD all contain a rare A-KR didomain within a NRPS module. These KR domains are found embedded between the core motifs A8 and A9 of the A domain [59], and within the A domain binding pocket, the invariable Asp235 of amino acid incorporating A domains has been replaced by Val235. This replacement was identified in HapC in all three gene clusters (Additional file 3). The A domain of HctE and HctF has been shown to incorporate 2-oxoisovaleric acid through ATP-PPi exchange assays [55]. The A domain binding pocket of HapC is identical to HctE and HctF, and along with the structure of hapalosin, suggests an identical substrate is incorporated (Additional file 3). Therefore, *hapC,* encoding an NRPS module with the domain organisation C-A-KR-PCP is proposed to incorporate 2-oxoisovaleric acid, which is then reduced to 2-hydroxyisovaleric acid, producing C12 and C13 of hapalosin.

The NRPS HapD has the domain organisation C-A-NM-PCP. Analysis of the A domain binding pocket suggests phenylalanine is selected and activated by HapD (Additional file 3), producing C14 and C15 of hapalosin. Subsequent *N*-methylation of phenylalanine by the NM domain leads to the biosynthesis of C16 methyl substituent.

*hapE* encodes a single PKS module with the domain organisation KS-AT-KR-ACP-TE, which is identical to the PKS organisation of JamP, which displays the highest amino acid sequence similarity with HapE. Analysis of the AT domain of HapE suggests malonyl-CoA is incorporated, forming C17 and C18 of hapalosin. Subsequent reduction of the carbonyl group of C15 produces the hydroxyl group. The TE domain is proposed to break the thioester bond connecting the hapalosin chain to the ACP domain, enabling the oxygen of C8 to attack C18, forming the cyclised final structure. Together, the domain architecture and prediction of substrates is consistent with the structure of hapalosin.

The complete NRPS/PKS biosynthetic gene cluster for hapalosin biosynthesis is the first report of a biosynthetic gene cluster for this natural product. The *hap* gene cluster demonstrates similar domain architecture to the *hct* gene cluster from *L. majuscula* [55]. The first three modules are almost identical, with the exclusion of a halogenase domain from *hctB,* and a CM domain from *hctD.* The identification of the *hap* biosynthetic gene cluster in three of the Subsection V cyanobacteria was surprising, as only *H. welwitschii* UH strain IC-52-3 was reported to produce hapalosin. The conservation of the *hap* gene cluster across three genera suggests there is an unknown evolutionary significance/benefit to the organism, similar to the conservation of the saxitoxin gene cluster within cyanobacteria [60].

#### Microcystin gene cluster

The only known NRPS/PKS gene cluster previously identified from the Subsection V cyanobacterial genomes is the *mcy* gene cluster from *Fischerella* sp. PCC 9339. According to Shih et al. [37], the 67.5 kb gene cluster encodes *mcyA-I,* and there is an additional PKS gene with partial sequence similarity to *npnA* from the nostophycin gene cluster from *Nostoc* sp. 152 [57], and *mcyB* is located on the border of two contigs. The remaining Subsection V cyanobacterial genomes were screened for a *mcy* and other toxin biosynthetic gene clusters, however, no other toxin biosynthetic gene clusters were identified.

### Orphan NRPS/PKS biosynthetic gene clusters

A total of 103 orphan NRPS/PKS/Hybrid gene clusters were identified from the Subsection V cyanobacterial genomes which produce unknown products. Analysis of the NRPS and PKS domain composition enabled these gene clusters to be divided into those that likely encode NRPS/PKS natural products and those which appear to be incomplete gene clusters. A total of 17 gene clusters were identified from nine of the Subsection V cyanobacterial genomes which are potentially complete and have been given arbitrary cluster identification numbers (Additional file 4). Four of these gene clusters were identified in more than one cyanobacterial strain. These gene clusters were then compared with the remaining identified gene clusters. Two incomplete gene clusters share homology with a complete gene cluster from another genome (Additional file 5). The remaining gene clusters were categorised as incomplete, either encoding incomplete modules, gaps (N’s) in nucleotide sequence, or located on contig borders (Additional file 5). These incomplete gene clusters will not be discussed.

Three NRPS gene clusters (Cluster 1–3) were identified from four Subsection V cyanobacterial genomes which lack a C domain in the initiation module. There are four additional gene clusters (Clusters 14–17) which also contain genes encoding NRPS modules, however, the first module also encodes a C domain. It is unclear if these gene clusters are incomplete (missing an initiation module) or if these gene clusters encode an NRPS natural product.

The remaining 10 gene clusters which likely encode a NRPS/PKS natural product all encode an initiation module containing an AS domain which likely selects for a fatty acid molecule as the starter unit for biosynthesis, such as those observed in the *jam*, *hct* and *hap* gene clusters. AS domains have only been identified as initiation modules in these three NRPS/PKS gene clusters within cyanobacteria. This high proportion of AS domains within initiation modules within the Subsection V cyanobacteria is therefore unique.

Overall, prediction of the substrate selected and incorporated by the A domain was difficult due to the low similarity of the A domain binding pocket to known amino acids in the NRPSpredictor2 database. The nearest neighbour for each A domain binding pocket is reported, and the percentage identity to nearest signature for each A domain binding pocket is provided in Additional file 4. However, due to this low similarity for some A domains, and the unknown length of the proposed fatty acid substrate incorporated by the AS domain, the predicted structure of the encoded natural product cannot be proposed.

The majority of the orphan NRPS/PKS gene clusters analysed in this study included genes encoding tailoring enzymes which are not typically identified within NRPS/PKS gene clusters. Four NRPS/PKS gene clusters identified from the Subsection V cyanobacterial genomes (Cluster 6–9) contained genes encoding for dioxygenases and/or glycosyltransferases. The dioxygenase genes identified within these gene clusters display similarity to α-ketoglutarate-dependent, taurine dioxygenases. These enzymes catalyse the hydroxylation of taurine to produce sulphite and aminoacetaldehyde [61]. The dioxygenase genes precede NRPS genes within each orphan NRPS/PKS gene cluster, and are possibly involved in hydroxylation of the selected amino acid. Only Cluster 8 encodes a glycosyltransferase gene within an orphan NRPS/PKS gene cluster, which is likely to be involved in the addition of a sugar moiety on the encoded natural product. Glycosylated natural products display a wide range of bioactivities including insecticidal [62] and antitumor [63] activity, amongst others [64].

There are two gene clusters identified from the Subsection V cyanobacterial genomes (Cluster 10 and 11) which encode fatty acid desaturase genes within an NRPS/PKS gene cluster. These genes are located downstream from the AS domain, suggesting the fatty acid incorporated by the AS domain may be desaturated in the final natural product. In Cluster 3, a gene encoding a fatty acid desaturase is located upstream from the NRPS gene cluster, however, as this gene cluster does not encode an AS domain, the function of this enzyme remains unknown. The remaining orphan NRPS/PKS gene clusters contain genes encoding hypothetical proteins (Clusters 11–13), aspartate racemase (Cluster 9) and asparagine synthase and racemase (Cluster 17). However, the effect of these proteins on the encoded natural product cannot be predicted at this time.

Genome mining orphan NRPS/PKS gene clusters from the Subsection V cyanobacteria has uncovered novel biosynthetic gene clusters with unique genes, however, screening for the products themselves or characterisation of the enzymatic pathways is necessary to determine if the gene clusters are functional, or remnants of evolution. If functional, these gene clusters, which encode new enzymes within NRPS/PKS gene clusters, may perform new biosynthetic reactions, which may potentially produce natural products with enhanced or new bioactivities. The ability to successfully express cyanobacterial gene clusters in *Streptomyces* and *E. coli* hosts [65–71] means these gene clusters could potentially be characterised, and the bioactivity (if any) could eventually be determined.

There is also the potential to identify new products from previously identified biosynthetic gene clusters, such as the *hap* or *mcy* gene clusters. Characterisation of the barbamide gene cluster from *M. producens* in *Streptomyces venezuelae* [65] led to the production of the previously unidentified 4-*O*-demethylbarbamide, which was found to be more potent as a molluscicidal agent than barbamide. Therefore, characterisation of these biosynthetic gene clusters has the potential to lead to the identification of natural product analogues with enhanced bioactivities.

### PRPS biosynthetic gene clusters

The RiPPs are a growing class of natural products which are being increasingly recognised within cyanobacteria . Three distinct classes of RiPPs were identified from the Subsection V cyanobacteria in this study, specifically cyanobactins, microviridins and bacteriocins.

#### Cyanobactin biosynthetic gene cluster

**Fig. 2:**
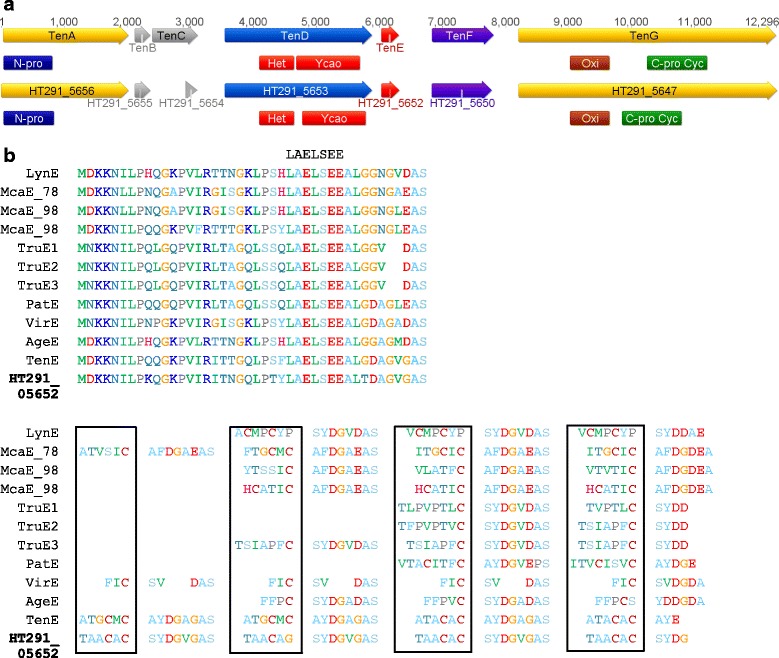
Cyanobactin gene cluster from *W. intricata* UH strain HT-29-1 and precursor analysis. **a** The cyanobactin gene cluster from *W. intricata* UH strain HT-29-1 was aligned with the *ten* gene cluster *Nostoc spongiaeforme* var. *tenue*. **b** Alignments of cyanobactin precursor peptides encoding multiple cyanobactins with precursor peptide sequence from *W. intricata* UH strain HT-29-1. The highly conserved LAELSEE motif is indicated above the precursor peptide sequences. The core peptide sequences are indicated in the box. Copies of each core peptide sequence range from one to four copies. *W. intricata* UH strain HT-29-1 encodes two different cyanobactins, with one (TAACAG) and three (TAACAC) copies of each core peptide. LynE from aestuaramide gene cluster (*Lyngbya* sp. PCC 8106) [94, 95], McaE from microcyclamide gene cluster (*Microcystis aeruginosa* PCC 7806, PCC 9809 and PCC 9806 respectively) [72, 96], TruE1,2,3 from trunkamide gene cluster (*Prochloron*) [94], PatE from patellamide gene cluster (*Prochloron*) [67], VirE from viridisamide gene cluster (*Oscillatoria nigro-viridis* PCC 7112) [72], AgeE from aeruginosamide gene cluster (*Microcystis aeruginosa* PCC 9432) [72], TenE from tenuecyclamide gene cluster (*Nostoc spongiaeforme* var. *tenue*) [68]; HT291_05652 from putative cyanobactin biosynthetic gene cluster (*Westiella intricata* UH strain HT-29-1)

Cyanobactins, N-C terminally cyclised peptides, are often prenylated or contain heterocyclised cysteine, serine or threonine residues, however linear cyanobactins have recently been discovered [72, 73]. A single cyanobactin biosynthetic gene cluster was identified from the Subsection V cyanobacteria, specifically from the genome of *W. intricata* UH strain HT-29-1 (Fig. 2a). The putative 11.7 kb cyanobactin biosynthetic gene cluster from *W. intricata* UH strain HT-29-1 encodes seven genes, which share homology to the tenuecyclamide (*ten*) gene cluster from *Nostoc spongiaeforme* var. *tenue* [68]. The cyanobactin gene cluster from *W. intricata* UH strain HT-29-1 appears to be intact, although a truncated homologue of the non-essential *tenC* gene is present. Within the putative *W. intricata* UH strain HT-29-1 cyanobactin gene cluster, HT291_05652 demonstrates homology with TenE, the precursor peptide for tenuecyclamide biosynthesis. Protein alignments of HT291_05652 with other known cyanobactin precursors revealed the presence of the highly conserved LAELSEE motif in the leader sequence, and the presence of four core peptide sequences. Comparison of the four core peptide sequences revealed one (TAACAG) and three (TAACAC) copies of the core peptide sequences, suggesting two different cyanobactins are biosynthesised by *W. intricata* UH strain HT-29-1 (Fig. 2b).

The remaining genes in the *W. intricata* UH strain HT-29-1 cyanobactin gene cluster are required for modifications of the precursor peptide. HT291_05656 and HT291_05647, which are homologous to TenA and TenG, respectively, are predicted to cleave the N- and C-terminus of the precursor peptide. Furthermore, PatG (homologous to TenG) has been shown to be responsible for macrocyclisation to form the final cyclic peptide [74], suggesting the final peptide from *W. intricata* UH strain HT-29-1 may also be cyclised.

Tenuecyclamides contain heterocyclised amino acids [75], which are proposed to be catalysed by TenD and the oxidase domain of TenG [68]. HT291_05653 is homologous to TenD, whilst HT291_05647 is homologous to TenG and contains an oxidase domain, suggesting the tyrosine and cysteine residues in the core peptide sequences may be heterocyclised in the final product. Furthermore, a homologous protein to the putative prenyltransferase TenF was identified in the cyanobactin gene cluster from *W. intricata* UH strain HT-29-1 (HT291_05650). The presence of this protein encoded in the cyanobactin gene cluster from *W. intricata* UH strain HT-29-1 suggests tyrosine may also be prenylated in the final cyanobactin product. The single precursor gene from *W. intricata* UH strain HT-29-1 encoding two different cyanobactins both appear to be novel, even though over 100 cyanobactin variants are known [76]. Recent genome mining of 126 cyanobacterial genomes revealed approximately 24 % of cyanobacterial strains encoded a cyanobactin biosynthetic gene cluster [72], however, many of these gene clusters are predicted to be non-functional [72], although active versions have been identified within closely related strains [77]. The identification of the cyanobactin gene cluster within *W. intricata* UH strain HT-29-1 encoding two novel cyanobactins contributes to the structural diversity of cyanobactins within cyanobacteria, whilst highlighting the diversity of natural products encoded within the Subsection V cyanobacteria.

#### Microviridin biosynthetic gene cluster

**Fig. 3:**
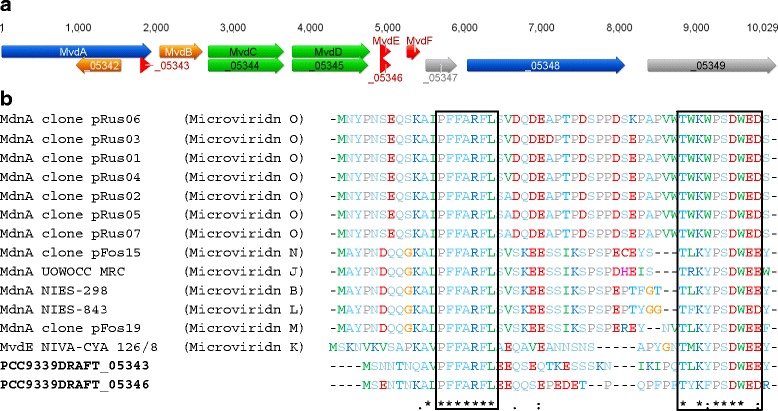
Microviridin gene cluster from *Fischerella* sp. PCC 9339 and precursor analysis. **a** The microviridin gene cluster from *Fischerella* sp. PCC 9339 was aligned with the *mvd* gene cluster from *Planktothrix agardhii* NIVA-CYA 126/8. **b** Alignment of selected known microviridin precursor peptide sequences with new putative microviridin precursor peptide sequences from *Fischerella* sp. PCC 9339. The PFFARFL region of the leader peptide and the conserved core peptide region are identified in the boxes. MdnA prepeptide sequences from uncultured *Microcystis* sp. clones pRus01-07 (GenBank: KF742389-KF742395), uncultured *Microcystis* sp. clones pFos15 and 19 (GenBank: KF742386 and KF742388), *Microcystis* UOWOCC MRC (GenBank: CAQ16121), *Microcystis* aeruginosa NIES-298 (GenBank: CAQ16116), *Microcystis* aeruginosa NIES-843 (GenBank: BAG02233) and MvdE from *Planktothrix agardhii* NIVA-CYA 126/8 (GenBank: ACC54551-ACC54552)

The microviridins are a family of *N*-acetylated tricyclic depsipeptides which contain a rare cage-like architecture [78, 79]. The peptide sequence is encoded within the ribosomal precursor peptide, which undergoes post-translational modifications including macrocyclisation by ATP grasp-type ligases [80, 81]. Genome mining of the Subsection V cyanobacterial genomes revealed only *Fischerella* sp. PCC 9339 encoded a microviridin (*mvd*) gene cluster (Fig. 3a). The novel eight gene biosynthetic cluster displays high sequence similarity with the *mvd* gene cluster from *Planktothrix agardhii* NIVA-CYA 126/8 [80]. The 8.8 kb *mvd* gene cluster from *Fischerella* sp. PCC 9339 encodes two precursor peptides (PCC9339DRAFT_05343 and PCC9339DRAFT_05346). Protein alignments with other known *mvd* precursor peptide sequences revealed the *Fischerella* sp. PCC 9339 peptide sequences were novel, therefore, the encoded microviridin variant could not be proposed (Fig. 3b). The putative *mvd* gene cluster from *Fischerella* sp. PCC 9339 encodes two cyclisation proteins for amide and ester bond formation (homologous to MvdC and MvdD), as well as an ABC transporter (homologous to MvdA). Furthermore, the putative *mvd* gene cluster from *Fischerella* sp. PCC 9339 encodes a protein similar to GCN5-related *N*-acetyltransferase proteins. The microviridin gene cluster from *M. aeruginosa* NIES 298 also encodes a GCN5-related *N*-acelyltransferase, suggesting the microviridin variant produced by *Fischerella* sp. PCC 9339 may be acetylated [82]. Finally, the putative *mvd* gene cluster from *Fischerella* sp. PCC 9339 also encodes a protein belonging to the GUN4 superfamily and a hypothetical protein. A putative *mvd* biosynthetic gene cluster from *N. spumigena* CCY 9414 also encodes a protein belonging to the GUN4 superfamily [80], however, the function and/or involvement of these proteins in microviridin biosynthesis remains unknown.

#### Bacteriocin biosynthetic gene clusters

Bacteriocins are another major class of RiPPs identified from cyanobacteria. Bacteriocins are encoded within short ribosomally produced precursor peptides, which contains both the core peptide and leader peptide [83]. The leader peptide is then cleaved by a peptidase domain during maturation [84]. The core peptide sequence can then undergo further post-translational modification, including macrocyclisation, dehydration, heterocyclization, as well as lanthionine formation, to produce the final RiPP [85, 86].

**Fig. 4:**
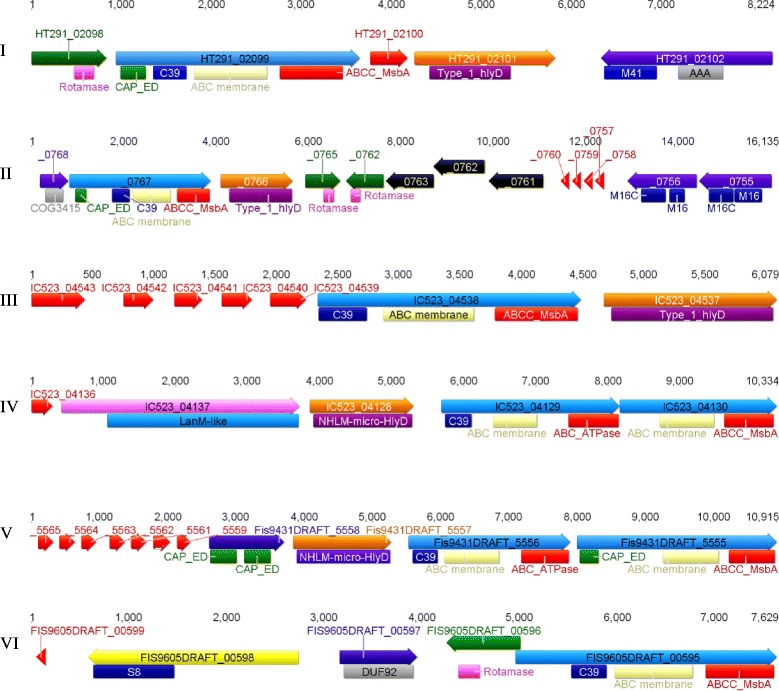
Examples of bacteriocin gene clusters from the Subsection V cyanobacteria. The six different groups (classified according to Wang *et al.* [34]) identified from the Subsection V cyanobacteria are represented. The group I gene cluster shown was identified from *W. intricata* UH strain HT-29-1; group II gene cluster shown was identidied from *M. repens* PCC 10914; the group III and IV gene clusters shown were identidied from *H. welwitschii* UH strain IC-52-3; the group V gene cluster shown was identified from *Fischerella* sp. PCC 9431 and the group VI gene cluster shown was identified from *Fischerella* sp. PCC 9605. Putative precursor genes are represented by a red arrow, HlyD genes are represented by orange arrow, SurA genes are represented by green arrow, ABC transporter genes are represented by green arrow, other modification enzymes are represented by purple arrow, S8 peptidase genes are represented by yellow arrow and LanM genes are represented by pink arrow. Domains involved in cyanobacterial bacteriocin production and modification are highlighted under each gene (domain names derived from the Conserved Domain Database [48]

From the 13 Subsection V cyanobacterial genomes analysed, a total of 116 bacteriocin gene clusters were identified (Table 3). The gene clusters were organised into the seven groups according to Wang et al. [34] (Fig. 4) based on the presence of the genes in the gene cluster, however, this study identified nine gene clusters which were unable to be classified (Table 3).

**Table 3:** Summary of bacteriocin biosynthetic gene clusters identified from Subsection V cyanobacteria, separated into groups according to Wang *et al.,* [34]

Organism	I	II	III	IV	V	VI	VII	Unclassified	Total
*W. intricata* UH strain HT-29-1	3	2	1	1	1	1	0	0	9
*H. welwitschii* UH strain IC-52-3	3	2	1	2	1	1	0	0	10
*Fischerella* sp. PCC 9339	3	2	1	0	1	1	0	1	9
*Fischerella* sp. PCC 9605	4	2	2	1	1	1	0	1	12
*Fischerella* sp. PCC 9431	4	1	1	1	1	1	0	0	9
*Fischerella* sp. JSC-11	3	1	2	1	0	1	0	0	8
*F. muscicola* SAG 1427-1	3	1	1	1	1	1	0	0	8
*F. muscicola* PCC 7414	3	1	2	0	0	1	0	1	8
*F. thermalis* PCC 7521	3	0	2	1	0	1	0	0	7
*Chlorogloeopsis* sp. PCC 9212	2	0	3	2	0	1	0	2	10
*C. fritschii* PCC 6912	2	0	3	2	0	1	0	2	10
*M. testarum* BC008	3	2	2	1	1	0	0	1	10
*M. repens* PCC 10914	2	1	0	1	0	1	0	1	6
Total:	38	15	21	14	7	12	0	9	116

The group I were the most abundant type identified from the Subsection V cyanobacterial genomes (Table 3). Six of the group I bacteriocin gene clusters contained a gene encoding M41 peptidases, which were not previously identified in other bacteriocin gene clusters. Genes encoding M16 peptidases (characteristic of the group II bacteriocin gene clusters) were only identified from the genomes of *M. repens* PCC 10914 and *Fischerella* sp. PCC 9605 (Additional file 6).

The second most abundant group of bacteriocin gene clusters was group III (Table 3). The group IV bacteriocin gene clusters, which contain the LanM-type genes, were identified in 11 of the Subsection V cyanobacterial genomes. The single LanM gene was identified in six genomes however, in two of these genomes (*Chlorogloeopsis* sp. PCC 9212 and *C. fritschii* PCC 6912), a single type 2 lantibiotic gene was identified clustered with the LanM gene (Additional file 6). The group IV bacteriocin gene cluster from *Fischerella* sp. PCC 9605 encodes an S8 peptidase, which according to Wang et al. [34], has only been previously identified in group VI bacteriocin gene clusters. No group VII bacteriocin gene clusters, containing the fused ABC transporter, were identified from the Subsection V cyanobacterial genomes.

Within each of the Subsection V cyanobacterial genomes, a single putative HetP-type precursor was identified from a group I bacteriocin gene cluster. Protein alignments of these putative precursors with previously identified HetP type precursors from Wang et al. [34] identified the leader peptide cleavage motif (KIxDLxYLEx_10_GG) (Additional file 6).

The putative DUF37-type precursors were also identified from all the Subsection V cyanobacterial genomes, however, none of these genes were clustered with other bacteriocin biosynthetic genes. Protein alignments of these putative precursors with previously identified DUF37 precursors identified by Wang et al. [34] enabled the identification of a putative leader peptide cleavage motif from the Subsection V cyanobacterial precursors (Additional file 6). These precursors do not demonstrate sequence similarity to any known proteins in the NCBI database, and therefore their function cannot be predicted at this time.

The N11P-type precursor peptide was identified in all of the Subsection V cyanobacterial genomes. However, the double glycine motif for peptide cleavage was unable to be identified in any of the precursor peptide sequences (Additional file 6). The NHLP-type precursor was only identified from *Fischerella* sp. PCC 9605. This putative precursor peptide was aligned with selected NHLP precursor peptides from *N. punctiforme* PCC 73102, and a conserved region near the peptide cleavage site (double glycine motif) was identified [87] (Additional file 6).

The wide distribution and range of bacteriocin gene clusters identified from the Subsection V cyanobacteria is consistent with previous reports from other cyanobacteria. Recent genome mining studies by Wang et al. [34] identified 145 bacteriocin gene clusters from 43 cyanobacteria, and Shih et al. [37] identified 358 bacteriocin gene clusters from 106 cyanobacterial genomes. Shih et al. [37] identified 23 bacteriocin (including lantipeptide) gene clusters from the five Subsection V cyanobacteria analysed, however, in this study, we identified 38 bacteriocin gene clusters from the same genomes. Wang et al. [34] identified M16 and S8 peptidase domains located within bacteriocin gene clusters. In this study, M41 peptidase domains, also known as FtsH peptidases, were identified within or adjacent to bacteriocin gene clusters encoded within the Subsection V cyanobacterial genomes. These membrane-anchored ATP-dependent peptidases suggest other cleavage sites, in addition to the double glycine motif, are located on the precursor peptides. Interestingly, the double glycine motif for peptide cleavage was unable to be identified within the N11P-type precursor peptide sequences, suggesting these precursor peptides may be nonfunctional. Out of the 51 putative precursors with similarity to known precursor types identified from the Subsection V cyanobacterial genomes, only two of these putative precursors were identified using antiSMASH; the remaining precursor peptides were identified using the BLAST program within the IMG JGI database. However, almost every bacteriocin gene cluster identified from the Subsection V cyanobacteria also encoded a large number of short peptide sequences either within or located at the ends of the bacteriocin gene clusters, in addition to the known precursor types. These short peptide sequences may encode precursor peptides for bacteriocin biosynthesis. Future functional characterisation of these putative precursor peptides will determine if these sequences are part of the bacteriocin gene clusters identified from the Subsection V cyanobacteria.

### Mycosporine-like amino acid (MAA) and scytonemin biosynthetic gene clusters

**Fig. 5:**
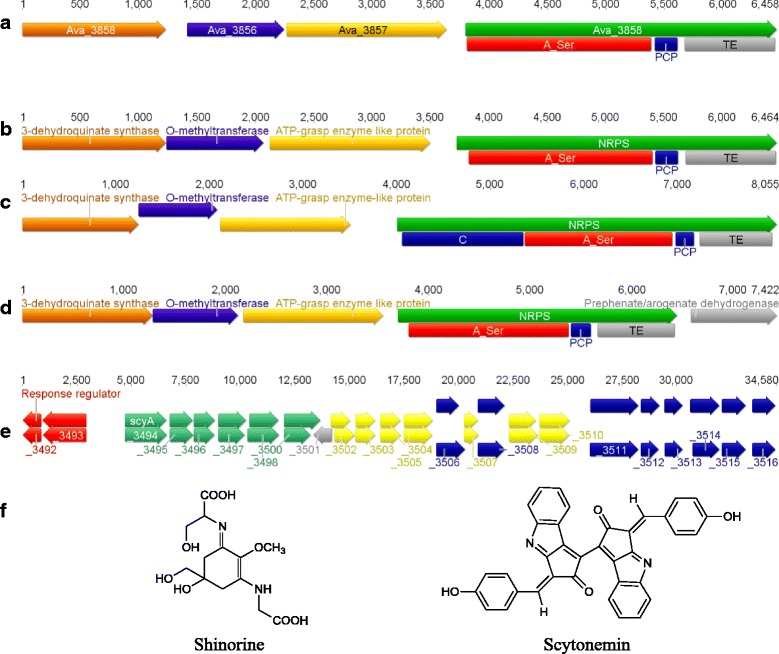
MAA and scytonemin biosynthetic gene clusters identified from Subsection V cyanobacteria. **a**) *mys* gene cluster from *Anabaena variabilis* ATCC 29413. **b**) *mys* gene cluster identified from *Fischerella muscicola* SAG 1427-1 and *Fischerella* sp*.* PCC 9339. **c**) *mys* gene cluster with C domain encoded in NRPS gene identifed from *Chlorogloeopsis* sp. PCC 9212, *Chlorogloeopsis fritschii* PCC 6912 and *Mastigocoleus testarum* BC008. **d**) *mys* with additional gene located downstream from NRPS gene identified from *Westiella intricata* UH strain HT-29-1, *Hapalosiphon welwitschii* UH strain IC-52-3 and *Fischerella* sp. PCC 9431. **e**) *scy* gene cluster from *Mastigocladopsis repens* PCC 10914 (Locus Tag: Mas10914DRAFT_xxxx) aligned with *scy* gene cluster from *Nodularia spumigena* CCY9414. Scytonemin core genes are represented by green arrows, regulatory proteins in red, aromatic amino acid biosynthetic genes are blue, other hypothetical genes are represented in yellow, and transposase gene is highlighted in silver arrow. **f**) Chemical structures of the MAA shinorine and scytonemin

Cyanobacteria are known to produce two different types of UV-absorbing molecules, MAAs and scytonemin, in order to protect the cells against either UV-B or UV-A radiation, respectively [88–92]. The *mys* biosynthetic gene cluster has previously been identified from three Subsection V cyanobacterial genomes, specifically *Fischerella* sp. PCC 9339 [37], *Fischerella* sp. PCC 9431 [38] and *C. fritschii* PCC 6912 [42]. In this study, we identified an additional five genomes harbouring the *mys* gene cluster, including *W. intricata* UH strain HT-29-1, *H. welwitschii* UH strain IC-52-3, *M. testarum* BC008, *F. muscicola* SAG 1427–1 and *Chlorogloeopsis* sp. PCC 9212 (Fig. 5). All eight *mys* gene clusters identified contain the three genes required for the biosynthesis of mycosporine-glycine, the precursor compound for shinorine biosynthesis. All eight gene clusters also encode an NRPS-like enzyme proposed to activate serine (Additional file 7) and suggests all eight Subsection V cyanobacteria are capable of biosynthesising the MAA shinorine, identical to the pathway in *A. variabilis* ATCC 29413, as reported by Balskus and Walsh [88]. However, the domain organisation of the NRPS-like enzyme varies between the cyanobacterial strains. The majority of the identified gene clusters encode the domain organisation A-PCP-TE (Fig. 5a), identical to the characterised NRPS-like enzyme from *A. variabilis* ATCC 29413. However, three cyanobacterial strains (*C. fritschii* PCC 6912, *Chlorogloeopsis* sp. PCC 9212 and *M. testarum* BC008) all also encode a C domain at the N-terminus (Fig. 5b). An additional gene was identified downstream from the NRPS-like enzyme in three Subsection V cyanobacterial strains, specifically in *W. intricata* UH strain HT-29-1, *H. welwitschii* UH strain IC-52-3 and *Fischerella* sp. PCC 9431 (Fig. 5c). The encoded protein belongs to the prephenate/arogenate dehydrogenase family, which catalyse the conversion of prephenate to tyrosine in the shikimate pathway. While it is known that a wide variety of MAA analogues are biosynthesised, the effect of this fifth encoded gene (if any) remains unknown. However, transcription analysis revealed that this fifth gene is co-transcribed with the *W. intricata* UH strain HT-29-1 *mys* gene cluster in response to UV-B exposure (Micallef, ML. unpublished data).

Lastly, the scytonemin (*scy*) biosynthetic gene cluster was only identified within the *M. repens* PCC 10914 genome (Fig. 5d). The 32.9 kb gene cluster is similar to the *scy* gene cluster from *N. spumigena* CCY9414 [91], however the gene cluster from *M. repens* PCC 10914 encodes a transposase downstream of *scyF*. The presence of the *scy* gene cluster within the *M. repens* PCC 10914 genome suggests this organism is capable of biosynthesising scytonemin in order to protect the cells against UV-A radiation.

### Identification of hydrocarbon biosynthetic gene clusters

**Fig. 6:**
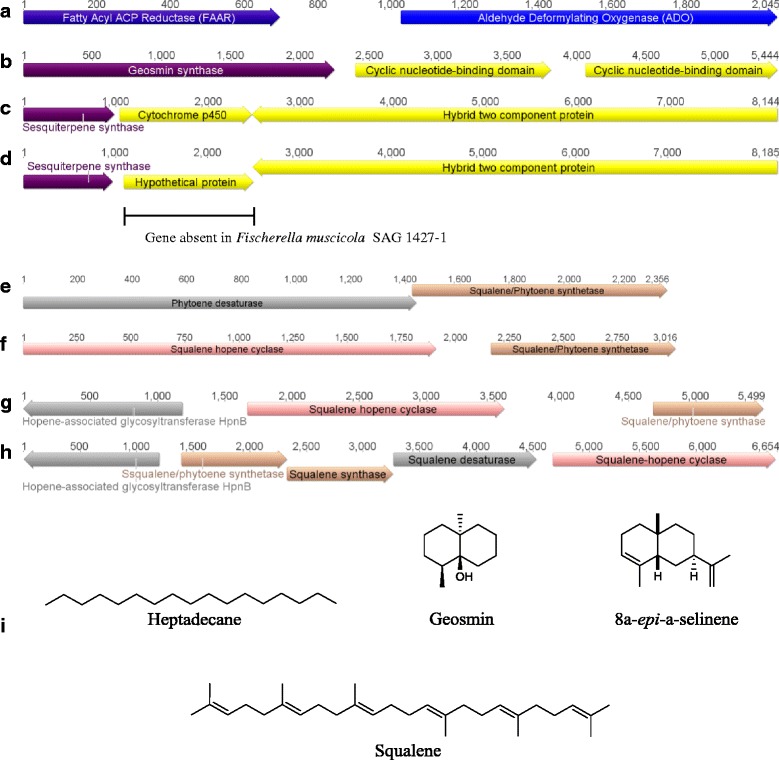
Additional gene clusters identified from Subsection V cyanobacterial genomes. **a** Hydrocarbon biosynthetic gene cluster identified from all the Subsection V cyanobacterial genomes. **b** Geosmin gene cluster identified from *W. intricata* UH strain HT-29-1, *H. welwitschii* UH strain IC-52-3, *Fischerella* sp. PCC 9431 and *F. muscicola* SAG 1427–1. **c** Sesquiterpene gene cluster from *Fischerella* sp. JSC-11, *F. thermalis* PCC 7521, *F. muscicola* PCC 7414and *Fischerella* sp. PCC 9605. **d** Sesquiterpene gene cluster with hypothetical protein instead of cytochrome p450 identified from *Fischerella* sp. PCC 9339, *W. intricata* UH strain HT-29-1, *H. welwitschii* UH strain IC-52-3 and *Fischerella* sp. PCC 9431. **e** Squalene gene cluster encoding phytoene desaturase identified in all Subsection V cyanobacterial genomes. **f** Squalene gene cluster encoding squalene hopene cyclase identified in all Subsection V cyanobacterial genomes except *M. testarum* BC008 and *M. repens* PCC 10914. **g** Squalene gene cluster encoding squalene hopene and hopene-assocciated glycosyltransferase identified in *Chlorogloeopsis* sp. PCC 9212, *C. fritschii* PCC 6912 and *M. repens* PCC 10914 (squalene synthase gene not clustered in *M. repens* PCC 10914). **h**) Unique squalene gene cluster identified from *M. testarum* BC008 genome. **i**) Chemical structures of heptadecane, geosmin, 8a-*epi*-selinene and squalene

Cyanobacteria have the unique ability to produce hydrocarbons from fatty acids, including heptadecane and methylheptadecane, which have potential diesel fuel applications [12]. The hydrocarbon biosynthetic gene cluster contains two genes, FAAR and ADO, for the biosynthesis of alkanes from fatty acids [69]. The FAAR/ADO pathway has previously been identified in all 13 Subsection V cyanobacterial genomes (Fig. 6a) [12, 43, 69]. All the Subsection V cyanobacterial strains analysed contain only the FAAL/ADO gene cluster; the OLS pathway was not identified from any Subsection V cyanobacterial genomes, which is consistent with the observation that all analysed cyanobacterial genomes encode only one type of hydrocarbon biosynthetic gene cluster [12].

Hydrocarbon composition has been characterised for four of the Subsection V cyanobacteria. Whilst *W. intricata* UH strain HT-29-1 is only able to biosynthesis heptadecane, *H. welwitschii* UH strain IC-52-3, *F. muscicola* PCC 7414 and *C. fritschii* PCC 6912 are capable of biosynthesising heptadecanes and methylheptadecanes [12]. Heptadecane is the most commonly observed hydrocarbon from cyanobacteria, whilst branched alkanes, such as methylheptadecanes, are also only observed from cyanobacteria encoding the FAAR/ADO pathway [12]. Since hydrocarbon biosynthetic gene clusters have been identified from a broad range of cyanobacteria, it has been suggested that an unknown selective pressure is forcing these organisms to maintain either pathway within their genomes [12].

### Identification of terpene biosynthetic gene clusters

A number of terpene biosynthetic gene clusters were identified from the Subsection V cyanobacteria (Fig. 6b-h) (Additional file 8). The 5.4 kb gene cluster encoding the biosynthesis of geosmin (encoding geosmin synthase and two cyclic nucleotide-binding domain proteins) was identified in four of the 13 Subsection V cyanobacterial genomes, specifically *W. intricata* UH strain HT-29-1, *H. welwitschii* UH strain IC-52-3, *Fischerella* sp. PCC 9431 and *F. muscicola* SAG 1427–1 (Fig. 6b). The gene encoding geosmin synthase displays approximately 90 % amino acid sequence similarity with geosmin synthase from *N. punctiforme* PCC 7120 [71]. Characterisation of the geosmin synthase from *N. punctiforme* PCC 73102 by Giglio et al. [71] revealed that the protein catalyses the biosynthesis of geosmin, as well as germacradienol, germacrene D, octalin and (*E*)-nerolidol. The gene cluster encoding 2-methylisoborneol, however, was not identified from any of the Subsection V cyanobacterial genome sequences.

A 8.2 kb sesquiterpene biosynthetic gene cluster, encoding three proteins (a sesquiterpene synthase, a cytochrome p450 and a putative hybrid two-component protein), was identified in four Subsection V cyanobacterial genomes (*Fischerella* sp. JSC-11, *F. thermalis* PCC 7521, *F. muscicola* PCC 7414 and *Fischerella* sp. PCC 9605) (Fig. 6c). This gene cluster demonstrates approximately 78 % amino acid sequence similarity with the 8a-*epi*-α-selinene biosynthetic gene cluster from *N. punctiforme* PCC 7120 [70]. However, in four additional cyanobacterial genomes, a similar sesquiterpene biosynthetic gene cluster was identified, in which a gene encoding a hypothetical protein related to 2-polyprenyl-6-methoxyphenol hydroxylase and FAD-depended oxidoreductases (COG0654) was identified in place of the gene encoding a cytochrome p450 (*Fischerella* sp. PCC 9339, *W. intricata* UH strain HT-29-1, *H. welwitschii* UH strain IC-52-3 and *Fischerella* sp. PCC 9431) (Fig. 6d). The presence of this alternative gene in the sesquiterpene gene cluster prevents the prediction of the encoded natural product. In *F. muscicola* SAG 1427–1, only genes encoding sesquiterpene synthetase and a hybrid two-component protein were identified.

Two additional terpene gene clusters were identified from the Subsection V cyanobacterial genomes using antiSMASH. Genes encoding a phytoene/squalene synthetase and a phytoene desaturase (COG3349) were clustered together in all Subsection V cyanobacterial genomes (Fig. 6e). The other terpene gene cluster found in 11 Subsection V cyanobacterial genomes encodes a phytoene/squalene synthetase and a squalene-hopene cyclase (Fig. 6f). The encoded phytoene/squalene synthetases and squalene-hopene cyclase from the Subsection V cyanobacteria demonstrate approximately 80 % amino acid sequence similarity to the recently characterised squalene synthase and squalene hopene cyclase from *Synechocystis* sp. PCC 6803 [93]. In three genomes (*Chlorogloeopsis* sp. PCC 9212, *C. fritschii* PCC 6912 and *M. repens* PCC 10914), a hopene-associated glycosyltransferase *hpnB* (COG1215) gene was identified downstream from the squalene-hopene cyclase gene (Fig. 6g). The gene cluster from *M. repens* PCC 10914, however, does not contain the phytoene/squalene synthetase gene, although the gene is located within the genome.

A 6.6 kb terpene gene cluster from *M. testarum* BC008 is distinct from the other Subsection V cyanobacterial terpene gene clusters (Fig. 6h). In *M. testarum* BC008, the terpene gene cluster contains a hopene-associated glycosyltransferase *hpnB* (COG1215) gene, two phytoene/squalene synthetase genes, a phytoene desaturase gene, and a squalene-hopene cyclase gene. Furthermore *M. testarum* BC008 also encodes an additional squalene synthetase gene in the genome.

### Identification of hapalindole and welwitindolinone biosynthetic gene clusters

A major class of secondary metabolites produced exclusively, to date, by the Subsection V cyanobacteria are the hapalindole family of natural products. A putative gene cluster encoding the biosynthesis of the hapalindoles and welwitindolinones has been published separately by our research group and Hillwig et al. [39–41]. Breifly, the presence of genes encoding methyltransferases (*welM1-3*), specific oxygenases (*welO11-19*) and regulation proteins (*welR3*) within the gene cluster suggests *W. intricata* UH strain HT-29-1, *H. welwitschii* UH strain IC-52-3, *Fischerella* sp. PCC 9431 and *F. muscicola* SAG 1427–1 encodes the *wel* gene cluster [41]. Characterisation of WelM1, responsible for the biosynthesis of *N-*methylwelwitindolinone C isothiocyanate, confirmed the *wel* gene cluster is responsible for welwitindolinone biosynthesis [39]. The gene cluster from *Fischerella* sp. PCC 9339 lacks the gene *ambP3* (encoding the prenyltransferase responsible for ambiguine biosynthesis) and the genes characteristic of the *wel* gene cluster, suggesting the gene cluster from *Fischerella* sp. PCC 9339 encodes the biosynthesis of the hapalindoles [41].

## Conclusion

In this study, all 11 publically available Subsection V cyanobacterial genomes, together with our draft genomes of *W. intricata* UH strain HT-29-1 and *H. welwitschii* UH strain IC-52-3 were mined for their genetic potential to produce secondary metabolites. In this study, we were able to identify a putative gene cluster for hapalosin from the producing organism *H. welwitschii* UH strain IC-52-3. However, through genome mining, additional Subsection V cyanobacteria were identified to also encode the *hap* gene cluster. This study has also identified a wide range of biosynthetic gene clusters from the Subsection V cyanobacteria, including orphan NRPS/PKS gene clusters, PRPS gene cluster (including cyanobactin, microviridin and bacteriocin gene clusters), MAA and scytonemin gene clusters for UV-absorbing compounds and terpene gene clusters for geosmin, sesquiterpene and squalene biosynthesis. Through genome mining, the distribution and diversity of secondary metabolite biosynthesis in the Subsection V cyanobacteria has been revealed.

### Availability of supporting data

The draft genome sequences of *W. intricata* UH strain HT-29-1 and *H. welwitschii* UH strain IC-52-3 reported in this article were deposited as data sets in the US Department of Energy (DOE) Joint Genome Institute (JGI) Integrated Microbial Genomes (IMG) repository, under the Taxon ID 2529292565 [https://img.jgi.doe.gov/cgi-bin/er/main.cgi?section=TaxonDetail&page=taxonDetail&taxon_oid=2529292565] and 2529292566 [https://img.jgi.doe.gov/cgi-bin/er/main.cgi?section=TaxonDetail&page=taxonDetail&taxon_oid=2529292566], respectively. All other data was obtained from the US DOE JGI IMG database with the following Taxon ID *Fischerella* sp. PCC 9339 (2516653082), *Fischerella* sp. PCC 9431 (2512875027) *Fischerella* sp. JSC-11 (2505679024), *Fischerella* sp. PCC 9605 (2516143000), *F. muscicola* PCC 7414 (2548876996), *F. muscicola* SAG 1427–1 (2548876995), *F. thermalis* PCC 7521 (2548876998), *M. repens* PCC 10914 (2517093042), *C. fritschii* PCC 6912 (2551306142) and *Chlorogloeopsis* sp. PCC 9212 (2548877023). *M. testarum* BC008 genomic data was obtained from the National Centre for Biotechnology Information (NCBI) with GenBank Assembly Accession 000472885.1.

## Additional files

Additional file 1:
**Specific and degenerative primers used to close gaps in orphan NRPS/PKS gene sequences and identify A-KR didomain.** (DOCX 16 kb) Additional file 2:
***W. intricata*** **UH strain HT-29-1 and** ***H. welwitschii*** **UH strain IC-52-3 102 housekeeping genes, COGs.** (XLSX 118 kb) Additional file 3: Table S1. Hapalosin biosynthetic gene cluster, Table S2: Adenylation domain binding pockets from *hap* gene cluster (DOCX 22 kb) Additional file 4:
**Complete orphan NRPS/PKS gene clusters identified from the Subsection V cyanobacterial genomes.** The genomes in which the orphan gene clusters were identified are stated to the left of each cluster. Fragments of gene clusters, or differences from the gene cluster shown are noted in the image. NRPS/PKS genes are represented by green arrows. Additional genes that may be involved in biosynthesis are represented by blue arrows. Genes encoding hypothetical proteins and transposases are represented by silver arrows. (PDF 307 kb) Additional file 5:
**Hapalosin and orphan NRPS/PKS gene clusters.** (DOCX 94 kb) Additional file 6:
**Bacteriocin gene cluster and precursor analysis.** (DOCX 103 kb) Additional file 7:
**A domain binding pocket of NRPS-like enzyme identified in** ***mys*** **gene clusters from Subsection V cyanobacteria.** (DOCX 18 kb) Additional file 8:
**Terpene gene clusters.** (DOCX 20 kb)
