# Supplementary material for: Genome mining for natural product biosynthetic gene clusters in the Subsection V cyanobacteria
Source: BMC Genomics. 2015 Sep 3;16(1):669. doi: 10.1186/s12864-015-1855-z (PMC4558948; doi:10.1186/s12864-015-1855-z)
Supplement: Additional file 1: — Specific and degenerative primers used to close gaps in orphan NRPS/PKS gene sequences and identify A-KR didomain. (DOCX 16 kb) [file 12864_2015_1855_MOESM1_ESM.docx]

| **Additional file 1: Specific and degenerative primers used to close gaps in orphan NRPS/PKS gene sequences and identify A-KR didomain** | | |
| --- | --- | --- |
| Primer name: | Primer sequence^£^: | Melting temperature (°C): |
| HT1767F | CTCAATTGATATCTCGAATGC | 58.8 |
| HT1767R | CTATTTCATTGATGCTGTTTTC | 57.9 |
| ICScaffold44-90F1 | GAAATTATTTTGAGCCAGATTG | 59.2 |
| ICScaffold44-90F2 | GACACTCTCTCCGCTAATTG | 59.1 |
| ICScaffold44-90F3 | CTTTGTACTGGGGAAAAATC | 57.6 |
| ICScaffold44-90R1 | GTTGGTAAATCTTCCTCTTTG | 56.9 |
| ICScaffold44-90R2 | GACATAGCGGTAAGTAGTAG | 50.7 |
| AKR1F | TTTTGGGGTTGTATTTTGGGNGGNTT | 70.5 |
| AKR4R | ATCACCAGT(A/G)TT(A/G)AACCANCC(A/G)TC | 62.9 |
| AKR4F | GATGGTTGGTTTAA(C/T)ACNGGNGA | 61.4 |
| AKR5R | ACCAATACCACCNA(A/G)NCCNCC | 60.5 |
| ^£^N indicates any nucleotide. | | |
