# Supplementary material for: Genome mining for natural product biosynthetic gene clusters in the Subsection V cyanobacteria
Source: BMC Genomics. 2015 Sep 3;16(1):669. doi: 10.1186/s12864-015-1855-z (PMC4558948; doi:10.1186/s12864-015-1855-z)
Supplement: Additional file 3: Table S1. — Hapalosin biosynthetic gene cluster, Table S2: Adenylation domain binding pockets from hap gene cluster (DOCX 22 kb) [file 12864_2015_1855_MOESM3_ESM.docx]

| **Additional file 3, Table 1: *hap* biosynthetic gene cluster** | | | | | | |
| --- | --- | --- | --- | --- | --- | --- |
| Protein Name: | Length (aa): | % similarity^£^ | Proposed function: | Top BLASTp results: | % Identity /Similarity^€^ | Accession No. |
| HapA | 708 | 99.3 | Initiation Module: AS-ACP | HctA, *Moorea producens* | 54/71 | AAY42393 |
| HapB | 1,979 | 99.4 | PKS:  KS-AT-CM-KR-PCP | HctD, *Moorea producens* | 46/63 | AAY42396 |
| HapC | 2,348 | 99.5 | NRPS:  C-A-KR-PCP | HctF, *Moorea producens* | 47/63 | AAY42398 |
| HapD | 1,526 | 99.2 | NRPS:  C-A-NM-PCP | ApdB, *Anabaena* sp. 90 | 64/76 | YP_006996377 |
| HapE | 1,824 | 99.1 | PKS:  KS-AT-KR-ACP-TE | JamP, *Moorea producens* | 56/72 | AAS98787 |
| ^£^Amino acid similarity between Hap proteins from *Hapalosiphon welwitschii* UH strain IC-52-3, *Westiella intricata* UH strain HT-29-1 and *Fischerella* sp. PCC 9431.  ^€^Based on *Hapalosiphon welwitschii* UH strain IC-52-3. | | | | | | |

| **Additional file 3, Table 2: A domain binding pockets from *hap* gene cluster** | | | | | | | | | | |
| --- | --- | --- | --- | --- | --- | --- | --- | --- | --- | --- |
| Protein | Binding pocket amino acids | | | | | | | | Substrate | Reference |
|  | 235 | 236 | 239 | 278 | 299 | 301 | 322 | 330 |  |  |
| HctE | V | G | V | W | L | A | L | F | 2-oxoisovaleric acid | [[1](#_ENREF_1)] |
| HctF | V | G | V | W | L | A | L | F | 2-oxoisovaleric acid | [[1](#_ENREF_1)] |
| HapC^€^ | V | G | V | W | L | A | L | F | 2-oxoisovaleric acid^£^ | This study |
| HapD^€^ | D | A | W | T | I | A | A | V | Phenylalanine^Ʊ^ | This study |
| GrsA | D | A | W | T | I | A | A | I | Phenylalanine | [[2](#_ENREF_2)] |
| ^€^Identical binding pocket between *Hapalosiphon welwitschii* UH strain IC-52-3, *Westiella intricata* UH strain HT-29-1 and *Fischerella* sp. PCC 9431.  ^£^Proposed substrates based on identical binding pocket residues with HctE and HctF from *hct* gene cluster and hapalosin structure  ^Ʊ^Proposed substrate based on near identical binding pocket residues of GrsA and hapalosin structure | | | | | | | | | | |

1. Ramaswamy AV, Sorrels CM, Gerwick WH: **Cloning and biochemical characterization of the hectochlorin biosynthetic gene cluster from the marine cyanobacterium *Lyngbya majuscula***. *J Nat Prod* 2007, **70**(12):1977-1986.

2. Challis G, Ravel J, Townsend C: **Predictive, structure-based model of amino acid recognition by nonribosomal peptide synthetase adenylation domains**. *Chem Biol* 2000, **7**(3):211 - 224.
