# Supplementary material for: Genome mining for natural product biosynthetic gene clusters in the Subsection V cyanobacteria
Source: BMC Genomics. 2015 Sep 3;16(1):669. doi: 10.1186/s12864-015-1855-z (PMC4558948; doi:10.1186/s12864-015-1855-z)
Supplement: Additional file 4: — Complete orphan NRPS/PKS gene clusters identified from the Subsection V cyanobacterial genomes. The genomes in which the orphan gene clusters were identified are stated to the left of each cluster. Fragments of gene clusters, or differences from the gene cluster shown are noted in the image. NRPS/PKS genes are represented by green arrows. Additional genes that may be involved in biosynthesis are represented by blue arrows. Genes encoding hypothetical proteins and transposases are represented by silver arrows. (PDF 307 kb) [file 12864_2015_1855_MOESM4_ESM.pdf]

Additional file 4: Complete orphan NRPS/PKS gene clusters identified from the Subsection V cyanobacterial genomes.

Cluster 1:
CS PCC 9212
CF PCC 6912

Cluster 2:
MR PCC 10914

Cluster 3:
FS PCC 9605

Cluster 4:
FS PCC 9605

Cluster 5:
FS PCC 9339

Cluster 6:
WI HT-29-1,
FS PCC 9431
and
HW IC-52-3

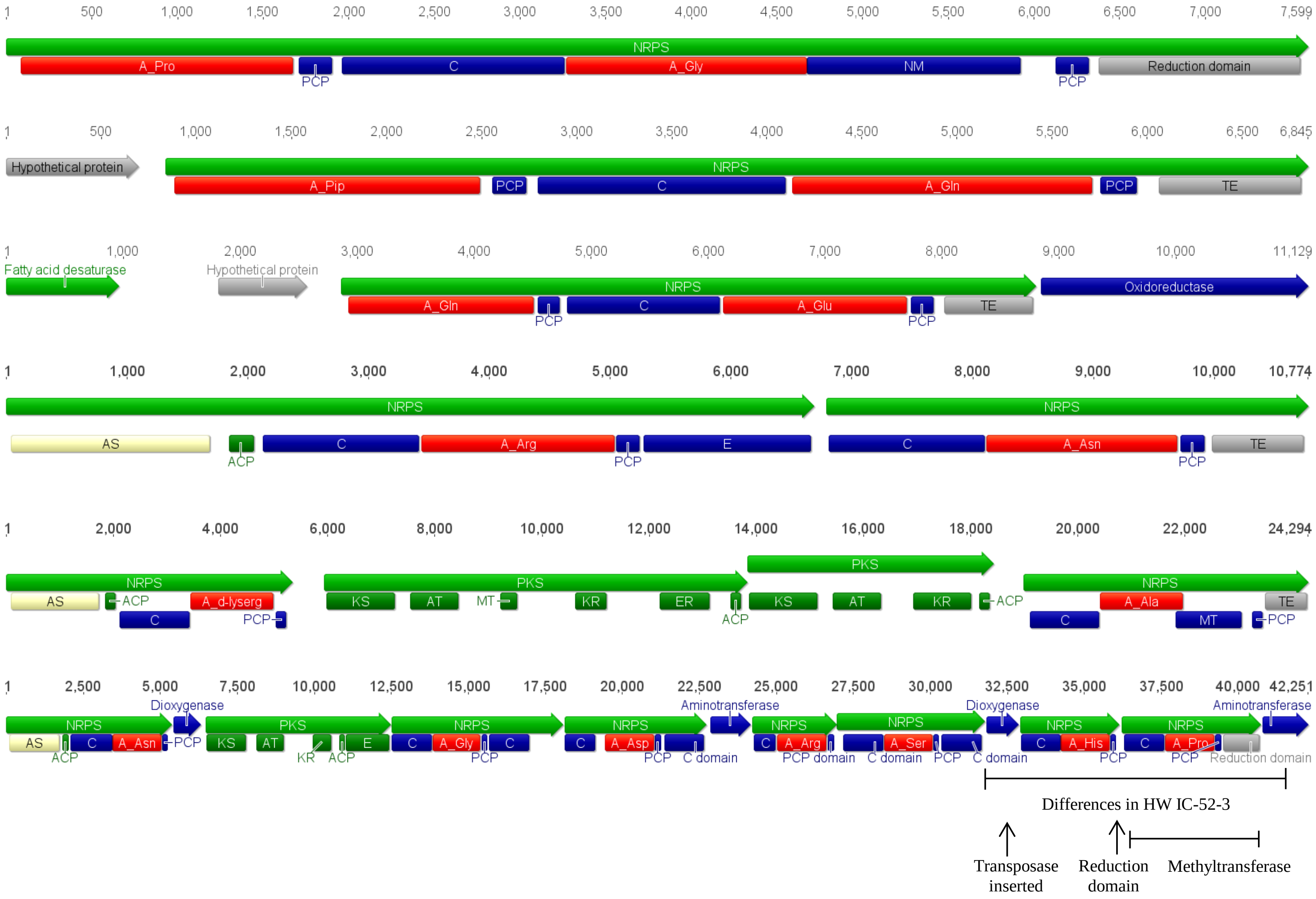

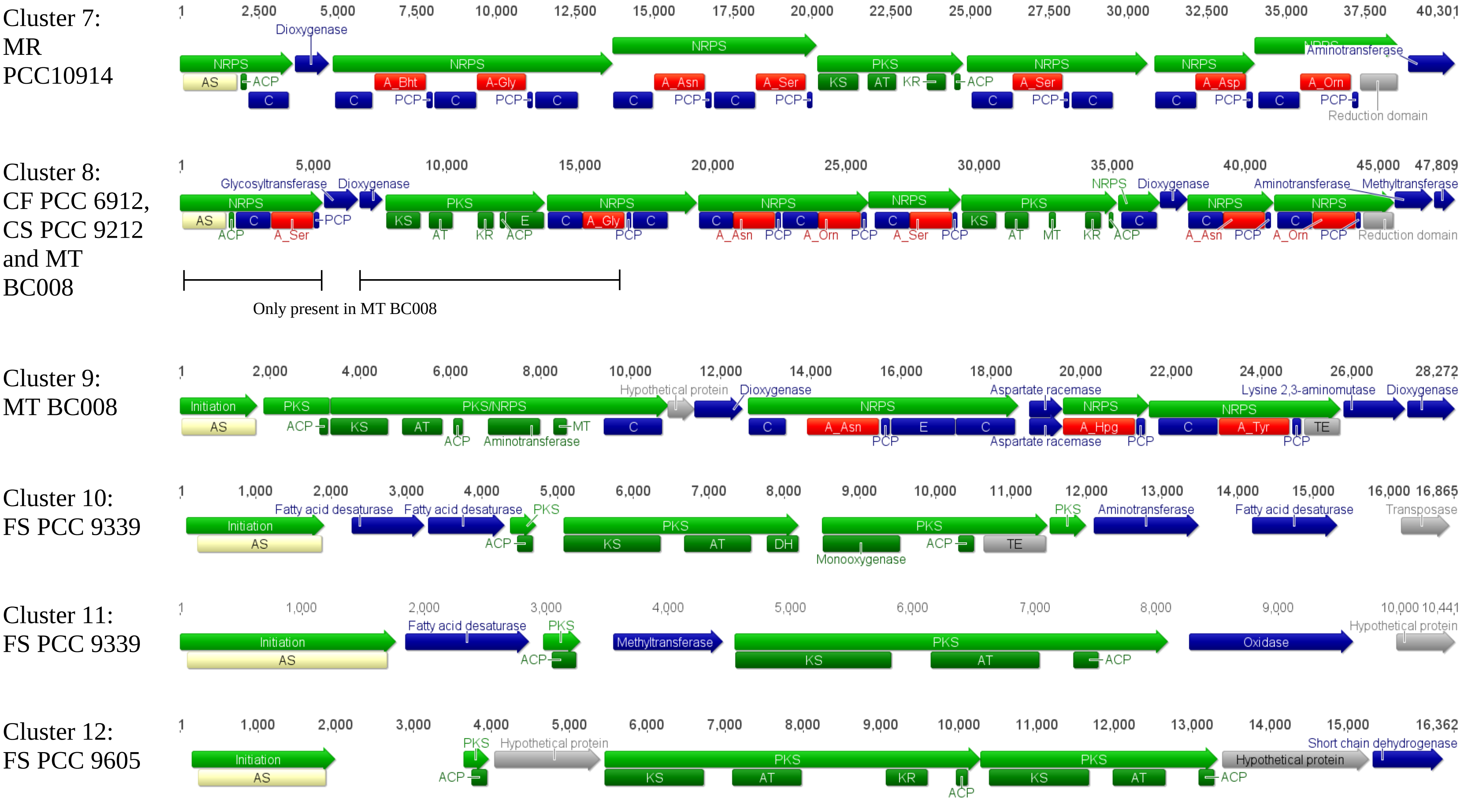

Cluster 7:
MR PCC10914
Dioxygenase
NRPS
NRPS
NRPS
PKS
NRPS
NRPS
Aminotransferase
AS
ACP
A_Bht
A-Gly
A_Asn
A_Ser
KS
AT
KR
ACP
A_Ser
A_Asp
A_Orn
C
PCP
Reduction domain
Cluster 8:
CF PCC 6912,
CS PCC 9212
and MT
BC008
Glycosyltransferase
Dioxygenase
NRPS
PKS
NRPS
NRPS
NRPS
PKS
NRPS
Dioxygenase
NRPS
Aminotransferase
NRPS
Methyltransferase
AS
ACP
A_Ser
PCP
KS
AT
KR
ACP
E
A_Gly
A_Asn
A_Orn
MT
Reduction domain
Only present in MT BC008
Cluster 9:
MT BC008
Initiation
PKS
PKS/NRPS
Hypothetical protein
Dioxygenase
NRPS
Aspartate racemase
NRPS
NRPS
Lysine 2,3-aminomutase
Dioxygenase
AS
ACP
KS
AT
MT
Aminotransferase
A_Asn
E
A_Hpg
A_Tyr
TE
PCP
Cluster 10:
FS PCC 9339
Initiation
Fatty acid desaturase
Fatty acid desaturase
PKS
PKS
PKS
PKS
Aminotransferase
Fatty acid desaturase
Transposase
AS
ACP
KS
AT
DH
Monooxygenase
TE
Cluster 11:
FS PCC 9339
Initiation
Fatty acid desaturase
PKS
Methyltransferase
PKS
Oxidase
Hypothetical protein
AS
ACP
KS
AT
Cluster 12:
FS PCC 9605
Initiation
PKS
Hypothetical protein
PKS
PKS
Hypothetical protein
Short chain dehydrogenase
AS
ACP
KS
AT
KR

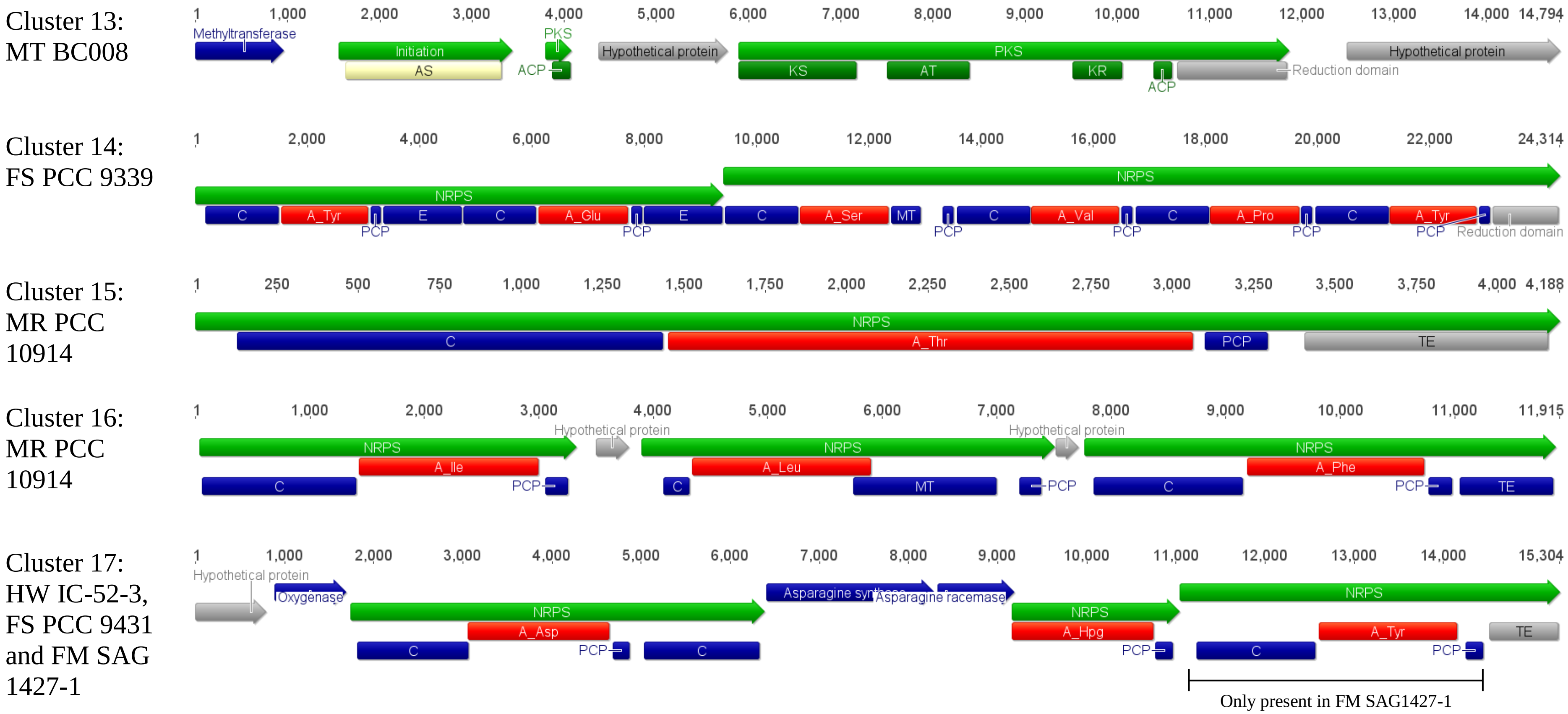
Cluster 13: MT BC008
1
1,000
2,000
3,000
4,000
5,000
6,000
7,000
8,000
9,000
10,000
11,000
12,000
13,000
14,000
14,794
Methyltransferase
Initiation
AS
PKS
ACP
Hypothetical protein
PKS
KS
AT
KR
ACP
Reduction domain
Hypothetical protein
Cluster 14: FS PCC 9339
1
2,000
4,000
6,000
8,000
10,000
12,000
14,000
16,000
18,000
20,000
22,000
24,314
NRPS
NRPS
C
A_Tyr
PCP
E
C
A_Glu
PCP
E
C
A_Ser
MT
PCP
C
A_Val
PCP
C
A_Pro
PCP
C
A_Tyr
PCP
Reduction domain
Cluster 15: MR PCC 10914
1
250
500
750
1,000
1,250
1,500
1,750
2,000
2,250
2,500
2,750
3,000
3,250
3,500
3,750
4,000
4,188
NRPS
C
A_Thr
PCP
TE
Cluster 16: MR PCC 10914
1
1,000
2,000
3,000
4,000
5,000
6,000
7,000
8,000
9,000
10,000
11,000
11,915
NRPS
A_Ile
C
PCP
Hypothetical protein
NRPS
A_Leu
C
MT
PCP
Hypothetical protein
NRPS
A_Phe
C
PCP
TE
Cluster 17: HW IC-52-3, FS PCC 9431 and FM SAG 1427-1
1
1,000
2,000
3,000
4,000
5,000
6,000
7,000
8,000
9,000
10,000
11,000
12,000
13,000
14,000
15,304
Hypothetical protein
Oxygenase
NRPS
A_Asp
C
PCP
C
Asparagine syn
Asparagine racemase
NRPS
A_Hpg
PCP
NRPS
C
A_Tyr
PCP
TE
Only present in FM SAG1427-1

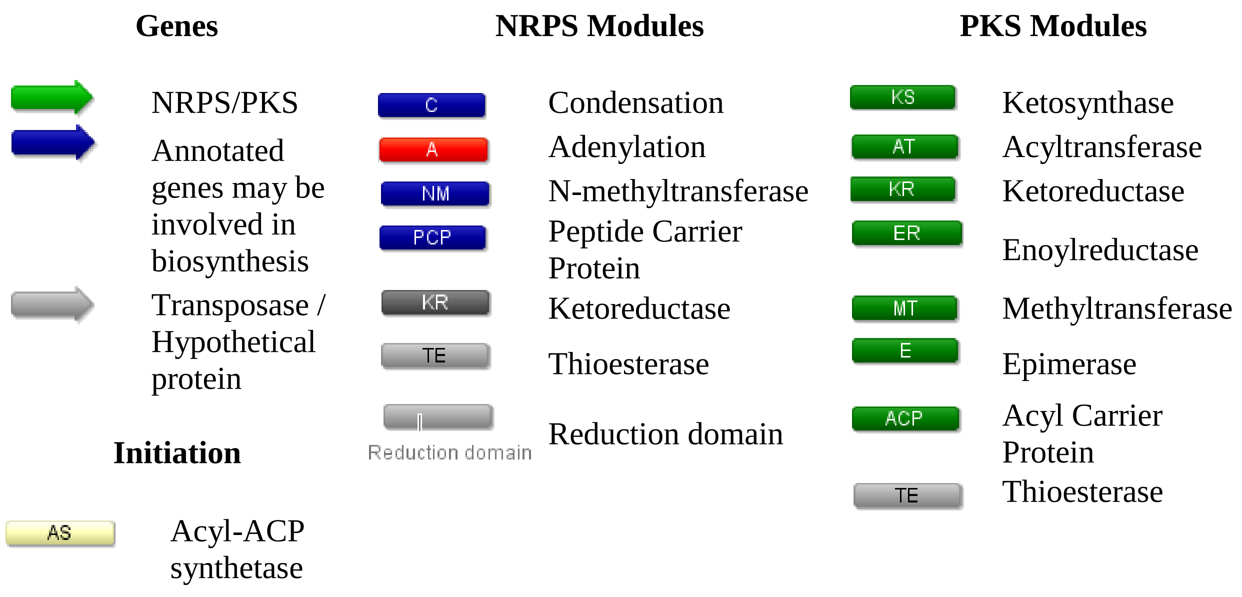

Additional file 4: Complete orphan NRPS/PKS gene clusters identified from the Subsection V cyanobacterial genomes. The genomes in which the orphan gene clusters were identified are stated to the left of each cluster (WI HT-29-1: *W. intricata* UH strain HT-29-1, HW IC-52-3: *H. welwitschii* UH strain IC-52-3, FS PCC 9431: *Fischerella* sp. PCC 9431, FS PCC 9399: *Fischerella* sp. PCC 9339, FM SAG 1427-1: *Fischerella muscicola* SAG 1427-1, CF PCC 6912: *Chlorogloeopsis fritschii* PCC 6912, CS PCC 9212: *Chlorogloeopsis* sp. PCC 9212, MT BC008: *Mastigocoleus testarum* BC008. MR PCC 10914: *Mastigocladopsis repens* PCC 10914, FS PCC 9605: *Fischerella* sp. PCC 9605). Fragments of gene clusters, or differences from the gene cluster shown are noted in the image.
