# Supplementary material for: Genome mining for natural product biosynthetic gene clusters in the Subsection V cyanobacteria
Source: BMC Genomics. 2015 Sep 3;16(1):669. doi: 10.1186/s12864-015-1855-z (PMC4558948; doi:10.1186/s12864-015-1855-z)
Supplement: Additional file 5: — Hapalosin and orphan NRPS/PKS gene clusters. (DOCX 94 kb) [file 12864_2015_1855_MOESM5_ESM.docx]

**Additional file 5: Hapalosin and orphan NRPS/PKS gene clusters**

| Hapalosin gene cluster: | | | | | | | | | | |
| --- | --- | --- | --- | --- | --- | --- | --- | --- | --- | --- |
| Gene: | Proposed function: | Domain organisation: | Subsection V cyanobacteria | | | | | | A domain binding pocket | Proposed amino acid |
|  |  |  | *Westiella intricata* UH strain HT-29-1 | | *Hapalosiphon welwitschii* UH strain IC-62-3 | | *Fischerella* sp. PCC 9431 | |  |  |
|  |  |  | Locus Tag: | Size (aa) | Locus Tag: | Size (aa) | Locus Tag: | Size (aa) |  |  |
| *hapA* | PKS | AS-ACP | HT291_04467 | 708 | IC523_04982 | 708 | Fis9431DRAFT_5403 | 708 |  |  |
| *hapB* | PKS | KS-AT-CM-KR-ACP | HT291_04468 | 1,979 | IC523_04981 | 1,979 | Fis9431DRAFT_5401 | 1,981 |  |  |
| *hapC* | NRPS/PKS | C-A-KR-PCP | HT291_04469 | 2,348 | IC523_04980 | 2,348 | Fis9431DRAFT_5400 | 2,348 | VGVWLALF | 2-oxoisovaleric acid |
| *hapD* | NRPS | C-A-MT-PCP | HT291_04470 | 1,526 | IC523_04979 | 1,526 | Fis9431DRAFT_5399 | 1,526 | DAWTIAAV | Phenylalanine |
| *hapE* | PKS | KS-AT-KR-ACP-TE | HT291_04471 | 1,824 | IC523_04978 | 1,824 | Fis9431DRAFT_5398 | 1,818 |  |  |

Orphan NRPS/PKS gene clusters:

Abbreviations: WI HT-29-1: *W. intricata* UH strain HT-29-1, HW IC-52-3: *H. welwitschii* UH strain IC-52-3, FS PCC 9431: *Fischerella* sp. PCC 9431, FS PCC 9399: *Fischerella* sp. PCC 9339, FM SAG 1427-1: *F. muscicola* SAG 1427-1, CF PCC 6912: *Chlorogloeopsis fritschii* PCC 6912, CS PCC 9212: *Chlorogloeopsis* sp. PCC 9212, MT BC008: *M. testarum* BC008. MR PCC 10914: *Mastigicladopsis repens* PCC 10914, FS PCC 9605: *Fischerella* sp. PCC 9605, FS JSC-11: *Fischerella* sp. JSC-11, FM PCC 7414: *Fischerella muscicola* PCC 7414, FT PCC 7521: *Fischerella thermalis* PCC 7521.

| Cluster 1: Orphan NRPS: | | | | | |
| --- | --- | --- | --- | --- | --- |
| Proposed function: | Domain organisation: | Subsection V cyanobacteria: | | A domain binding pocket: | Proposed amino acid: |
|  |  | CS PCC 9212 | CF PCC 6912 |  |  |
| NRPS | A-PCP-C-A-MT-PCP-Red | UYEDRAFT_04865 | UYCDRAFT_01991 | A1: DVQFIAHVVK  A2: DIIQLGVVWK | A1: Pro (100%)  A2: Gly (80%) |

| Cluster 2: Orphan NRPS: | | | | |
| --- | --- | --- | --- | --- |
| Proposed function: | Domain organisation: | MR PCC 10914 | A domain binding pocket: | Proposed amino acid: |
| Hypothetical protein |  | Mas10914DRAFT_0401 |  |  |
| NRPS | A-PCP-C-A-PCP-TE | Mas10914DRAFT_0400 | A1: SSRDVSVGVK  A2: DPWATGCIDK | Pip (30%)  Gln (60%) |

| Cluster 3: Orphan NRPS: | | | | | |
| --- | --- | --- | --- | --- | --- |
| Proposed function: | Domain organisation: | FS PCC 9605 | Size (aa) | A domain binding pocket: | Proposed amino acid: |
| Fatty acid desaturase |  | FIS9605DRAFT_04771 | 320 |  |  |
| Hypothetical protein |  | FIS9605DRAFT_04770 | 253 |  |  |
| NRPS | A-PCP-C-A-PCP-TE | FIS9605DRAFT_04769 | 1980 | FS_A1: DPWATGCIDK  FS_A2: DTKDLGVVDK | Gln (60%)  Glu (90%) |
| Oxidoreductase |  | FIS9605DRAFT_04768 | 759 |  |  |

| Cluster 4: Orphan NRPS: | | | | | |
| --- | --- | --- | --- | --- | --- |
| Proposed function: | Domain organisation: | FS PCC 9605 | Size (aa) | A domain binding pocket: | Proposed amino acid: |
| NRPS | AS-ACP-C-A-PCP-E | FIS9605DRAFT_05903 | 2,228 | DVENVGAITK | Arg (70%) |
| NRPS | C-A-PCP-TE | FIS9605DRAFT_05904 | 1,328 | DATKIGEVGK | Asn (90%) |

| Cluster 5: Orphan NRPS/PKS: | | | | | |
| --- | --- | --- | --- | --- | --- |
| Proposed function: | Domain organisation: | FS PCC 9993: | Size (aa): | A domain binding pocket: | Proposed amino acid: |
| PKS/NRPS | AS-ACP-C-A-PCP | PCC9339DRAFT_05609 | 1,783 | DVFGVGAVFK | D-lyserg (70%) |
| PKS | KS-AT-MT-KR-ER-ACP | PCC9339DRAFT_05610 | 2,632 |  |  |
| PKS | KS-AT-KR-ACP | PCC9339DRAFT_05611 | 1,532 |  |  |
| NRPS | C-A-MT-PCP-TE | PCC9339DRAFT_05612 | 1,767 | DLFNNALTY- | Ala (90%) |

| Cluster 6: Orphan NRPS/PKS: | | | | | | | | | |
| --- | --- | --- | --- | --- | --- | --- | --- | --- | --- |
| Proposed function: | Domain organisation: | Subsection V cyanobacteria: | | | | | | A domain binding pocket: | Proposed amino acid: |
|  |  | WI HT-29-1 | | FS PCC 9431 | | HW IC-52-3 | |  |  |
|  |  | Locus Tag: | Size (aa) | Locus Tag: | Size (aa) | Locus Tag: | Size (aa) |  |  |
| PKS/NRPS | AS-ACP-C-A-PCP | HT291_02580 | 1,794 | Fis9431DRAFT_5546 | 1,794 | IC523_04614 | 1,794 | DMTKIGEVGK | Asn (90%) |
| Dioxygenase |  | HT291_02579 | 291 | Fis9431DRAFT_5545 | 291 | IC523_04615 | 291 |  |  |
| PKS | KS-AT-KR-ACP-E | HT291_02578 | 1,999 | Fis9431DRAFT_5544 | 1,999 | IC523_04616 | 1,999 |  |  |
| NRPS | C-A-PCP-C | HT291_02577 | 1,852 | Fis9431DRAFT_5543 | 1,852 | IC523_04617 | 1,852 | DILQLGLIWK | Gly (100%) |
| NRPS | C-A-PCP-C | HT291_02576 | 1,531 | Fis9431DRAFT_5542 | 1,529 | IC523_04618 | 1,529 | DLTKIGHVGK | Asn (90%) |
| Aminotransferase |  | HT291_02575 | 429 | Fis9431DRAFT_5541 | 431 | IC523_04619 | 431 |  |  |
| NRPS | A-PCP | HT291_02574 | 913 | Fis9431DRAFT_5540 | 931 | IC523_04620 | 913 | DIWEITADDK | Arg (60%) |
| NRPS | C-A-PCP-C | HT291_02573 | 1,602 | Fis9431DRAFT_5539 | 1,602 | IC523_04621 | 1,602 | DVWHISLIDK | Ser (100%) |
| Dioxygenase |  | HT291_02572 | 350 | Fis9431DRAFT_5538 | 350 | IC523_04622 | 226 |  |  |
| Transposase |  | - | - | - | - | IC523_04623  IC523_04624 | 320  156 |  |  |
| Dioxygenase |  | - | - | - | - | IC523_04625 | 105 |  |  |
| NRPS | C-A-PCP | HT291_02571 | 1,076 | Fis9431DRAFT_5537 | 1,076 | IC523_04626  (C-A-PCP-R) | 1,444 | DSALIAEVWK | His (70%) |
| NRPS | C-A-PCP-R | HT291_02570 | 1,504 | Fis9431DRAFT_5536 | 1,504 | - | - | DVQFIAHVVK | Pro (100%) |
| Methyltransferase |  | - |  | - |  | IC523_04627 | 249 |  |  |
| Aminotransferase |  | HT291_02569 | 484 | Fis9431DRAFT_5535 | 848 | IC523_04628 | 484 |  |  |

| Cluster 7: Orphan NRPS/PKS: | | | | | |
| --- | --- | --- | --- | --- | --- |
| Proposed function: | Domain organisation: | MR PCC 10914: | Size (aa): | A domain binding pocket: | Proposed amino acid: |
| PKS/NRPS | AS-ACP-C | Mas10914DRAFT_3583 | 1,183 |  |  |
| Dioxygenase |  | Mas10914DRAFT_3584 | 352 |  |  |
| NRPS | C-A-PCP-C-A-PCP-C | Mas10914DRAFT_3585 | 2,953 | A1: SSRDVAMIVK, A2: DILQLGLIWK | A1: Bht (40%), A2: Gly (100%) |
| NRPS | C-A-PCP-C-A-PCP | Mas10914DRAFT_3586 | 5,153 | A1: DLTKIGHVGH, A2: DVWHISLIDK | A1: Asn (90%), A2: Ser (100%) |
| PKS | KS-AT-KR-ACP | Mas10914DRAFT_3587 | 1,536 |  |  |
| NRPS | C-A-PCP-C | Mas10914DRAFT_3588 | 1,898 | DVWHIS LIDK | Ser (100%) |
| NRPS | C-A-PCP | Mas10914DRAFT_3589 | 1,054 | DLTKVGHVGK | Asp (100%) |
| NRPS | C-A-PCP-R | Mas10914DRAFT_3590 | 1,513 | DVGEIGSIDK | Phe |
| Aminotransferase |  | Mas10914DRAFT_3591 | 480 |  |  |

| Cluster 8: Orphan NRPS/PKS: | | | | | | | | | |
| --- | --- | --- | --- | --- | --- | --- | --- | --- | --- |
| Proposed function: | Domain organisation: | Subsection V cyanobacteria: | | | | | | A domain binding pocket: | Proposed amino acid: |
|  |  | CF PCC 6912 |  | CS PCC 9212 |  | MT BC008 |  |  |  |
|  |  | Locus Tag: | Size (aa) | Locus Tag: | Size (aa) | Locus Tag: | Size (aa) |  |  |
| PKS/NRPS | AS-ACP-C-A-PCP | UYCDRAFT_06525 | 1,778 | UYEDRAFT_04458 | 1,778 | YYIDRAFT_07421 | 1,793 | DVWHISLIDK | Ser (100%) |
| Glycosyltransferase |  | UYCDRAFT_06526 | 429 | UYEDRAFT_04459 | 429 | - |  |  |  |
| Dioxygenase |  | UYCDRAFT_06527 | 288 | UYEDRAFT_04460 | 288 | YYIDRAFT_07422 | 287 |  |  |
| PKS | KS-AT-KR-ACP-E | UYCDRAFT_06528 | 1,988 | UYEDRAFT_04461 | 1,988 | YYIDRAFT_07423 | 1,999 |  |  |
| NRPS | C-A-PCP-C | UYCDRAFT_06529 | 1,867 | UYEDRAFT_04462 | 1,867 | YYIDRAFT_07424  (C-A only)  End of scaffold | 752 | DILQLGLIWK | Gly (100%) |
| NRPS | C-A-PCP-C-A-PCP | UYCDRAFT_06530 | 2,132 | UYEDRAFT_04463 | 2,132 |  |  | A1:DLTKIGHVGH,  A2: DVGEIGSIDK | A1: Asn (90%), A2:Orn (100%) |
| NRPS | C-A-PCP | UYCDRAFT_06531 | 1,154 | UYEDRAFT_04464 | 1,154 |  |  | DVWHISLVDK | Ser (100%) |
| PKS | KS-AT-MT-KR-ACP | UYCDRAFT_06532 | 1,941 | UYEDRAFT_04465 | 1,941 |  |  |  |  |
| NRPS | C | UYCDRAFT_06533 | 523 | UYEDRAFT_04466 | 523 |  |  |  |  |
| Dioxygenase |  | UYCDRAFT_06534 | 341 | UYEDRAFT_04467 | 341 |  |  |  |  |
| NRPS | C-A-PCP | UYCDRAFT_06535 | 1,075 | UYEDRAFT_04468 | 1,075 |  |  | DLTKIGEVGK | Asn (100%) |
| NRPS | C-A-PCP-R | UYCDRAFT_06536 | 1,496 | UYEDRAFT_04469 | 1,496 |  |  | DVGEVGSIDK | Orn (100%) |
| Aminotransferase |  | UYCDRAFT_06537 | 474 | UYEDRAFT_04470 | 474 |  |  |  |  |
| Methyltransferase |  | UYCDRAFT_06538 | 249 | UYEDRAFT_04471 | 249 |  |  |  |  |

| Cluster 9: Orphan NRPS/PKS: | | | | | |
| --- | --- | --- | --- | --- | --- |
| Proposed function: | Domain organisation: | MT BC008: | Size (aa): | A domain binding pocket: | Proposed amino acid: |
| PKS | AS | YYIDRAFT_05354 | 569 |  |  |
| PKS | ACP | YYIDRAFT_05353 | 488 |  |  |
| PKS/NRPS | KS-AT-ACP-Aminotransferase-MT-C | YYIDRAFT_05352 | 2,499 |  |  |
| Hypothetical protein |  | YYIDRAFT_05351 | 197 |  |  |
| Dioxygenase |  | YYIDRAFT_05350 | 355 |  |  |
| NRPS | C-A-PCP-E-C | YYIDRAFT_05349 | 1,995 | DLTKIGHVGK | Asn (90%) |
| Aspartate racemase |  | YYIDRAFT_05348 | 246 |  |  |
| NRPS | A-PCP | YYIDRAFT_05347 | 635 | DAKHVALLTK | Hpg (60%) |
| NRPS | C-A-PCP-TE | YYIDRAFT_05346 | 1,420 | DASTIAAVCK | Tyr (100%) |
| Lysine 2,3-aminomutase |  | YYIDRAFT_05345 | 449 |  |  |
| Dioxygenase |  | YYIDRAFT_05344 | 342 |  |  |

| Cluster 10: Orphan PKS: | | | |
| --- | --- | --- | --- |
| Proposed function: | Domain organisation: | FS PCC 9339: | Size (aa): |
| PKS | AS | PCC9339DRAFT_05606 | 607 |
| Fatty acid desaturase |  | PCC9339DRAFT_05604 | 319 |
| Fatty acid desaturase |  | PCC9339DRAFT_05603 | 335 |
| PKS | ACP | PCC9339DRAFT_05602 | 114 |
| PKS | KS-AT-DH | PCC9339DRAFT_05601 | 1,036 |
| PKS | Monoxygenase-ACP-TE | PCC9339DRAFT_05600 | 994 |
| PKS | ACP | PCC9339DRAFT_05599 | 159 |
| Aminotransferase |  | PCC9339DRAFT_05598 | 462 |
| Fatty acid desaturase |  | PCC9339DRAFT_05597 | 376 |
| Transposase |  | PCC9339DRAFT_05596 | 213 |

| Cluster 11: Orphan PKS: | | | |
| --- | --- | --- | --- |
| Proposed function: | Domain organisation: | FS PCC 9339: | Size (aa): |
| Acyl-ACP synthetase | AS | PCC9339DRAFT_05219 | 588 |
| Fatty acid desaturase | - | PCC9339DRAFT_05218 | 337 |
| PKS | ACP | PCC9339DRAFT_05217 | 99 |
| Methyltransferase | - | PCC9339DRAFT_05216 | 299 |
| PKS | KS-AT-ACP | PCC9339DRAFT_05215 | 1,183 |
| Oxidase | - | PCC9339DRAFT_05214 | 448 |
| Hypothetical protein | - | PCC9339DRAFT_05213 | 157 |

| Cluster 12: Orphan PKS: | | | |
| --- | --- | --- | --- |
| Proposed function: | Domain organisation: | FS PCC 9605: | Size (aa): |
| PKS | AS | FIS9605DRAFT_06605 | 613 |
| PKS | ACP | FIS9605DRAFT_06604 | 105 |
| Hypothetical protein |  | FIS9605DRAFT_06603 | 453 |
| PKS | KS-AT-KR-ACP | FIS9605DRAFT_06602 | 1,607 |
| PKS | KS-AT-ACP | FIS9605DRAFT_06601 | 1,016 |
| Hypothetical protein |  | FIS9605DRAFT_06600 | 627 |
| Short-chain dehydrogenase |  | FIS9605DRAFT_06599 | 297 |

| Cluster 13: Orphan PKS: | | | |
| --- | --- | --- | --- |
| Proposed function: | Domain organisation: | MT BC008: | Size (aa): |
| Methyltransferase |  | YYIDRAFT_06980 | 319 |
| PKS | AS | YYIDRAFT_06979 | 627 |
| PKS | ACP | YYIDRAFT_06978 | 93 |
| Hypothetical protein |  | YYIDRAFT_06977 | 467 |
| PKS | KS-AT-KR-ACP-Red | YYIDRAFT_06976 | 1,989 |
| Hypothetical protein |  | YYIDRAFT_06975 | 768 |

| Cluster 14: Orphan NRPS: | | | | | |
| --- | --- | --- | --- | --- | --- |
| Proposed function: | Domain organisation: | FS PCC 9993: | Size (aa): | A domain binding pocket: | Proposed amino acid: |
| NRPS | C-A-PCP-E-C-A-PCP-E | PCC9339DRAFT_05503 | 3,137 | A1: DASTIAAVCK, A2: DLKNFGSDVK | A1: Tyr (100%) A2: Glu (50%) |
| NRPS | C-A-MT-PCP-C-A-PCP-  C-A-PCP-C-A-PCP-R | PCC9339DRAFT_05502 | 4,967 | A1: DVWHISLIDK, A2: DAFWLGGTFK, A3:DVQFISHLAK, A4: DASIIAVCK | A1: Ser (100%) A2: Val (90%)  A3: Pro (90%) A4: Tyr (90%) |

| Cluster 15: Orphan NRPS: | | | | | |
| --- | --- | --- | --- | --- | --- |
| Proposed function: | Domain organisation: | MR PCC 10914: | Size (aa): | A domain binding pocket: | Proposed amino acid: |
| NRPS | C-A-PCP-TE | Mas10914DRAFT_3966 | 1,395 | SSRDVTMGTK | Thr (30%) |

| Cluster 16: Orphan NRPS/PKS: | | | | | |
| --- | --- | --- | --- | --- | --- |
| Proposed function: | Domain organisation: | MR PCC 10914: | Size (aa): | A domain binding pocket: | Proposed amino acid: |
| NRPS | C-A-PCP | Mas10914DRAFT_3745 | 1,097 | DAFFLGVTFK | Ile (100%) |
| Hypothetical protein |  | Mas10914DRAFT_3746 | 93 |  |  |
| NRPS | C-A-MT-PCP | Mas10914DRAFT_3747 | 1,203 | DAWFLGNVVK | Leu (100%) |
| Hypothetical protein |  | Mas10914DRAFT_3748 | 67 |  |  |
| NRPS | C-A-PCP-TE | Mas10914DRAFT_3749 | 1,371 | DAFTIAAVWK | Phe (80%) |

| Cluster 17: Orphan NRPS/PKS: | | | | | | | | | |
| --- | --- | --- | --- | --- | --- | --- | --- | --- | --- |
| Proposed function: | Domain organisation: | Subsection V cyanobacteria: | | | | | | A domain binding pocket: | Proposed amino acid: |
|  |  | HW IC-52-3 | | FS PCC 9431 | | FM SAG 1427-1 | |  |  |
|  |  | Locus Tag: | Size (aa) | Locus Tag: | Size (aa) | Locus Tag: | Size (aa) |  |  |
| Hypothetical protein |  | IC523_04490 | 264 | Fis9431DRAFT_5429 | 264 |  |  |  |  |
| 2-oxoglutarate and iron-dependent oxgenase |  | IC523_04489 | 268 | Fis9431DRAFT_5428 | 268 |  |  |  |  |
| NRPS | C-A-PCP-C | IC523_04488 | 1,548 | Fis9431DRAFT_5427 | 1,548 | - |  | DATKMGHVGK | Asp (90%) |
| Asparagine synthase |  | IC523_04487 | 626 | Fis9431DRAFT_5426 | 626 | - |  |  |  |
| Asparagine racemase |  | IC523_04486 | 287 | Fis9431DRAFT_5425 | 287 | - |  |  |  |
| NRPS | A-PCP | IC523_04485 | 628 | Fis9431DRAFT_5424 | 628 | - |  | DARHLSLMTK | Hpg (60%) |
| NRPS | C-A-PCP-TE | IC523_04484 | 1420 | Fis9431DRAFT_5423 | 1420 | UYGDRAFT_05852  (C-A-PCP) | 1,122 | DASTIAAVCK | Tyr (100%) |

Incomplete NRPS/PKS:

| Cluster 18: Orphan NRPS/PKS: | | | | |
| --- | --- | --- | --- | --- |
| Proposed function: | Domain organisation: | FS PCC 9339: | A domain binding pocket: | Proposed amino acid: |
| NRPS/PKS | A-KR-PCP-KS-AT-KR-ACP | PCC9339DRAFT_05404 | AILWVAASG- | Tcl (60%) |
| NRPS | C-A-PCP-C | PCC9339DRAFT_05405 | DILQLGLIWK | Gly |
| NRPS (N’s in seq) | A-PCP-C-A-PCP-C-A-PCP-TE | PCC9339DRAFT_05406 | A1: DAWTIAAICK  A2: DVQFIAYVAK  A3: DAWQFGIIDK | A1: Phe  A2: Pro (80%)  A3: Gln (90%) |
| B-lactamase |  | PCC9339DRAFT_05407 |  |  |
| Methyltansferase |  | PCC9339DRAFT_05408 |  |  |

| Cluster 19: Orphan NRPS/PKS: | | | | | |
| --- | --- | --- | --- | --- | --- |
| Proposed function: | Domain organisation: | FS PCC 9339: | Size (aa): | A domain binding pocket: | Proposed amino acid: |
| PKS | AS-ACP | PCC9339DRAFT_05703 | 767 |  |  |
| PKS | KS | PCC9339DRAFT_05704 | 374 |  |  |
| PKS | DH-MT-ER-KR-ACP | PCC9339DRAFT_05705 | 1,726 |  |  |
| PKS | KS-AT-MT-KR-ACP | PCC9339DRAFT_05706 | 1,917 |  |  |
| NRPS | Cy-A-PCP | PCC9339DRAFT_05707 | 1,420 | DLYNLSLIWK | Cys (100%) |
| PKS | KS-AT-MT-ACP-TE | PCC9339DRAFT_05708 | 1,716 |  |  |

| Cluster 20: Orphan NRPS/PKS: | | | | |
| --- | --- | --- | --- | --- |
| Proposed function: | Domain organisation: | FS PCC 9339: | A domain binding pocket: | Proposed amino acid: |
| NRPS | A-PCP | PCC9339DRAFT_06041 | DVQFIAQAVK | Pro (80%) |
| NRPS | C | PCC9339DRAFT_06042 |  |  |

| Cluster 21: Orphan NRPS/PKS: | | | | |
| --- | --- | --- | --- | --- |
| Proposed function: | Domain organisation: | MR PCC 10914: | A domain binding pocket: | Proposed amino acid: |
| NRPS | C-UNK-PCP | Mas10914DRAFT_4942 |  |  |
| NRPS | Cy-A | Mas10914DRAFT_4943 | DLYNLSLIW- | Cys (90%) |
| PKS | AS | Mas10914DRAFT_4944 |  |  |

| Cluster 22: Orphan NRPS/PKS: | | |
| --- | --- | --- |
| Proposed function: | Domain organisation: | FS PCC 9339: |
| PKS | KS | PCC9339DRAFT_05437 |
| PKS | ACP-KR-DH-UNK-UNK | PCC9339DRAFT_05438 |
| Tryptophan halogenase |  | PCC9339DRAFT_05439 |
| Asparagine synthase |  | PCC9339DRAFT_05440 |

| Cluster 23: Orphan PKS: | | |
| --- | --- | --- |
| Proposed function: | Domain organisation: | FS PCC 9339: |
| PKS | UNK-ACP-KS-AT-ACP | PCC9339DRAFT_06064 |
| PKS | KS-AT-KR-ACP | PCC9339DRAFT_06063 |
| PKS | KS-AT-DH-ACP | PCC9339DRAFT_06062 |
| PKS | Reduction | PCC9339DRAFT_06061 |

| Cluster 24: Orphan NRPS/PKS: | | |
| --- | --- | --- |
| Proposed function: | Domain organisation: | MT BC008: |
| PfaD |  | YYIDRAFT_00748 |
| PKS | KS-AT-ACP-ACP | YYIDRAFT_00749 |
| Dehydrogenase |  | YYIDRAFT_00750 |
| PKS | KS-KS-AT-UNK-UNK | YYIDRAFT_00751 |

| Cluster 25: Orphan NRPS/PKS: | | | | |
| --- | --- | --- | --- | --- |
| Proposed function: | Domain organisation: | FM SAG 1427-1: | A domain binding pocket: | Proposed amino acid: |
| PKS | KS-AT-KR-ACP | UYGDRAFT_04103 |  |  |
| NRPS | C-A | UYGDRAFT_04102 End of scaffold | DILQLGMIW- | Gly 90% |

| Cluster 26: Orphan NRPS/PKS: | | | | |
| --- | --- | --- | --- | --- |
| Proposed function: | Domain organisation: | MT BC008: | A domain binding pocket: | Proposed amino acid: |
| PKS/NRPS | AS-ACP-Monooxygenase-C-A | YYIDRAFT_10452  End of scaffold | DAWFLGNVVK | Leu 100% |

| Cluster 27: Orphan NRPS/PKS: | | | | |
| --- | --- | --- | --- | --- |
| Proposed function: | Domain organisation: | MT BC008: | A domain binding pocket: | Proposed amino acid: |
| NRPS | A-PCP-C | Beginning of scaffold  YYIDRAFT_11887 | DILQLGMIWK | Gly 100% |
| NRPS | C-A-PCP-C | YYIDRAFT_11886  End of scaffold | DLTKIGHIGK | Asp 90% |

| Cluster 28: Orphan NRPS/PKS: | | | | |
| --- | --- | --- | --- | --- |
| Proposed function: | Domain organisation: | MT BC008: | A domain binding pocket: | Proposed amino acid: |
| NRPS | A-PCP | Beginning of scaffold  YYIDRAFT_12315 | DVQFIAQVVK | Pro 90% |
| NRPS | C-A-PCP-Red | YYIDRAFT_12314  End of scaffold | DVENVGAITK | Arg 70% |

| Cluster 29: Orphan NRPS/PKS: | | | | |
| --- | --- | --- | --- | --- |
| Proposed function: | Domain organisation: | MT BC008: | A domain binding pocket: | Proposed amino acid: |
| NRPS | PCP-C-A-PCP-TE | Beginning of scaffold YYIDRAFT_12322 | DAWFLGNVVK | Leu 100% |

| Cluster 30: Orphan NRPS/PKS: | | | | |
| --- | --- | --- | --- | --- |
| Proposed function: | Domain organisation: | MT BC008: | A domain binding pocket: | Proposed amino acid: |
| NRPS | A-KR-PCP | Only gene on scaffold  YYIDRAFT_12606 | GLFWLGASG- | Alpha-hydroxy-isocaproic acid 70% |

| Cluster 31: Orphan NRPS/PKS: | | |
| --- | --- | --- |
| Proposed function: | Domain organisation: | MT BC008: |
| PKS | ACP | Beginning of scaffold YYIDRAFT_12646 |
| PKS | KS-AT-KR-PCP | YYIDRAFT_12647 |
| NRPS | C | YYIDRAFT_12648  End of scaffold |

| Cluster 32: Orphan NRPS/PKS: | | | | | |
| --- | --- | --- | --- | --- | --- |
| Proposed function: | Domain organisation: | Subsection V cyanobacteria: | | A domain binding pocket: | Proposed amino acid: |
|  |  | CS PCC 9212 | CF PCC 6912 |  |  |
| PKS | ACP-KS-AT | Start of scaffold UYEDRAFT_05051 | Start of scaffold  UYCDRAFT_01667 |  |  |
| NRPS | ACP-C-A-PCP-Red | UYEDRAFT_05052 | UYCDRAFT_01666 | DLFNNALTYK | Ala (100%) |
| Hypothetical protein |  | UYEDRAFT_05053 | UYCDRAFT_01665 |  |  |
| PfaD family protein |  | UYEDRAFT_05054 | UYCDRAFT_01664 |  |  |

| Cluster 33: Orphan NRPS/PKS: | | | | |
| --- | --- | --- | --- | --- |
| Proposed function: | Domain organisation: | MT BC008: | A domain binding pocket: | Proposed amino acid: |
| HAD superfamily phosphatase |  | YYIDRAFT_11144 |  |  |
| NRPS | C-A-PCP-E | YYIDRAFT_11143 | DVEDIGAVEK | Arg (80%) |
| NRPS | C | YYIDRAFT_11142  End of scaffold |  |  |

| Cluster 34: Orphan NRPS/PKS: | | | | |
| --- | --- | --- | --- | --- |
| Proposed function: | Domain organisation: | FS PCC 9605: | A domain binding pocket: | Proposed amino acid: |
| NRPS | A-PCP-C-A-PCP | Beginning of scaffold FIS9605DRAFT_05481 | A1: DLFNNALTYK  A2: DILQLGMIWK | A1: Ala (100%)  A2: Gly (100%) |
| PKS | KS-AT-KR-PCP-E | FIS9605DRAFT_05480 |  |  |
| NRPS | C | FIS9605DRAFT_05479 |  |  |
| Cupin superfamily protein | - | FIS9605DRAFT_05478 |  |  |
| Aminotransferase | AMT | FIS9605DRAFT_05477 |  |  |
| NRPS | A-PCP | FIS9605DRAFT_05476 | DIWEMVADDK | Ser (50%) |
| NRPS | PCP | FIS9605DRAFT_05475 |  |  |
| Glutamate decarboxylase | AMT | FIS9605DRAFT_05474 |  |  |
| PKS | KS-AT | FIS9605DRAFT_05473 |  |  |
| Glutamate decarboxylase | - | FIS9605DRAFT_05472 |  |  |
| PKS | KS-AT-KR-ACP | FIS9605DRAFT_05471 |  |  |
| Methyltransferase | - | FIS9605DRAFT_05470 |  |  |
| NRPS | C-A-PCP-TE | FIS9605DRAFT_05469 | VDWIISLANK | Ala-b (80%) |
| Cytochrome p450 | - | FIS9605DRAFT_05468 |  |  |
| Short chain dehydrogenase | - | FIS9605DRAFT_05467 |  |  |

| Cluster 35: Orphan NRPS/PKS: | | | | | | |
| --- | --- | --- | --- | --- | --- | --- |
| Proposed function: | Domain organisation: | Subsection V cyanobacteria: | | | A domain binding pocket: | Proposed amino acid: |
|  |  | FM PCC 7414 | FS JSC-11 | FT PCC 7521 |  |  |
| PKS/NRPS | AS-ACP-C-A-PCP | UYIDRAFT_02316 | FJSC11DRAFT_1703 | UYKDRAFT_03968 | DVWHISLIDK | Ser (100%) |
| PKS/NRPS | KS-AT-KR-PCP | UYIDRAFT_02317 | FJSC11DRAFT_1702 | UYKDRAFT_03967 |  |  |
| Monooxygenase |  | UYIDRAFT_02318 | - | - |  |  |
| Hypothetical protein |  | - | FJSC11DRAFT_1701 | UYKDRAFT_03966 |  |  |
| NRPS | C-A-PCP-E | UYIDRAFT_02319 | FJSC11DRAFT_1700 | UYKDRAFT_03965 | DAEDIGTVVK | Glu (70%) |
| NRPS | C-A-PCP-C-A-PCP | UYIDRAFT_02320 | FJSC11DRAFT_1699 | UYKDRAFT_03964  (C-A-PCP-C-PCP)  (End of scaffold)  (Beginning of scaffold)  UYKDRAFT_03963  (A-PCP) | A1:  DVGEIGSIDK  A2: VDWVVSLADK | A1: Orn (100%)  A2: Ala-b (90%) |
| PKS/NPRS | KS-AT-KR-PCP | UYIDRAFT_02321 | FJSC11DRAFT_1698 | UYKDRAFT_03962 |  |  |
| Hypothetical protein |  | UYIDRAFT_02322 | FJSC11DRAFT_1697 | UYKDRAFT_03961 |  |  |
| Monooxygenase |  | UYIDRAFT_02323 | FJSC11DRAFT_1696 | UYKDRAFT_03960 |  |  |
| NRPS | C-A-PCP | UYIDRAFT_02324 | FJSC11DRAFT_1695  C (partial)  (End of scaffold)  (Beginning of scaffold)  FJSC11DRAFT_4608 (A-PCP) | UYKDRAFT_03959 (C) (End of scaffold)  (Beginning of scaffold) UYKDRAFT_03958  (A-PCP) | DILQLGLIWK | Gly (100%) |
| Decarboxylase |  | UYIDRAFT_02325 | FJSC11DRAFT_4609 | UYKDRAFT_03957 |  | - |
| PKS | KS-AT-KR-ACP | UYIDRAFT_02326 (KS) (End of scaffold) | FJSC11DRAFT_4610 | UYKDRAFT_03956 (KS) (End of scaffold) |  | - |
| PKS | KS | - | FJSC11DRAFT_4611  (End of scaffold) | - |  | - |

| Cluster 36: Orphan NRPS/PKS: | | | | |
| --- | --- | --- | --- | --- |
| Proposed function: | Domain organisation: | FS PCC 9339: | A domain binding pocket: | Proposed amino acid: |
| PKS | AS-ACP-KS-AT-KR-ACP | PCC9339DRAFT_05235 |  |  |
| Hypothetical protein |  | PCC9339DRAFT_05236 |  |  |
| Hypothetical protein |  | PCC9339DRAFT_05237 |  |  |
| Transposase |  | PCC9339DRAFT_05238 |  |  |
| Transposase |  | PCC9339DRAFT_05239 |  |  |
| Transposase |  | PCC9339DRAFT_05240 |  |  |
| PKS | KS-AT-DH-KR-ACP | PCC9339DRAFT_05241 |  |  |
| NRPS | C-A-PCP | PCC9339DRAFT_05242 | DIWELTADDK | Ser (50%) |
| Transposase |  | PCC9339DRAFT_05243 |  |  |
| Hypothetical protein |  | PCC9339DRAFT_05244 |  |  |
| Transposase |  | PCC9339DRAFT_05245 |  |  |
| Transposase |  | PCC9339DRAFT_05246 |  |  |
| Hypothetical protein |  | PCC9339DRAFT_05247 |  |  |
| NRPS | C-A-PCP-C-A-PCP | PCC9339DRAFT_05248 | A1: DATKVGEVGK  A2: DAFWLGGTFK | A1: Asn (100%)  A2: Val (90%) |
| N’s in sequence |  |  |  |  |
| NRPS | PCP-C | PCC9339DRAFT_05249 |  |  |
| Transposase |  | PCC9339DRAFT_05250 |  |  |
| Racemase |  | PCC9339DRAFT_05251 |  |  |
| NRPS | A-PCP-C-A-PCP-TE | PCC9339DRAFT_05252 | A1: DARHLSLMVK  A2: DILQLGLIWK | A1: Hpg (60%)  A2: Gly (100%) |
| N’s in sequence | | | | |
| NRPS | PCP-TE | PCC9339DRAFT_05253 |  |  |
| Hypothetical protein |  | PCC9339DRAFT_05254 |  |  |

| Cluster 37: Orphan PKS: | | | |
| --- | --- | --- | --- |
| Proposed function: | Domain organisation: | Subsection V cyanobacteria: | |
|  |  | CS PCC 9212 | CF PCC 6912 |
| PKS | KS | UYEDRAFT_00573 | UYCDRAFT_02913 |
| Hypothetical protein |  | UYEDRAFT_00574 | UYCDRAFT_02912 |
| PKS | KR-DH | UYEDRAFT_00575 | UYCDRAFT_02911 |
| PKS | KS-AT | UYEDRAFT_00576 | UYCDRAFT_02910 |
| Dioxygenase |  | UYEDRAFT_00577 | UYCDRAFT_02909 |
| ACP |  | UYEDRAFT_00578 | UYCDRAFT_02908 |
| PKS | KS-AT-ACP-TE | UYEDRAFT_00579 | UYCDRAFT_02907 |

| Cluster 38: Orphan NRPS/PKS: | | | | |
| --- | --- | --- | --- | --- |
| Proposed function: | Domain organisation: | FS PCC 9431: | A domain binding pocket: | Proposed amino acid: |
| PKS | AS | Fis9431DRAFT_5794 |  |  |
| Hypothetical protein |  | Fis9431DRAFT_5795 |  |  |
| Hypothetical protein |  | Fis9431DRAFT_5796 |  |  |
| Fatty acid desaturase |  | Fis9431DRAFT_5797 |  |  |
| PKS | ACP | Fis9431DRAFT_5798 |  |  |
| Cyclopropane fatty acid synthase and related methyltransferases |  | Fis9431DRAFT_5799 |  |  |
| PKS | KS-AT | Fis9431DRAFT_5800 |  |  |
| PKS | KS-AT-ACP | Fis9431DRAFT_5801 |  |  |
| NRPS | Cy-A-PCP | Fis9431DRAFT_5802 | DLYNFSLIWK | Cys (90%) |
| NRPS | C-UNK-A-MT-PCP-TE | Fis9431DRAFT_5803 | D--QMGMVWK | Leu (60%) |
| Cupin superfamily protein |  | Fis9431DRAFT_5804 |  |  |
| Cytochrome p450 |  | Fis9431DRAFT_5805 |  |  |
| PKS | ACP | Fis9431DRAFT_5806 |  |  |
| Hypothetical protein |  | Fis9431DRAFT_5807 |  |  |

| Cluster 39: Orphan NRPS/PKS: | | | | |
| --- | --- | --- | --- | --- |
| Proposed function: | Domain organisation: | FS PCC 9339 | A domain binding pocket: | Proposed amino acid: |
| PKS/NRPS | AS-ACP-C-A-PCP | PCC9339DRAFT_06331 | DLFNNALTYK | Ala (100%) |
| Carbamoyl transferase | - | PCC9339DRAFT_06332 |  |  |
| PKS | AT | PCC9339DRAFT_06333 |  |  |
| 3-hydroxyacyl-CoA dehydrogenase | - | PCC9339DRAFT_06334 |  |  |
| PKS | ACP | PCC9339DRAFT_06335 |  |  |
| Cupin superfamily protein | - | PCC9339DRAFT_06336 |  |  |
| Cupin superfamily protein | - | PCC9339DRAFT_06337 |  |  |
| Acyl-CoA dehydrogenase | - | PCC9339DRAFT_06338 |  |  |
| Cupin superfamily protein | - | PCC9339DRAFT_06339 |  |  |
| HAD-superfamily phosphatase | - | PCC9339DRAFT_06340 |  |  |
| PKS/NRPS | KS-AT-KR-ACP-C | PCC9339DRAFT_06341 |  |  |
| NRPS | A-PCP | PCC9339DRAFT_06342 | DIWEMVADDK | Ser (50%) |
| PKS | KS-AT-KR-ACP | PCC9339DRAFT_06343 |  |  |
| PKS/NRPS | KS-AT-KR-ACP-E | PCC9339DRAFT_06344 |  |  |
| NRPS | C | PCC9339DRAFT_06345 |  |  |
| Cupin superfamily protein (JmjC domain) | - | PCC9339DRAFT_06346 |  |  |
| Aminotransferase | - | PCC9339DRAFT_06347 |  |  |
| NRPS | A-PCP | PCC9339DRAFT_06348 | DIWEMVADDK | Ser (50%) |
| NRPS | C-A-PCP-C-A-PCP-C | PCC9339DRAFT_06349 | A1: DVWHISLVDK,  A2: DILQLGMIWK | A1: Ser (100%)  A2: Gly (100%) |
| Dioxygenase | - | PCC9339DRAFT_06350 |  |  |
| NRPS | C-A-PCP-R | PCC9339DRAFT_06351 | DLTKVGHVGK | Asp (100%) |
| Oxidoreductase | - | PCC9339DRAFT_06352 |  |  |

| Cluster 40: Orphan NRPS/PKS: | | | | | | |
| --- | --- | --- | --- | --- | --- | --- |
| Proposed function: | Domain organisation: | Subsection V cyanobacteria: | | | A domain binding pocket: | Proposed amino acid: |
|  |  | FS PCC 9339 | FM SAG 1427-1 | FS PCC 9605 |  |  |
| NRPS | AS-ACP-C-A-PCP-E | PCC9339DRAFT_05572 | - | Beginning of scaffold FIS9605DRAFT_05481 (A-PCP) | DLFNNALTYK | Ala (100%) |
| NRPS | C | PCC9339DRAFT_05573 | - | - |  |  |
| NRPS | PCP-C-A-PCP-C | PCC9339DRAFT_05574 | - | FIS9605DRAFT_05481 (C-A-PCP) | DILQLGMIWK | Gly (100%) |
| Dioxygenase |  | PCC9339DRAFT_05575 | - | - |  |  |
| NRPS | A-PCP-C-A-PCP | PCC9339DRAFT_05576 | - | - | DLTKIGEVGK | Asn (100%) |
| PKS | KS-AT-KR-PCP-E | PCC9339DRAFT_05577 | UYGDRAFT_04100 | FIS9605DRAFT_05480 |  |  |
| NRPS | C | PCC9339DRAFT_05578 | UYGDRAFT_04099 | FIS9605DRAFT_05479 |  |  |
| Cupin superfamily protein |  | PCC9339DRAFT_05579 | UYGDRAFT_04098 | FIS9605DRAFT_05478 |  |  |
| Aminotransferase |  | PCC9339DRAFT_05580 | UYGDRAFT_04097 | FIS9605DRAFT_05477 |  |  |
| NRPS | A-PCP | PCC9339DRAFT_05581 | UYGDRAFT_04096 | FIS9605DRAFT_05476 | DIWEMVADDK | Ser (50%) |
| NRPS | PCP | - | - | FIS9605DRAFT_05475 |  |  |
| Glutamate decarboxylase |  | - | - | FIS9605DRAFT_05474 |  |  |
| PKS | KS-AT-KR-ACP | PCC9339DRAFT_05582 | UYGDRAFT_04095 | FIS9605DRAFT_05473 (KS-AT only) |  |  |
| Aspartate decarboxylase |  | PCC9339DRAFT_05583 | UYGDRAFT_04094 | FIS9605DRAFT_05472 |  |  |
| PKS | KS-AT-KR-ACP | PCC9339DRAFT_05584 | - | - |  |  |
| PKS | KS-AT-KR-ACP | PCC9339DRAFT_05585 | UYGDRAFT_04093 | FIS9605DRAFT_05471 |  |  |
| Methyltransferase |  | PCC9339DRAFT_05586 | UYGDRAFT_04092  UYGDRAFT_04091 | FIS9605DRAFT_05470 |  |  |
| NRPS | C-A-PCP-TE | PCC9339DRAFT_05587 | UYGDRAFT_04090 | FIS9605DRAFT_05469 | VDWIISLANK | Ala-b (80%) |
| Cytochrome p450 |  | PCC9339DRAFT_05588  PCC9339DRAFT_05589 | UYGDRAFT_04089  UYGDRAFT_04088 | FIS9605DRAFT_05468 |  |  |
| Short chain dehydrogenase |  | PCC9339DRAFT_05590 | UYGDRAFT_04087 | FIS9605DRAFT_05467 |  |  |
| Na+=driven multidrug efflux pump |  | PCC9339DRAFT_05591 | UYGDRAFT_04086 |  |  |  |
| Hypothetical protein |  | PCC9339DRAFT_05592 |  |  |  |  |
| Short chain dehydrogenase |  | PCC9339DRAFT_05593 |  |  |  |  |

| Cluster 41: Orphan NRPS/PKS: | | | | | |
| --- | --- | --- | --- | --- | --- |
| Proposed function: | Domain organisation: | FS PCC 9339: | Size (aa): | A domain binding pocket: | Proposed amino acid: |
| PKS/NRPS | AS-ACP-C-A-PCP-UNK-A | PCC9339DRAFT_05309 | 2,793 | A1: DFWNIGMVHK  A2: DSALIAEVWK | Thr (100%)  His (70%) |
| NRPS | PCP | PCC9339DRAFT_05310 | 147 |  |  |
| NRPS | C | PCC9339DRAFT_05311 | 475 |  |  |
| Hypothetical protein |  | PCC9339DRAFT_05312 | 42 |  |  |
| Asparagine synthase |  | PCC9339DRAFT_05313 | 611 |  |  |
| NRPS | UNK-A-PCP-C-A-PCP | PCC9339DRAFT_05314 | 2,133 | A1: DVWHFSLIEK  A2: DILQLGLIWK | Ser (100%)  Gly (100%) |
| PKS | KS | PCC9339DRAFT_05315 | 459 |  |  |
| PKS/NRPS | AT-KR-ACP-Cy-A-PCP | PCC9339DRAFT_05316 | 2,429 | DLYNLSLIWK | Cys (100%) |
| Hypothetical protein |  | PCC9339DRAFT_05317 | 108 |  |  |
| NRPS | C-A-PCP-TE | PCC9339DRAFT_05318 | 1,409 | DVENIGAIGK | Orn (60%) |

| Cluster 42: Orphan PKS: | | | | |
| --- | --- | --- | --- | --- |
| Proposed function: | Domain organisation: | Subsection V cyanobacteria: | | |
|  |  | FT PCC 7521 | WI HT-29-1 | FM SAG 1427-1 |
| PKS | KS-AT-ACP-ACP-KR-DH | UYKDRAFT_00878 | HT291_03862 | UYGDRAFT_01973 |
| Dehydrogenase (FAD-binding) |  | UYKDRAFT_00879 UYKDRAFT_00880 | HT291_03863 | UYGDRAFT_01972  End of scaffold |
| NAD-dependent aldehyde dehydrogenase |  | UYKDRAFT_00881 | HT291_03864 |  |
| Predicted metal-dependent hydrolase |  | - | HT291_03865 |  |
| Dioxygenase |  | UYKDRAFT_00882 | HT291_03866 |  |
| Mo-dependent nitrogenase C-terminus |  | UYKDRAFT_00883 | - |  |
| Domain of unknown function (DUF1702) |  | UYKDRAFT_00884  UYKDRAFT_00885 | HT291_03867  HT291_03868 |  |
| Dehydrogenase |  | UYKDRAFT_00886 | HT291_03869 |  |

| Cluster 43: Orphan PKS: | | | | |
| --- | --- | --- | --- | --- |
| Proposed function: | Domain organisation: | Subsection V cyanobacteria: | | |
|  |  | FS PCC 9339 | WI HT-29-1 | FM SAG 1427-1 |
| PKS | KS-AT-ACP-KR-DH | PCC9339DRAFT_06207 | HT291_05362 | UYGDRAFT_06099 |
| Hypothetical protein |  | PCC9339DRAFT_06206 | HT291_05363 | UYGDRAFT_06098 |
| Dehydrogenase |  | PCC9339DRAFT_06205 | HT291_05364 | UYGDRAFT_06097 |
| Tryptophan halogenase |  | PCC9339DRAFT_06204 | HT291_05365 | UYGDRAFT_06096 |
| Hypothetical protein |  | PCC9339DRAFT_06203 | HT291_05366 | UYGDRAFT_06095 |
| Domain of unknown function (DUF3291) |  | PCC9339DRAFT_06202 | HT291_05367 | UYGDRAFT_06094 |
| Domain of unknown function (DUF2855) |  | PCC9339DRAFT_06201 | HT291_05368 | UYGDRAFT_06093 |

| Cluster 44: Orphan NRPS/PKS: | | | | | | |
| --- | --- | --- | --- | --- | --- | --- |
| Proposed function: | Domain organisation: | Subsection V cyanobacteria: | | | A domain binding pocket: | Proposed amino acid: |
|  |  | HW IC-52-3 | FS PCC 9339 | MT BC008 |  |  |
| PKS | AS | IC523_02336 | PCC9339DRAFT_00105 |  |  |  |
| Transmembrane secretion effector |  | IC523_02337 | PCC9339DRAFT_00106 |  |  |  |
| Probable taurine catabolism dioxygenase |  | IC523_02338 | PCC9339DRAFT_00100 |  |  |  |
| Domain of unknown function (DUF2662) |  | IC523_02339 | PCC9339DRAFT_00101 |  |  |  |
| PKS | ACP | IC523_02340 | PCC9339DRAFT_00102 | YYIDRAFT_11764 |  |  |
| NRPS | C-A-PCP-Cy-A-PCP | IC523_02341 | PCC9339DRAFT_00103 | YYIDRAFT_11765  (C-A-PCP-Cy-A only)  End of scaffold | A1: DVGEVGSIDK  A2: DLYNLSLIWK | A1: Orn (100%)  A2: Cys (100%) |
| NRPS | Cy-A-PCP-TE | IC523_02342 | PCC9339DRAFT_00104 (Cy-A-PCP) |  | DLYNLSLIWK | Cys (100%) |

| Cluster 45: Orphan NRPS/PKS: | | | | | |
| --- | --- | --- | --- | --- | --- |
| Proposed function: | Domain organisation: | Subsection V cyanobacteria: | | A domain binding pocket: | Proposed amino acid: |
|  |  | FS PCC 9605 | FM SAG 1427-1 |  |  |
| PKS/NRPS | AS-ACP-C-A-PCP | FIS9605DRAFT_05970 | UYGDRAFT_04113 | DILQLGLIWK | Gly (100%) |
| PKS | AT | FIS9605DRAFT_05969 | - |  |  |
| 3-hydroxyacyl-CoA dehydrogenase |  | FIS9605DRAFT_05968 | UYGDRAFT_04112 |  |  |
| PKS | ACP | FIS9605DRAFT_05967 | UYGDRAFT_04111 |  |  |
| Acyl-CoA dehydrogenase |  | FIS9605DRAFT_05966 | UYGDRAFT_04110 |  |  |
| Cupin superfamily protein |  | FIS9605DRAFT_05965 | - |  |  |
| HAD-superfamily phosphatase |  | FIS9605DRAFT_05964 | UYGDRAFT_04109 |  |  |
| PKS | KS-UNK-KR-ACP-E | FIS9605DRAFT_05963 | - |  |  |
| NRPS | C | FIS9605DRAFT_05962 | UYGDRAFT_04108  UYGDRAFT_04107  End of scaffold |  |  |
| NRPS | A-PCP-C | FIS9605DRAFT_05961 |  | DIWEMVADDK | Ser (50%) |

| Cluster 46: Orphan NRPS/PKS: | | | | |
| --- | --- | --- | --- | --- |
| Proposed function: | Domain organisation: | Subsection V cyanobacteria: | | |
|  |  | FS PCC 9431 | WI HT-29-1 | HW IC-52-3 |
| PKS | UNK-KS-AT | Fis9431DRAFT_1073 | HT291_00486 | IC523_04221 |
| PKS | KR | Fis9431DRAFT_1072 | HT291_00485 | IC523_04222 |
| Hypothetical protein |  | - | - | IC523_04223 |
| 4-amino-4-deoxy-L-arabinose transferase and related glycosyltransferases of PMT family |  | Fis9431DRAFT_1071 | HT291_00484 | IC523_04224 |
| Cyclopropane fatty acid synthase and related methyltransferases |  | Fis9431DRAFT_1070 | HT291_00483 | IC523_04225 |
| Uncharacterized protein, putative amidase |  | Fis9431DRAFT_1069 | HT291_00482 | IC523_04226 |
| Mo-dependent nitrogenase C-terminus |  | Fis9431DRAFT_1068 | HT291_00481 | IC523_04227 |

| Cluster 47: Orphan NRPS/PKS: | | | | | | | |
| --- | --- | --- | --- | --- | --- | --- | --- |
| Proposed function: | Domain organisation: | Subsection V cyanobacteria: | | | | A domain binding pocket: | Proposed amino acid: |
|  |  | MT BC008 | FT PCC 7521 | FS JSC11 | FM PCC 7414 |  |  |
| NRPS | A-PCP-C-A-E-PCP | Beginning of scaffold  YYIDRAFT_06440 | - | - |  | A1: DILQLGMIWK  A2: VDAVFSLADK | Gly (100%)  Beta-ala (100%) |
| PKS | KS-AT-KR-ACP | YYIDRAFT_06441 | Beginning of scaffold UYKDRAFT_03951  (KR-ACP) | Beginning of scaffold FJSC11DRAFT_0494  (KR-ACP) | Beginning of scaffold UYIDRAFT_02331  (KR-ACP) |  |  |
| Methyltransferase |  | - | UYKDRAFT_03950 | FJSC11DRAFT_0493 | UYIDRAFT_02332 |  |  |
| NRPS | C-A-PCP-TE | YYIDRAFT_06442 | UYKDRAFT_03949 | FJSC11DRAFT_0492 | UYIDRAFT_02333 | VDWVISLANK | Ala-b (90%) |
| Monooxygenase/ Cytochrome p450 |  | YYIDRAFT_06443  YYIDRAFT_06444 | UYKDRAFT_03948 | FJSC11DRAFT_0491 | UYIDRAFT_02334 |  |  |
| Short-chain dehydrogenase |  | - | UYKDRAFT_03947 | FJSC11DRAFT_0490 | UYIDRAFT_02335 |  |  |
| Hypothetical protein |  | YYIDRAFT_06445 | UYKDRAFT_03946 | FJSC11DRAFT_0489 | UYIDRAFT_02336 |  |  |
| Multi antimicrobial extrusion protein MatE/ Na+-driven multidrug efflux pump |  | YYIDRAFT_06446 | UYKDRAFT_03945 | FJSC11DRAFT_0488 | UYIDRAFT_02337 |  |  |
| MbtH domain protein |  | YYIDRAFT_06448 | UYKDRAFT_03944  UYKDRAFT_03943 | FJSC11DRAFT_0487 FJSC11DRAFT_0486 | UYIDRAFT_02338  UYIDRAFT_02340 |  |  |
| Short chain dehydrogenase |  | - | UYKDRAFT_03942 | FJSC11DRAFT_0485 | UYIDRAFT_02339 |  |  |

| Cluster 48: Orphan PKS | | | | | | | | |
| --- | --- | --- | --- | --- | --- | --- | --- | --- |
|  | FT PCC 7521 | FM PCC 7414 | FS PCC 9339 | FM SAG 1427-1 | FS JSC-11 | FS PCC 9431 | WI HT-29-1 | HW IC-52-3 |
| PKS (UNK-KS-AT-ACP) | UYKDRAFT_04200 | UYIDRAFT_02038 | PCC9339_04835 | UYGDRAFT_03296 | FJSC11DRAFT_1925 | - | - | - |
| PKS (KR) | UYKDRAFT_04199 | UYIDRAFT_02039 | PCC9339_04834 | UYGDRAFT_03297 | FJSC11DRAFT_1924 | - | - | - |
| PKS (KS) | UYKDRAFT_04198 | UYIDRAFT_02040 | PCC9339_04833 | UYGDRAFT_03298 | FJSC11DRAFT_1923 | Fis9431DRAFT_0816 | HT291_01137 | IC523_00630 |
| Acyl carrier protein | UYKDRAFT_04197 | UYIDRAFT_02041 | PCC9339_04832 | UYGDRAFT_03299 | FJSC11DRAFT_1922 | Fis9431DRAFT_0815 | HT291_01136 | IC523_00629 |
| Uncharacterised conserved protein | UYKDRAFT_04196 | UYIDRAFT_02042 | PCC9339_04831 | UYGDRAFT_03300 | FJSC11DRAFT_1921 | Fis9431DRAFT_0814 | HT291_01135 | IC523_00628 |
| Multidrug efflux pump | UYKDRAFT_04195 | UYIDRAFT_02043 | PCC9339_04830 | UYGDRAFT_03301 | - | - | - | - |
| Hypothetical protein | UYKDRAFT_04194 | UYIDRAFT_02044 | PCC9339_04829 | UYGDRAFT_03302 | - | - | - | - |
| Alpha/beta hydrolase family | UYKDRAFT_04193 | UYIDRAFT_02045 | PCC9339_04828 | UYGDRAFT_03303 | - | Fis9431DRAFT_0813 | HT291_01134 | IC523_00627 |

| Cluster 49: Orphan PKS | | | | | | | | | | | | | |
| --- | --- | --- | --- | --- | --- | --- | --- | --- | --- | --- | --- | --- | --- |
|  | PKS (KS-AT-UNK-KR) | PfaB family protein | PfaD family protein | Hypothetical protein | PKS (ACP) | Fatty acid desaturase | Hypothetical protein | Dehydrogenase/ | Aryl carrier domain | Hypothetical protein | Uncharacterized protein containing a NRPS condensation (elongation) domain | Epimerase | Uncharacterised protein conserved in bacteria |
| FS PCC 9339 | PCC9339DRAFT_00979 | PCC9339DRAFT_00978 | PCC9339DRAFT_00977 | PCC9339DRAFT_00976 | PCC9339DRAFT_00975 | PCC9339DRAFT_00974 | - | - | PCC9339DRAFT_00973 | PCC9339DRAFT_00972 | PCC9339DRAFT_00971  PCC9339DRAFT_00970 | PCC9339DRAFT_00969 | PCC9339DRAFT_00968 |
| CS PCC 9212 | UYEDRAFT_00855 | UYEDRAFT_00854 | UYEDRAFT_00853 | UYEDRAFT_00852 | UYEDRAFT_00851 | - | - | - | UYEDRAFT_00850 | UYEDRAFT_00849 | UYEDRAFT_00848  UYEDRAFT_00847  UYEDRAFT_00846 | UYEDRAFT_00845 | UYEDRAFT_00844 |
| CF PCC 6912 | UYCDRAFT_00567 | UYCDRAFT_00566 | UYCDRAFT_00565 | UYCDRAFT_00564 | UYCDRAFT_00563 | - | - | - | UYCDRAFT_00562 | UYCDRAFT_00561 | UYCDRAFT_00560  UYCDRAFT_00559  UYCDRAFT_00558 | UYCDRAFT_00557 | UYCDRAFT_00556 |
| FS JSC-11 | FJSC11DRAFT_3965 | FJSC11DRAFT_3964 | FJSC11DRAFT_3963 | FJSC11DRAFT_3962 | FJSC11DRAFT_3961 | - | FJSC11DRAFT_3960  FJSC11DRAFT_3959 | - | FJSC11DRAFT_3958 | - | - | - | - |
| FT PCC 7521 | UYKDRAFT_02244 | UYKDRAFT_02245 | UYKDRAFT_02246 | UYKDRAFT_02247 | UYKDRAFT_02248 | - | UYKDRAFT_02249  UYKDRAFT_02250 | - | UYKDRAFT_02251 | UYKDRAFT_0225 | - | - | - |
| FS PCC 9431 | Fis9431DRAFT_0253 | Fis9431DRAFT_0252 | Fis9431DRAFT_0251 | Fis9431DRAFT_0250 | Fis9431DRAFT_0249 | - | Fis9431DRAFT_0248  Fis9431DRAFT_0247 | Fis9431DRAFT_0246 | Fis9431DRAFT_0245 | Fis9431DRAFT_0244 | Fis9431DRAFT_0243  Fis9431DRAFT_0242 | Fis9431DRAFT_0241 | Fis9431DRAFT_0240 |
| HW IC-52-3 | IC523_02090 | IC523_02091 | IC523_02092 | IC523_02093 | IC523_02094 | - | IC523_02095 IC523_02096 | IC523_02097 | IC523_02098 | IC523_02099 | IC523_02100  IC523_02101 | IC523_02092 | IC523_02093 |
| WI HT-29-1 | HT291_03712 | HT291_03711 | HT291_03710 | HT291_03709 | HT291_03708 | - | HT291_03707  HT291_03706 | HT291_03705 | HT291_03704 | HT291_03703 | HT291_03702  HT291_03701 | HT291_03700 | HT291_03699 |
| FM SAG 1427-1 | UYGDRAFT_06348 | UYGDRAFT_06349 | UYGDRAFT_06350 | UYGDRAFT_06351 | UYGDRAFT_06352 |  | UYGDRAFT_06353  UYGDRAFT_06354 | UYGDRAFT_06355 | UYGDRAFT_06356 | UYGDRAFT_06357 | UYGDRAFT_06358  UYGDRAFT_06359 | UYGDRAFT_06360 | UYGDRAFT_06361 |
| FM PCC 7414 | UYIDRAFT_05479  UYIDRAFT_05480 | UYIDRAFT_05481 | UYIDRAFT_05482 | UYIDRAFT_05483 | UYIDRAFT_05484 | - | UYIDRAFT_05485  UYIDRAFT_05486  (End of scaffold) | - | - | - | - | - | - |

| Cluster 50: Orphan PKS | | | | | |  |  |
| --- | --- | --- | --- | --- | --- | --- | --- |
|  | PKS (KS-UNK-ACP-ACP) /PfaA | Hypothetical protein (DUF4480/ Carboxypeptidase M14 superfamily) | KR-DH domains | PKS (KS-KS-AT) /PfaB family protein | Dioxygenases related to 2-niropropane dioxygenase/ PfaD family protein | ACP-Red | Epimerase |
| FT PCC 7521 | UYKDRAFT_04235 | UYKDRAFT_04234 | UYKDRAFT_04233 | UYKDRAFT_04232 | UYKDRAFT_04231 | UYKDRAFT_04230 | UYKDRAFT_04239 |
| FS PCC 9339 | PCC9339DRAFT_00210 | PCC9339DRAFT_00209 | PCC9339DRAFT_00208 | PCC9339DRAFT_00207 | PCC9339DRAFT_00206 | PCC9339DRAFT_00205 | PCC9339DRAFT_00204 |
| FM SAG 1427-1 | UYGDRAFT_03685 | UYGDRAFT_03686 | UYGDRAFT_03687 | UYGDRAFT_03688 | UYGDRAFT_03689 | UYGDRAFT_03690 | UYGDRAFT_03691 |
| WI HT-29-1 | HT291_04062 | HT291_04063 | HT291_04064 | HT291_04065 | HT291_04066 | HT291_04068 | HT291_04069 |
| HW IC-52-3 | IC523_01788 | IC523_01789 | IC523_01790 | IC523_01791 | IC523_01792 | IC523_01793 | IC523_01794 |
| FS PCC 9431 | Fis9431DRAFT_3265 | Fis9431DRAFT_3266 | Fis9431DRAFT_3267 | Fis9431DRAFT_3268 | Fis9431DRAFT_3269 | Fis9431DRAFT_3270 | Fis9431DRAFT_3271 |
| FS PCC 9605 | FIS9605DRAFT_01283 | FIS9605DRAFT_01284 | FIS9605DRAFT_01285 | FIS9605DRAFT_01286 | FIS9605DRAFT_01287 | FIS9605DRAFT_01288 | FIS9605DRAFT_01289 |
| CF PCC 6912 | UYEDRAFT_03589 | UYEDRAFT_03590 | UYEDRAFT_03591 | UYEDRAFT_03592 | UYEDRAFT_03593 | UYEDRAFT_03594 | UYEDRAFT_03595 |
| CS PCC 9212 | UYCDRAFT_03678 | UYCDRAFT_03677 | UYCDRAFT_03676 | UYCDRAFT_03675 | UYCDRAFT_03674 | UYCDRAFT_03673 | UYCDRAFT_03672 |
| FM PCC 7414 | UYIDRAFT_04194  UYIDRAFT_04193 | UYIDRAFT_04192  UYIDRAFT_04191 | UYIDRAFT_04190 | UYIDRAFT_04189 | UYIDRAFT_04188 | UYIDRAFT_04187 | UYIDRAFT_04186 |
| FS JSC11 | FJSC11DRAFT_1963 | FJSC11DRAFT_1962 | FJSC11DRAFT_1961 | FJSC11DRAFT_1960  FJSC11DRAFT_1959 | FJSC11DRAFT_1958 | FJSC11DRAFT_1957 | FJSC11DRAFT_1956 |
| MR PCC 10914 | Mas10914DRAFT_3767 | Mas10914DRAFT_3766 | Mas10914DRAFT_3765 | Mas10914DRAFT_3764 | Mas10914DRAFT_3763 | Mas10914DRAFT_3767 | - |
| MT BC008 | YYIDRAFT_10253  End of scaffold | - | - | Beginning of scaffold YYIDRAFT_11222 | YYIDRAFT_11223 | YYIDRAFT_11224 | - |
