# Supplementary material for: Genome mining for natural product biosynthetic gene clusters in the Subsection V cyanobacteria
Source: BMC Genomics. 2015 Sep 3;16(1):669. doi: 10.1186/s12864-015-1855-z (PMC4558948; doi:10.1186/s12864-015-1855-z)
Supplement: Additional file 6: — Bacteriocin gene cluster and precursor analysis. (DOCX 103 kb) [file 12864_2015_1855_MOESM6_ESM.docx]

**Additional file 6: Bacteriocin gene clusters and precursor analysis**

Abbreviations: WI HT-29-1: *W. intricata* UH strain HT-29-1, HW IC-52-3: *H. welwitschii* UH strain IC-52-3, FS PCC 9431: *Fischerella* sp. PCC 9431, FS PCC 9399: *Fischerella* sp. PCC 9339, FM SAG 1427-1: *F. muscicola* SAG 1427-1, CF PCC 6912: *Chlorogloeopsis fritschii* PCC 6912, CS PCC 9212: *Chlorogloeopsis* sp. PCC 9212, MT BC008: *M. testarum* BC008. MR PCC 10914: *Mastigicladopsis repens* PCC 10914, FS PCC 9605: *Fischerella* sp. PCC 9605, FS JSC-11: *Fischerella* sp. JSC-11, FM PCC 7414: *Fischerella muscicola* PCC 7414, FT PCC 7521: *Fischerella thermalis* PCC 7521.

| Type I with HepP | | | | | |
| --- | --- | --- | --- | --- | --- |
|  | Putative HepP precursor | HlyD | Hypothetical protein | ABC transporter  (CAP_ED, C39, ABC mem, ABC ATPase) | SurA |
| WI HT-29-1 | HT291_02999 | HT291_03000 | - | HT291_03001 | - |
| HW IC-52-3 | IC523_01588 | IC523_01587 | - | IC523_01586 | - |
| FM PCC 7414 | UYIDRAFT_00311 | UYIDRAFT_00310 | UYIDRAFT_00309 | UYIDRAFT_00308 | - |
| FM SAG 1427-1 | UYGDRAFT_01271 | UYGDRAFT_01270 | - | UYGDRAFT_01269 | - |
| FS JSC-11 | FJSC11DRAFT_2277 | FJSC11DRAFT_2276 | - | FJSC11DRAFT_2275 | - |
| FS PCC 9339 | PCC9339DRAFT_04285 | PCC9339DRAFT_04286 | - | PCC9339DRAFT_04287 | - |
| FS PCC 9431 | Fis9431DRAFT_1960 | Fis9431DRAFT_1961 | - | Fis9431DRAFT_1962 | - |
| FS PCC 9605 | FIS9605DRAFT_02060 | FIS9605DRAFT_02061 | - | FIS9605DRAFT_02062 | - |
| FT PCC 7521 | UYKDRAFT_4432 | UYKDRAFT_4433 | - | UYKDRAFT_4434 | - |
| CS PCC 9212 | UYEDRAFT_04765 | UYEDRAFT_04764 | - | UYEDRAFT_04763 | - |
| CF PCC 6912 | UYCDRAFT_02092 | UYCDRAFT_02093 | - | UYCDRAFT_02094 | - |
| MR PCC 10914 | Mas10914DRAFT_1080 | - | - | - | - |
| MT BC008 | YYIDRAFT_06796 | YYIDRAFT_06800 | YYIDRAFT_06797  YYIDRAFT_06798 | YYIDRAFT_06799 | YYIDRAFT_06801 |

| Type I |  |  |  |  |  |
| --- | --- | --- | --- | --- | --- |
|  | SurA | ABC transporter (CAP_ED, C39, ABC mem, ABC ATPase) | Putative precursor | HlyD | Peptidase (M41) |
| WI HT-29-1 | HT291_02098 | HT291_02099 | HT291_02100 | HT291_02101 | HT291_02102 |
| HW IC-52-3 | IC523_03333 | IC523_03334 | - | IC523_03335 | IC523_03336 |
| FM PCC 7414 | UYIDRAFT_03886 | UYIDRAFT_03885 | - | UYIDRAFT_03884 | - |
| FS PCC 9339 | PCC9339DRAFT_02656 | PCC9339DRAFT_02657 | PCC9339DRAFT_02659 | PCC9339DRAFT_02658 | - |
| FM SAG 1427-1 | UYGDRAFT_05789 | UYGDRAFT_05788 | UYGDRAFT_05791 | UYGDRAFT_05787 | UYGDRAFT_05786 |
| FS JSC-11 | FJSC11DRAFT_4131 | FJSC11DRAFT_4130 | FJSC11DRAFT_4127 | FJSC11DRAFT_4129 | FJSC11DRAFT_4128 |
| FS PCC 9431 | Fis9431DRAFT_1377 | Fis9431DRAFT_1376 | Fis9431DRAFT_1375  Fis9431DRAFT_1373 | Fis9431DRAFT_1374 | Fis9431DRAFT_1372 |
| FS PCC 9605 | FIS9605DRAFT_03990 | FIS9605DRAFT_03991 | FIS9605DRAFT_03993  FIS9605DRAFT_03994 | FIS9605DRAFT_03992 | - |
| FT PCC 7521 | UYKDRAFT_00105 | UYKDRAFT_00106 | UYKDRAFT_00103 | UYKDRAFT_00107 | UYKDRAFT_00108 |

| Type I |  |  | |  | |  | |  |
| --- | --- | --- | --- | --- | --- | --- | --- | --- |
|  | SurA | | ABC transporter  (CAP_ED, C39, ABC mem, ABC ATPase) | | HlyD | | Putative precursor |  |
| WI HT-29-1 | HT291_02673 | | HT291_02674 | | HT291_02675 | | HT291_02676 |  |
| HW IC-52-3 | IC523_04195 | | IC523_04194 | | IC523_04193 | | IC523_04192 |  |
| FM PCC 7414 | UYIDRAFT_02215 | | UYIDRAFT_02214 | | UYIDRAFT_02213 | | UYIDRAFT_02212 |  |
| FS PCC 9339 | PCC9339DRAFT_04745 | | PCC9339DRAFT_04746 | | PCC9339DRAFT_04747 | | PCC9339DRAFT_04748 |  |
| FM SAG 1427-1 | UYGDRAFT_00468 | | UYGDRAFT_00467 | | UYGDRAFT_00466 | | UYGDRAFT_00465 |  |
| FS JSC-11 | FJSC11DRAFT_2626 | | FJSC11DRAFT_2627 | | FJSC11DRAFT_2628 | | FJSC11DRAFT_2629 |  |
| FS PCC 9431 | Fis9431DRAFT_0493 | | Fis9431DRAFT_0494 | | Fis9431DRAFT_0495 | | Fis9431DRAFT_0496 |  |
| FS PCC 9431 (2) | Fis9431DRAFT_5737 | | Fis9431DRAFT_5736 | | Fis9431DRAFT_5734 | | Fis9431DRAFT_5735 |  |
| FS PCC 9605 | FIS9605DRAFT_03407 | | FIS9605DRAFT_03406 | | FIS9605DRAFT_03405 | | FIS9605DRAFT_03404 |  |
| FS PCC 9605 (2) | FIS9605DRAFT_02549 | | FIS9605DRAFT_02548 | | FIS9605DRAFT_02547 | | - |  |
| FT PCC 7521 | UYKDRAFT_02608 | | UYKDRAFT_02607 | | UYKDRAFT_02606 | | UYKDRAFT_02605 |  |
| MR PCC 10914 | Mas10914DRAFT_0778 | | Mas10914DRAFT_0780 | | Mas10914DRAFT_0779 | | Mas10914DRAFT_0777  Mas10914DRAFT_0776  Mas10914DRAFT_0781  Mas10914DRAFT_0782 |  |
| MT BC008 | YYIDRAFT_12043 | | YYIDRAFT_12041 | | YYIDRAFT_12042 | | YYIDRAFT_12044 |  |
| MT BC008 | YYIDRAFT_08528 | | YYIDRAFT_08527 | | YYIDRAFT_08526 | | YYIDRAFT_08525 |  |

| Type I | |  | |  | |  | | |
| --- | --- | --- | --- | --- | --- | --- | --- | --- |
|  | Precursor | | Adjacent ORF | | SurA | | ABC transporter (CAP_ED, C39, ABC mem, ABC ATPase) | HlyD |
| CS PCC 9212 | UYEDRAFT_04500 | | UYEDRAFT_04505  UYEDRAFT_04504 | | UYEDRAFT_04503 | | UYEDRAFT_04502 | UYEDRAFT_04501 |
| CF PCC 6912 | UYCDRAFT_06567 | | UYCDRAFT_06572  UYCDRAFT_06571 | | UYCDRAFT_06570 | | UYCDRAFT_06569 | UYCDRAFT_06568 |

| Type II | | | |  | |  | | |  |  |  |  |
| --- | --- | --- | --- | --- | --- | --- | --- | --- | --- | --- | --- | --- |
|  | Precursor | Adjacent ORFs | SurA | | HlyD | | ABC transporter (CAP_ED, C39, ABC mem, ABC ATPase) | Transposase (COG3415) | | | | Other modification enzyme (M16) |
| WI HT-29-1 | HT291_02603  HT291_02604  HT291_02605 | HT291_02606  HT291_02607  HT291_02608 | HT291_02609  HT291_02610 | | HT291_02611 | | HT291_02612 | HT291_02613 | | | | - |
| HW IC-52-3 | IC523_04125  IC523_04124  IC523_04123  IC523_04122  IC523_04121  IC523_04117 | IC523_04120  IC523_04119  IC523_04118 | IC523_04116  IC523_04115 | | IC523_04114 | | IC523_04113 | IC523_04112 | | | | - |
| MR PCC 10914 | Mas10914DRAFT_0760  Mas10914DRAFT_0759  Mas10914DRAFT_0758  Mas10914DRAFT_0757 | Mas10914DRAFT_0763  Mas10914DRAFT_0762  Mas10914DRAFT_0761 | Mas10914DRAFT_0764  Mas10914DRAFT_0765 | | Mas10914DRAFT_0766 | | Mas10914DRAFT_0767 | Mas10914DRAFT_0768 | | | | Mas10914DRAFT_0756  Mas10914DRAFT_0755 |
| FS PCC 9339 | PCC9339DRAFT_04137  PCC9339DRAFT_04138  PCC9339DRAFT_04139  PCC9339DRAFT_04140  PCC9339DRAFT_04141  PCC9339DRAFT_04146 | - | PCC9339DRAFT_04142 | | PCC9339DRAFT_04143 | | PCC9339DRAFT_04144 | PCC9339DRAFT_04145 | | | | - |
| FS PCC 9605 | FIS9605DRAFT_06856  FIS9605DRAFT_06857  FIS9605DRAFT_06862 | - | FIS9605DRAFT_06858 | | FIS9605DRAFT_06859 | | FIS9605DRAFT_06860 | FIS9605DRAFT_06861 | | | |  |
| MT BC008 | YYIDRAFT_06878  YYIDRAFT_06873 | - | YYIDRAFT_06874 | | YYIDRAFT_06875 | | YYIDRAFT_06876 | YYIDRAFT_06877 | | | |  |

| Type II | | | | | | | |
| --- | --- | --- | --- | --- | --- | --- | --- |
|  | SurA | HlyD | ABC transporter (CAP_ED, C39, ABC mem, ABC ATPase) | Transposase (COG3415) | Adjacent ORF | ABC transporter (C39, ABC mem, ABC ATPase) | HlyD |
| MT BC008 | YYIDRAFT_08457 | YYIDRAFT_08456 | YYIDRAFT_08455 | YYIDRAFT_08454 | YYIDRAFT_08453  YYIDRAFT_08452 | YYIDRAFT_08451 | YYIDRAFT_08450 |

| Type II | | | |  | |  | |  | |  | |  |  |
| --- | --- | --- | --- | --- | --- | --- | --- | --- | --- | --- | --- | --- | --- |
|  | Putative precursor | SurA | ABC transporter (CAP_ED, C39, ABC mem, ABC ATPase) | | HlyD | | SurA | | Putative precursor | | Other modification enzymes (M16) | | |
| FS PCC 9605 | FIS9605DRAFT_01929  FIS9605DRAFT_01927 | FIS9605DRAFT_01928 | FIS9605DRAFT_01926 | | FIS9605DRAFT_01925 | | FIS9605DRAFT_01924 | | FIS9605DRAFT_01923  FIS9605DRAFT_01922  FIS9605DRAFT_01921  FIS9605DRAFT_01920  FIS9605DRAFT_01918 | | FIS9605DRAFT_01917  FIS9605DRAFT_01916 | | |
| FS PCC 9431 | Fis9431DRAFT_1605 | Fis9431DRAFT_1606 | Fis9431DRAFT_1607 | | Fis9431DRAFT_1608 | | Fis9431DRAFT_1609 | | Fis9431DRAFT_1610  Fis9431DRAFT_1611  Fis9431DRAFT_1612  Fis9431DRAFT_1613  Fis9431DRAFT_1614  Fis9431DRAFT_1615  Fis9431DRAFT_1616 Fis9431DRAFT_1617 | | - | | |
| FM SAG 1427-1 | UYGDRAFT_  06218  UYGDRAFT_  06216 | UYGDRAFT  _06217 | UYGDRAFT_  06215 | | UYGDRAFT_  06214 | | UYGDRAFT  _06213 | | UYGDRAFT_06212  UYGDRAFT_06211  UYGDRAFT_06210  UYGDRAFT_06209  UYGDRAFT_06208 | | - | | |
| FM PCC 7414 | UYIDRAFT_02116 | UYIDRAFT_02117 | UYIDRAFT_02118 | | UYIDRAFT_02119 | | UYIDRAFT_02120 | | UYIDRAFT_02121  UYIDRAFT_02122  UYIDRAFT_02123  UYIDRAFT_02124  UYIDRAFT_02125  UYIDRAFT_02126 | | - | | |
| FS PCC 9339 | PCC9339DRAFT_01115 | PCC9339DRAFT_01116 | PCC9339DRAFT_01117 | | PCC9339DRAFT_01118 | | PCC9339DRAFT_01119 | | PCC9339DRAFT_01120  PCC9339DRAFT_01121  PCC9339DRAFT_01122  PCC9339DRAFT_01123  PCC9339DRAFT_01124  PCC9339DRAFT_01125  PCC9339DRAFT_01126 | | - | | |
| WI  HT-29-1 | HT291_01196 | HT291_01197 | HT291_01198 | | HT291_01199 | | HT291_01200 | | HT291_01201  HT291_01202  HT291_01203  HT291_01204 | | - | | |
| HW  IC-52-3 | IC523_05295 | IC523_05296 | IC523_05297 | | IC523_05298 | | IC523_05299 | | IC523_05300 | | - | | |
| FT PCC 7521 | UYKDRAFT_  02526 | UYKDRAFT_  02527 | UYKDRAFT_  02528 | | UYKDRAFT_  02529 | | UYKDRAFT_  02530 | | UYKDRAFT_02531  UYKDRAFT_02532  UYKDRAFT_02533  UYKDRAFT_02534  UYKDRAFT_02535  UYKDRAFT_02536 | | - | | |
| FS JSC-11 | FJSC11DRAFT_  3629 | FJSC11DRAFT_  3628 | FJSC11DRAFT_  3627 | | FJSC11DRAFT_  3626 | | FJSC11DRAFT_  3625 | | FJSC11DRAFT_3624  FJSC11DRAFT_3623  FJSC11DRAFT_3622  FJSC11DRAFT_3621  FJSC11DRAFT_3620  FJSC11DRAFT_3619 | | - | | |

| Type III | | | |  |
| --- | --- | --- | --- | --- |
|  | Putative precursor | ABC transporter  (C39, ABC mem, ABC ATPase) | HlyD | |
| WI HT-29-1 | HT291_04123  HT291_04122  HT291_04121  HT291_04120  HT291_04119 | HT291_04118 | HT291_04117 | |
| HW IC-52-3 | IC523_04543  IC523_04542  IC523_04541  IC523_04540  IC523_04539 | IC523_04538 | IC523_04537 | |
| CS PCC 9212 | UYEDRAFT_01652  UYEDRAFT_01653  UYEDRAFT_01654 | UYEDRAFT_01655 | UYEDRAFT_01656 | |
| CF PCC 6912 | UYCDRAFT_01381  UYCDRAFT_01380  UYCDRAFT_01379 | UYCDRAFT_01378 | UYCDRAFT_01377 | |
| FM PCC 7414 | UYIDRAFT_01367  UYIDRAFT_01366  UYIDRAFT_01365  UYIDRAFT_01364  UYIDRAFT_01361 | UYIDRAFT_01363 | UYIDRAFT_01362 | |
| FS JSC-11 | FJSC11DRAFT_2140  FJSC11DRAFT_2141  FJSC11DRAFT_2142  FJSC11DRAFT_2143  FJSC11DRAFT_2144 | FJSC11DRAFT_2145 | FJSC11DRAFT_2146 | |
| FS PCC 9431 | Fis9431DRAFT_0200  Fis9431DRAFT_0199  Fis9431DRAFT_0198  Fis9431DRAFT_0197  Fis9431DRAFT_0196 | Fis9431DRAFT_0195 | Fis9431DRAFT_0194 | |
| FS PCC 9605 | FIS9605DRAFT_04080  FIS9605DRAFT_04079  FIS9605DRAFT_04078 | FIS9605DRAFT_04077 | FIS9605DRAFT_04076 | |
| FT PCC 7521 | UYKDRAFT_04574  UYKDRAFT_04573  UYKDRAFT_04572  UYKDRAFT_04571  UYKDRAFT_04570 | UYKDRAFT_04569 | UYKDRAFT_04568 | |
| FS PCC 9605 | FIS9605DRAFT_03511  FIS9605DRAFT_03512 | FIS9605DRAFT_03513 | FIS9605DRAFT_03514 | |
| CS PCC 9212 | UYEDRAFT_05691 | UYEDRAFT_05692 | UYEDRAFT_05693 | |
| CF PCC 6912 | UYCDRAFT_05976  UYCDRAFT_05977 | UYCDRAFT_05978 | UYCDRAFT_05979 | |
| MT BC008 | YYIDRAFT_08452 | YYIDRAFT_08451 | YYIDRAFT_08450 | |
| FM SAG1427-1 | UYGDRAFT_06406  UYGDRAFT_06405 | UYGDRAFT_06407 | UYGDRAFT_06408 | |

| Type III | | | |  |
| --- | --- | --- | --- | --- |
|  | Putative precursor | HlyD | ABC transporter  (C39, ABC mem, ABC ATPase) | |
| FM PCC 7414 | UYIDRAFT_02485 | UYIDRAFT_02486 | UYIDRAFT_02487 | |
| FS PCC 9339 | PCC9339DRAFT_02011 | PCC9339DRAFT_02010 | PCC9339DRAFT_02009 | |
| FT PCC 7521 | UYKDRAFT_02856 | UYKDRAFT_02855 | UYKDRAFT_02854 | |
| FS JSC-11 | FJSC11DRAFT_2417 | FJSC11DRAFT_2418 | FJSC11DRAFT_2419 | |
| CS PCC 9212 | UYEDRAFT_04803  UYEDRAFT_04802  UYEDRAFT_04801  UYEDRAFT_04800 | UYEDRAFT_04799 | UYEDRAFT_04798 | |
| CF PCC 6912 | UYCDRAFT_02053  UYCDRAFT_02054  UYCDRAFT_02055  UYCDRAFT_02056 | UYCDRAFT_02057 | UYCDRAFT_02058 | |
| MT BC008 | YYIDRAFT_05323  YYIDRAFT_05322 | YYIDRAFT_05321 | YYIDRAFT_05320 | |
| MT BC008 | YYIDRAFT_08840  YYIDRAFT_08839  YYIDRAFT_08838  YYIDRAFT_08837 | YYIDRAFT_08835 | YYIDRAFT_08834 | |

| Type IV | | |
| --- | --- | --- |
|  | LanM | Type 2 lantibiotic gene |
| HW IC-52-3 | IC523_04483 | - |
| MT BC008 | YYIDRAFT_12683 | - |
| FS PCC 9431 | Fis9431DRAFT_5422 | - |
| MR PCC 10914 | Mas10914DRAFT_3523 | - |
| CS PCC 9212 | UYEDRAFT_06004 | UYEDRAFT_06003 |
| CF PCC 6912 | UYCDRAFT_03447 | UYCDRAFT_03446 |

| Type IV | | | | | | | |
| --- | --- | --- | --- | --- | --- | --- | --- |
|  | Precursor | LanM | HlyD | ABC transporter  (C39, ABC mem, ABC ATPase) | ABC transporter (ABC mem, ABC ATPase) | Type 2 lantibiotic gene | HlyD |
| WI HT-29-1 | HT291_04027 | HT291_04026 | HT291_04025 | HT291_04024  HT291_04023 | HT291_04022 | - | - |
| HW IC-52-3 | IC523_04136 | IC523_04137 | IC523_04138 | IC523_04139 | IC523_04140 | - | - |
| CS PCC 9212 | UYEDRAFT_01784 | UYEDRAFT_01783 | UYEDRAFT_01782 | - | - | - | - |
| CF PCC 6912 | UYCDRAFT_01250 | UYCDRAFT_01251 | UYCDRAFT_01252 | - | - | - | - |
| FT PCC 7521 | - | UYKDRAFT_03165  UYKDRAFT_03166 | - | UYKDRAFT_03167 | - | UYKDRAFT_03168 | UYKDRAFT_03169 |
| FM SAG 1427-1 | UYGDRAFT_01907 | UYGDRAFT_01908 | - | UYGDRAFT_01909 | - | - | UYGDRAFT_01910 |
| FS JSC-11 | FJSC11DRAFT_0766 | FJSC11DRAFT_0764  FJSC11DRAFT_0765 | - | - | - | - | FJSC11DRAFT_0767 |

| Type IV | | | | | | | | |
| --- | --- | --- | --- | --- | --- | --- | --- | --- |
|  | S8 peptidase | Putative precursor | LanM | Adjacent ORFs | Other modification enzymes (CAP_ED, CAP_ED) | HlyD | ABC transporter (C39, ABC mem, ABC ATPase) | ABC transporter (ABC mem, ABC ATPase) |
| FS PCC 9605 | FIS9605DRAFT_04160 | FIS9605DRAFT_04159 | FIS9605DRAFT_04158 | FIS9605DRAFT_04157  FIS9605DRAFT_04156  FIS9605DRAFT_04155  FIS9605DRAFT_04154 | FIS9605DRAFT_04153 | FIS9605DRAFT_04152 | FIS9605DRAFT_04151 | FIS9605DRAFT_04150 |

| Type V | | |  | |  | |  |  | |  |  |  |
| --- | --- | --- | --- | --- | --- | --- | --- | --- | --- | --- | --- | --- |
|  | Precursor | Other modification enzyme (CAP_ED, CAP_ED) | | HlyD | | ABC transporter (C39, ABC mem, ABC ATPase) | | | ABC transporter (CAP_ED, ABC mem, ABC ATPase) | | | |
| WI HT-29-1 | HT291_02602  HT291_02601  HT291_02600  HT291_02599  HT291_02598  HT291_02597  HT291_02596  HT291_02595  HT291_02594  HT291_02593 | HT291_02592 | | HT291_02591 | | HT291_02590 | | | HT291_02589 | | | |
| HW IC-52-3 | IC523_06028  IC523_06027  IC523_06026  IC523_06025  IC523_06024  IC523_06023  IC523_06022  IC523_06021 | IC523_06020 | | IC523_06019  (Beginning of scaffold) | | (End of scaffold) IC523_04604 | | | IC523_04605 | | | |
| FS PCC 9431 | Fis9431DRAFT_5565  Fis9431DRAFT_5564  Fis9431DRAFT_5563  Fis9431DRAFT_5562  Fis9431DRAFT_5561  Fis9431DRAFT_5560  Fis9431DRAFT_5559 | Fis9431DRAFT_5558 | | Fis9431DRAFT_5557 | | Fis9431DRAFT_5556 | | | Fis9431DRAFT_5555 | | | |
| FM SAG 1427-1 | UYGDRAFT_04036  UYGDRAFT_04037  UYGDRAFT_04038 | UYGDRAFT_04039 | | UYGDRAFT_04040 | | UYGDRAFT_04041 | | | UYGDRAFT_04042 | | | |
| FS PCC 9339 | PCC9339DRAFT_05361  PCC9339DRAFT_05360  PCC9339DRAFT_05359  PCC9339DRAFT_05358  PCC9339DRAFT_05357  PCC9339DRAFT_05356 | PCC9339DRAFT_05355 | | PCC9339DRAFT_05354 | | PCC9339DRAFT_05353 (C39, ABC mem)  PCC9339DRAFT_05354 (ABC ATPase) | | | PCC9339DRAFT_05351 | | | |
| FS PCC 9605 | FIS9605DRAFT_01820  FIS9605DRAFT_01821  FIS9605DRAFT_01822  FIS9605DRAFT_01823 | FIS9605DRAFT_01824 | | FIS9605DRAFT_01825 | | FIS9605DRAFT_01826 | | | FIS9605DRAFT_01827 | | | |
| MT BC008 | - | YYIDRAFT_08465 | | YYIDRAFT_08464 | | YYIDRAFT_08463 | | | YYIDRAFT_08462  (ABC mem, ABC ATPase) | | | |

| Type VI | | |  | |  | |  | |  | |  |  |
| --- | --- | --- | --- | --- | --- | --- | --- | --- | --- | --- | --- | --- |
|  | Putative precursor or HlyD | S8 peptidase | | Other modification enzymes (DUF92) | | SurA | | ABC transporter (C39, ABC mem, ABC ATPase) | | Putative precursor | | |
| WI HT-29-1 | HT291_04430  HT291_04431  HT291_04432 | HT291_04433 | | HT291_04434 | | HT291_04435 | | HT291_04436 | | HT291_04437  HT291_04438 | | |
| HW IC-52-3 | - | IC523_00649 | | IC523_00650 | | IC523_00651 | | IC523_00652 | | IC523_00653  IC523_00654 | | |
| FS PCC 9431 | Fis9431DRAFT_0836 | Fis9431DRAFT_0837 | | Fis9431DRAFT_0838 | | Fis9431DRAFT_0839 | | Fis9431DRAFT_0840 | | - | | |
| FM SAG 1427-1 | UYGDRAFT_02153 | - | | UYGDRAFT_02154 | | UYGDRAFT_02155 | | UYGDRAFT_02156 | | - | | |
| FS PCC 9339 | PCC9339DRAFT_03806 | PCC9339DRAFT_03807 | | PCC9339DRAFT_03808 | | PCC9339DRAFT_03809 | | PCC9339DRAFT_03810 | | - | | |
| FM PCC 7414 | UYIDRAFT_04893 | UYIDRAFT_04894 | | UYIDRAFT_04895 | | UYIDRAFT_04896 | | UYIDRAFT_04897 | | UYIDRAFT_04898 | | |
| FT PCC 7521 | - | UYKDRAFT_00399 | | UYKDRAFT_00400 | | UYKDRAFT_00401 | | UYKDRAFT_00402  (with CAP_ED) | | UYKDRAFT_00403 | | |
| FS JSC-11 | - | FJSC11DRAFT_1145 | | FJSC11DRAFT_1146 | | FJSC11DRAFT_1147 | | FJSC11DRAFT_1148 (with CAP_ED) | | FJSC11DRAFT_1149 | | |
| FS PCC 9605 | FIS9605DRAFT_00599 | FIS9605DRAFT_00598 | | FIS9605DRAFT_00597 | | FIS9605DRAFT_00596 | | FIS9605DRAFT_00595 | | FIS9605DRAFT_00594 | | |
| MR PCC 10914 | Mas10914DRAFT_4746 | Mas10914DRAFT_4745 | | Mas10914DRAFT_4747 | | Mas10914DRAFT_4748 | | Mas10914DRAFT_4749 (with CAP_ED) | | - | | |
| CS PCC 9212 | UYEDRAFT_03957  (HlyD) | UYEDRAFT_03958 | | UYEDRAFT_03959 | | UYEDRAFT_03960 | | UYEDRAFT_03961 (with CAP_ED) | | - | | |
| CF PCC 6912 | UYCDRAFT_06818 (HlyD) | UYCDRAFT_06819 | | UYCDRAFT_06820 | | UYCDRAFT_06821 | | UYCDRAFT_06822 (with CAP_ED) | | - | | |

| Unclassified | | |  |  | |  | |  |  |  |
| --- | --- | --- | --- | --- | --- | --- | --- | --- | --- | --- |
|  | SurA | ABC transporter (CAP_ED/C39, ABC mem, ABC ATPase) | | | HlyD | | Precursor | | | |
| CS PCC 9212 | - | UYEDRAFT_02840 | | | UYEDRAFT_02841 | | - | | | |
| CF PCC 6912 | - | UYCDRAFT_04428 | | | UYCDRAFT_04427 | |  | | | |
| CF PCC 6912 | UYCDRAFT_05942 | UYCDRAFT_05943 | | | - | | - | | | |
| CS PCC 9212 | UYEDRAFT_05656 | UYEDRAFT_05657 | | | - | | - | | | |
| MT BC008 | YYIDRAFT_10912 | YYIDRAFT_10913 | | | - | | - | | | |
| FS PCC 9339 | - | PCC9339DRAFT_05380 | | | PCC9339DRAFT_05381 | | - | | | |
| FS PCC 9605 | - | FIS9605DRAFT_04264 | | | FIS9605DRAFT_04265 | | FIS9605DRAFT_04266 | | | |
| FM PCC 7414 | - | UYIDRAFT_05230 | | | - | | UYIDRAFT_05231 | | | |
| MR PCC 10914 | - | Mas10914DRAAFT_1490 | | | - | | Mas10914DRAAFT_1491 | | | |

MC7420_4637 ---MTYNMPT-INAKREKTLDPEQFEQIIDAIRQGKYSWACVLLLRFAGHNPQYYIPYRTYNRLRKEHSSQKPQENLQSGDTSGN----------PGQ

AM1_4010 ---MNHNLSTHSGTDFDKQITYEQFNQVIEAILMGKYSWACVLMLRFLGYNPLHYIPYRTYNRLIKKESLLSYQTSKDKASCKDF-------EPNSLI

YYIDRAFT_06796 ---MNQDIAGSSSNNLGKTLNSEQFDRVIEAILAGKYSWACVLMLRFTGYNPLHYIPYRTYNRLLKENTPGKKSKNQQQNNNVKVARENSTKNNQRN-

alr2818 ---MNQNTT-G-ITNYNKAINPQQFDKVVEAILAGKYSWACVLMLRFAGYNPMHYIPYRTYNRLLKENSEASKV-QQQQHDNLKNSQVAAVSRSNTN-

Ava_1098 ---MNQNTT-G-ITNYNKAINPQQFDKVVEAILAGKYSWACVLMLRFAGYNPMHYIPYRTYNRLLKENSEASKV-QQPQHDNLKNSQVAAVSRSNTN-

Aazo_0724 ---MNQDISGK-SSNLEKKINPEQFDQVIEAILAGKYSWACVLMLRFVGYNPLHYIPYRTYNRLLKENSRISRS-NTQQNESLKLAKPATEKRCDTH-

Mas10914DRAFT_1080 ---MNQSIS-G-SSNLDKKLHPEQFDQVVEAILAGKYSWACVLLLRFAGYNPLHYIPYRTYNRLLKENSQVGRS-NKHQNENIKIAKLPSDNRSESRV

UYEDRAFT_04765 ---MHQN---I-TSNFDKSINPEQLDQVVEAILAGKYSWACVLMLRFAGYNPLHYIPYRTYNRLIKENSQIKRA-SQQRTENLKIAALPSDQKSDSP-

UYCDRAFT_02092 ---MHQN---I-TSNFDKSINPEQLDQVVEAILAGKYSWACVLMLRFAGYNPLHYIPYRTYNRLIKENSQIKRA-SQQRTENLKIAALPSDQKSDSP-

FIS9605DRAFT_02060 MNTMNQDIPSN-ISNLEKTLNSEQFDQLVEAILAGKYSWACVLMLRFAGYNPLHYIPYRTYNRLIKENSQLKRA-SQQRNENLKIAKLPSDKRSDSN-

PCC9339DRAFT_04285 ---MNQNISSA-TSNFEKTLNPEQFDQLVEAILAGKYSWACVLILRFAGYNPLHYIPYRTYNRLIKENSQLKRV-NQQRNDNLKVAKLPSEKKSDSN-

HT291_02999 ---MNQNISSA-TSNFEKTLNPEQFDQLVEAILAGKYSWACVLILRFAGYNPLHYIPYRTYNRLIKENSQLKRV-NQQRNDNLKVAKLPSEKKSDSN-

IC523_01588 ---MNQNISSA-TSNFEKTLNPEQFDQLVEAILAGKYSWACVLILRFAGYNPLHYIPYRTYNRLIKENSQLKRV-NQQRNDNLKVAKLPSEKKSDSN-

Fis9431DRAFT_1960 ---MNQNISSA-TSNFEKTLNPEQFDQLVEAILAGKYSWACVLILRFAGYNPLHYIPYRTYNRLIKENSQLKRV-NQQRNDNLKVAKLPSEKKSDSN-

UYGDRAFT_01271 ---MNQNISSA-TSNFEKTLNPEQFDQLVEAILAGKYSWACVLILRFAGYNPLHYIPYRTYNRLIKENSQLKRV-NQQRNDNLKIAKLPSEKKSDSN-

UYIDRAFT_00310 ---MNQNVSSA-TSNFEKTLNPEQFDQLVEAILAGKYSWACVLILRFAGYNPLHYIPYRTYNRLIKENSQLKRV-NQQRNENLKIAKLPAEKKSDSN-

FJSC11DRAFT_2277 ---MNQNISSA-TSNFEKTLNPEQFDQLVEAILAGKYSWACVLILRFAGYNPLHYIPYRTYNRLIKENSQLKRV-NQQRNENLKIAKLPSEKKSDSN-

UYKDRAFT_04432 ---MNQNISSA-TSNFEKTLNPEQFDQLVEAILAGKYSWACVLILRFAGYNPLHYIPYRTYNRLIKENSQLKRV-NQQRKENLKVAKLPSEKKSDSN-

CY0110_11572 ---MSQSLYQ-QKNQVTKSMTEQQFEEIVDAILAGKYSWACVLILQTAGYNPLHYIPYRTYNRLIKDNRLKKRD-SEQQKTIKPSP--------KNSV

cce_2677 -MTMSNSGYS-ANSKQSRTMTEEQFEKIVDAILAGKYSWACVLILQTAGYNPLHYIPYRTYNRLIKDNCLKQSH-KKEKNEQRNFQKQQLSKDSCQQT

PCC8801_3266 ----MNRRFSNESGKTDKVMKEEQFEEIVAAILNGKYSWACVLILKFAGYNPLHYIPYRTYNRLIKDNCLNKPS-SQEKNQKTLN-KSSDDNG--DEF

Cyan8802_2855 ----MNRRFSNESGKTDKVMKEEQFEEIVAAILNGKYSWACVLILKFAGYNPLHYIPYRTYNRLIKDNCLNKPS-SQEKNQKTLN-KSSDDNG--DEF

PCC7424_3517 ---MRQQDFSNRFSKKDQVMTQEQFEKIVEAILAGKYSWACVLILRFAGYNPLHYIPYRTYNRLIKDNCRENSS-NQEQNQQKKP-NSDLSSRYYNNS

. : : :*::.:: ** *********:*: *:** :********** *.. .

KIxDLxYLExxxxxxxxxxGG

MC7420_4637 PTRKSSCKIADLNHLEVVDHKTLSMKGGYNLQGLPAIE-----SEDCIPDSVAPSDDWFSGVSSLFNRLTGLG----

AM1_4010 TPSNSPRYMQDLEYLEEVREKSTQVTGGTSNN----WLAKNYLSMWNKKSSNPNL----------------------

YYIDRAFT_06796 VTPSCLNQIKDLPYLEVVGKQKTQINGAKLEH----WLGKKINEYQSKKSEVKSATGSSTNPS---LKLCNLN----

alr2818 MPSSCLSKIKDLAYLEVVGKQTTEIHGGNLDQ----WLTEQVHEFQDMYLEPQAIS--NQDIT---FKLSDLDFIHN

Ava_1098 MPSSCLSKIKDLAYLEVVGKQTTEIHGGNLDQ----WLTEQVHEFQDMYLEPQAIS--NQDIT---FKLSDLDFIHN

Aazo_0724 LASACLSKIKDIAYLEVGGKQKAEVRGSHREK----KSA--------------------------------------

Mas10914DRAFT_1080 TPTSCLSKIKDIAYLEVVGKKKTEIRGGNLEQ----WLTTQVHEYESTNSEIAPET--INIFP--------------

UYEDRAFT_04765 TQKSCLSKIKDLAYLEVVGKKKTEIRGGSSER----WLATQIHEHQSIKPELKSDT--TQDSS---FKFSAFN----

UYCDRAFT_02092 TQKSCLSKIKDLAYLEVVGKKKTEIRGGSSER----WLATQIHEHQSIKPELKSDT--TQDSS---FKFSAFN----

FIS9605DRAFT_02060 PAQSCLSKIKDLAYLEVVGKQKREIRGGNQDQ----WLTTQIREYQSIKSELKPES--TQDLS---LKFCEFNYNIS

PCC9339DRAFT_04285 PPQSCLSKIKDLAYLEVVGKQKREIRGGHQDQ----WLATQVHEYESIQAELRPDK--TQDLS---FKYCEAN----

HT291_02999 PSQSCLSKIKDLAYLEVVGKQKREIRGGHQDQ----WLATQVHEYQSIQAELRPEK--TQDLS---FKYCEAN----

IC523_01588 PSQSCLSKIKDLAYLEVVGKQKREIRGGHQDQ----WLATQVHEYQSIQAELRPEK--TQDLS---FKYCEAN----

Fis9431DRAFT_1960 PSQSCLSKIKDLAYLEVVGKQKREIRGGHQDQ----WLATQVHEYQSIQAELRPEK--TQDLS---FKYCEAN----

UYGDRAFT_01271 PSQSCLSKIKDLAYLEVVGKQKREIRGGHQDQ----WLATQVHECQSIQAELRPDK--TQDLS---FKYCEAN----

UYIDRAFT_00310 PSQSCLSKIKDLAYLEVVGKQKREIRGGHQNQ----WLGTQIREYQSMEAELKPES--TQDLP---FKFCEIN----

FJSC11DRAFT_2277 PSQSCLSKIKDLAYLEVVGKQKREIRGGHQNQ----WLGTQIREYQSMQTELKPES--NQDLS---FKFCEIN----

UYKDRAFT_04432 PSQSCLSKIKDLAYLEVVGKQKREIRGGHQNQ----WLGTQIREYQSMQAELKPES--NQDLS---FKFCEIN----

CY0110_11572 TKHRTSTKISNLSYVESLDESESNSLGGLGCNQSSIWSIFFWNH---------------------------------

cce_2677 TTQTSREKIKDINYLEELTNNTAKIHGGFRLQ----LWQF-------------------------------------

PCC8801_3266 HRISYHQKIRDLSYLESVDKKAVKVAGGFRYF----WW---------------------------------------

Cyan8802_2855 HRISYHQKIRDLSYLESVDKKAVKVAGGFRYF----WW---------------------------------------

PCC7424_3517 SSYRDNSKIKDLNYLEECNQKEKLMAGSCGEK----WRIFS------------------------------------

: :: ::* .. *.

**Sequence alignment of putative HetP precursors.** The putative leader peptide cleavage motif is highlighted in the box. Putative HetP precursors from *Westiella intricata* UH strain HT-29-1 (HT291_02999), *Hapalosiphon welwitschii* UH strain IC-52-3 (IC523_01588), *Fischerella* sp. PCC 9605 (FIS9605DRAFT_02060), *Fischerella* sp. PCC 9431 (Fis9431DRAFT_1960), *Fischerella* sp. PCC 9339 (PCC9339DRAFT_04285), *Fischerella* sp. JSC-11 (FJSC11DRAFT_2277), *Fischerella muscicola* SAG 1427-1 (UYGDRAFT_01271), *Fischerella muscicola* PCC 7414 (UYIDRAFT_00310), *Fischerella thermalis* PCC 7521 (UYKDRAFT_04432), *Chlorogloeopsis fritschii* PCC 9212 (UYEDRAFT_04765), *Chlorogloeopsis fritschii* PCC 6912 (UYCDRAFT_02092), *Mastigocladopsis repens* PCC 10914 (Mas10914DRAFT_1080), *Mastigocoleus testarum* BC008 (YYIDRAFT_06796), *Anabaena variabilis* ATCC 29413 (Ava_1098), *Nostoc* sp. PCC 7120 (alr2818), *Nostoc azollae* 0708 (Aazo_0724), *Acaryochloris marina* MBIC11017 (AM1_4010), *Cyanothece* sp. PCC 8801 (PCC8801_3266), *Cyanothece* sp. PCC 8802 (Cyan8802_2855), *Cyanothece* sp. PCC 7424 (PCC7424_3517), *Cyanothece* sp. ATCC 51142 (cce_2677), *Cyanothece* sp. CCY0110 (CY0110_11572) and *Microcoleus chthonoplastes* PCC 7420 (MC7420_4637).

YYIDRAFT_07042 -----MPNPFKYLALGLIWVYRNAISPLLGPRCRHVPTCSDYAQEAITRFGVWRGGWLAVSRILRCHPWGTQGLDPVPEEVPHASALTPWKYGRWTGAHI---RDRFPDD----------

S7335_3409 MLF-SRFYEMKLTLLLLVKGYRQLISPLFPPMCRFEPTCSRYALDAIDRFGPLQGTWLTAKRFCRCHPLHPGGYDPVPEKKSVL------------------------------------

Aazo_0083 ---------MKILLIWIIKGYRLFVSPLFPPTCRFQPTCSMYAIQAIERFGLLRGGWMATLRILRCHPFHPGGYDPVPGLAEK-----SCCDHHD-------------------------

Ava_4222 ----------------------MFISPLFPPTCRFQPTCSMYALEAIERFGVFRGGWMGIRRILRCHPFHPGGYDPVPEVGEH------CCHHDSGK-----------------------

asr1611 ---------MKQIFIWLIKGYRMFISPLYPPTCRFRPTCSMYAIEAIERFGVFRGGWMAIRRILRCHPFHPGGYDPVPELGEH------CCHHDSGNKG---------------------

N9414_07129 ---------MKLLFIWLIKGYRMFISPLFPPTCRFQPTCSMYAIEAIERFGIWRGGWMATRRILRCHPFHPGGYDPVPEVKHN------CCDQHLSDSGKQTTEDHH--KG---------

UYCDRAFT_04502 ---------MKILLIWLIRFYRIFISPMFLPTCRFQPTCSAYALEAIERFGVWRGSWLAIRRILRCHPFHPGGYDPVPEITEQTKHHC--------------------------------

UYEDRAFT_02765 ---------MKILLIWLIRFYRIFISPMFLPTCRFQPTCSAYALEAIERFGVWRGSWLAIRRILRCHPFHPGGYDPVPEITEQTKHHC--------------------------------

Mas10914DRAFT_5362 ---------MKLLLILLIRFYRMFISPLFLPTCRFQPTCSMYAIQAIERFGVWRGSWLAIRRILRCHPFHPGGYDPVPEALGTATKQSSCCQHHE-------------------------

HT291_01132 ---------MKLLLIWLIRFYRMFISPLFLPTCRFQPTCSMYAIQAIERFGPWRGSWLAMRRILRCHPFHPGGYDPIPEGDKGDK---GDT-GDKGDKGDKGDKGDKGDKGEKLTTNS--

IC523_00625 ---------MKLLLIWLIRFYRMFISPLFLPTCRFQPTCSMYAIQAIERFGPWRGSWLAMRRILRCHPFHPGGYDPVPEGDKGDK---GDK-GDKEDK------------GEKLTTNS--

Fis9431DRAFT_0811 ---------MKLLLIWLIRFYRMFISPLFLPTCRFQPTCSMYAIQAIERFGPWRGSWLAMRRILRCHPFHPGGYDPVPEGDKGDK-------GDKEDK------------GEKLTTNS--

PCC9339DRAFT_04326 ---------MKLLLIWLIRFYRVFISPLFLPTCRFQPTCSMYAIQAIESFGPWRGSWLAIRRILRCHPFHPGGYDPVPEVENGER---GHGKGDKGDKEDKGDKGDKEDMGRETREKKLP

UYIDRAFT_01579 ---------MKLLLIWLIRFYRMFISPLFLPTCRFQPTCSMYAIQAIERFGALRGSWLAVRRILRCHPFHPGGYDPVPEVEE--------------------------------------

FJSC11DRAFT_3253 ---------MKLLLIWLIRFYRMFISPLFLPTCRFQPTCSMYAIQAIERFGPLRGSWLAVRRILRCHPFHRGGYDPVPEVEE--------------------------------------

UYKDRAFT_01396 ---------MKLLLIWLIRFYRMFISPLFLPTCRFQPTCSMYAIQAIERFGPLRGSWLAVRRILRCHPFHRGGYDPVPEVEE--------------------------------------

UYGDRAFT_01578 ---------MKLLLIWLIRFYRMFISPLFLPTCRFQPTCSMYAIQAIEKFGPWRGSWLAIRRILRCHPFHPGGYDPVPEVEKQTK---DCCYKEMG------------------------

FIS9605DRAFT_01955 ----------------------------------------MYAIQAIERFGPCRGSWLAIRRILRCHPFHPGGYDPVPEVEE--------------------------------------

*. :** .*:* *: *: **** * **:*

**Sequence alignment of putative DUF37 precursors.** The putative leader peptide cleavage motif is highlighted in the box. Putative D37 precursors from *Westiella intricata* UH strain HT-29-1 (HT291_01132), *Hapalosiphon welwitschii* UH strain IC-52-3 (IC523_00625), *Fischerella* sp. PCC 9605 (FIS9605DRAFT_01955), *Fischerella* sp. PCC 9431 (Fis9431DRAFT_0811), *Fischerella* sp. PCC 9339 (PCC9339DRAFT_04326), *Fischerella* sp. JSC11 (FJSC11DRAFT_3253), *Fischerella muscicola* SAG 1427-1 (UYGDRAFT_01578), *Fischerella muscicola* PCC 7414 (UYIDRAFT_01579), *Fischerella thermalis* PCC 7521 (UYKDRAFT_01396), *Chlorogloeopsis fritschii* PCC 9212 (UYEDRAFT_02765), *Chlorogloeopsis fritschii* PCC 6912 (UYCDRAFT_04502), *Mastigocladopsis repens* PCC 10914 (Mas10914DRAFT_5362), *Mastigocoleus testarum* BC008 (YYIDRAFT_07042), *Anabaena variabilis* ATCC 29413 (Ava_4222), *Nostoc* sp. PCC 7120 (asr1611), *Nostoc azollae* 0708 (Aazo_0083), *Nodularia spumigena* CCY9414 (N9414_07129) and *Synechococcus* sp. PCC 7335 (S7335_3409).

Fis9431DRAFT_2340 MSKQEVIRLFKNAQTNPNLRQVLNSASNLEAFVQIAHQHGYNFTVEEWQKATGLVIEDSESKVYRNQSS---------

HT291_00567 MSKQEVIRLFKNAQTNPNLRQVLNSASNLEAFVQIAHQHGYNFTVEEWQKATGLVIEDSESKVYRNQSS---------

IC523_00830 MSKQEVIRLFKNAQTNPNLRQVLNSASNLEAFVQIAHQHGYNFTVEEWQKATGLVIEDSESKVYRNQSS---------

UYGDRAFT_02065 MSKQEVIRLFRNAQTNPNLRKILNSASNLEAFVQMAQQQGYNFTLEEWQKATGLLTENLESKVYENQGS---------

UYIDRAFT_00914 MSKQEVIRLFRAAQTNPNLRETLNSASDLEAFVQMAQQQGYNFTVEEWQKATGLVMEESESQVSEIQGKYEG------

FJSC11DRAFT_3026 MSKQEVIRLFRAAQTNPNLREILNSASDLETFVKMAQQQGYNFTVDEWQKATGLVMEDSESQVSEIQGKCEG------

UYKDRAFT_01108 MSKQEVIRLFRAAQTNPNLREILNSASDLETFVKMAQQQGYNFTVDEWQKATGLVMEDSESQVSEIQGKCEG------

UYCDRAFT_05865 MSQASVKEFLATAKQDEAIRQQLQSAMTVHGCVKVAQESGYDFTAEELQSQLNEMSEEEVAEIV-NPGVAPRRHIQPQ

UYEDRAFT_05579 MSQASVKEFLATAKQDEAIRQQLQSAMTVHGCVKVAQESGYDFTAEELQSQLNEMSEEEVAEIV-NPGVAPRRHIQPQ

YYIDRAFT_11082 MIQENAARIYKQVEQAHAQQERQKALSNPEDFILLAAARGYNFNVKDLETQLSQLSDEDVAGIF-NPGIGPRRHLFPR

UYGDRAFT_05877 MTQQNAAQFLKAVKENQALKERLNATENPEAFIKIAKESGYDFSEEELEREISQLSDEDLAAIV-NPGWGTRRHIHPR

PCC9339DRAFT_06667 MTQQNAAQFLKAVKENQALKERLNATENPEAFIKIAKESGYDFSEEELEREISQLSDEDLAAIV-NPGWGTRRHIHPR

FIS9605DRAFT_05843 MAQKNAAELFKAVKEDQSLKERLRAATDPEAFIKVASLAGYNFTVSELQTELSKMSAEELAAIV-NPGVAPRLHIVPR

UYIDRAFT_02600 MSQKHAAQLFKAIKQDQVLKERLKAASDPEGFIKIAQERGYYFTVEELETELSKLSPEEMAAIV-NPGVAPRLHIYPR

FJSC11DRAFT_1770 MSQKHAAQLFKAVKEDQVLKERLKAASDPEGFIKIAQERGYHFTIEELETELSKLSCEELASIV-NPGVAPRLHIYPR

UYKDRAFT_04037 MSQKHAAQLFKAVKEDQVLKERLKAASDPEGFIKIAQERGYHFTIEELETELSKLSCEELASIV-NPGVAPRLHIYPR

Mas10914DRAFT_3891 MTQNNAAQLFKAVRQDQALQQRLKAATNPEAFITIAKERGFDFTVEELETEISKLSEEELAAIV-NPGIAPRTHIYPR

UYCDRAFT_02201 MTQKNAAQLFKAVKQDQALKARLKAAADPEAFIKIAQERGYNFTVTELQTELSQLSSEEVAAIV-NPGIAPRLHIYPR

UYEDRAFT_06592 MTQKNAAQLFKAVKQDQALKARLKAAADPEAFIKIAQERGYNFTVTELQTELSQLSSEEVAAIV-NPGIAPRLHIYPR

UYGDRAFT_03815 MTQNHAAQLFKAVKQDQALKERLKAAENPETFIQIAQERGYDFTVEELQTELSKLSSEELAAIV-NPGVAPRLHIYPR

HT291_01854 MIQNNAAQLFKAVTQDQALKERLKAAANPETFIQIAQERGYDFTVEELETELSKLSSEELAAIV-NPGVAPRLHIYPR

PCC9339DRAFT_03590 MTQNNAAQLFKAVKQDQALKERLKAAANPEAFIQIAQERGYDFTVEELETELSKLSSEELAAIV-NPGVRPRLHIYPR

Fis9431DRAFT_1465 MTQNNAAQLFKAVKQDQALKERLKAAANPETFIQIAQERGYDFTVEELETELSKLSSEELAAIV-NPGVAPRLHIYPR

IC523_03507 MTQNNAAQLFKAVKQDQALKERLKAAANPETFIQIAQERGYDFTVEELETELSKLSSEELAAIV-NPGVAPRLHIYPR

* : . .:: : :: *.:: . : :* *: *: * : : : : : .

**Sequence alignment of putative N11P precursor peptides from Subsection V cyanobacteria**. The conserved region near the peptide cleavage site is highlighted in the box. The double-glycine motif is absent in the N11P precursor peptides from the group V cyanobacteria. Putative N11P precursor peptides from *Westiella intricata* UH strain HT-29-1 (HT291_00567 and HT291_01854), *Hapalosiphon welwitschii* UH strain IC-52-3 (IC523_00830 and IC523_03507), *Fischerella* sp. PCC 9605 (FIS9605DRAFT_05843), *Fischerella* sp. PCC 9431 (Fis9431DRAFT_3240 and Fis9431DRAFT_1465), *Fischerella* sp. PCC 9339 (PCC9339DRAFT_06667 and PCC9339DRAFT_03590), *Fischerella* sp. JSC11 (FJSC11DRAFT_3026 and FJSC11DRAFT_1770), *Fischerella muscicola* SAG 1427-1 (UYGDRAFT_02065, UYGDRAF_05877 and UYGDRAF_03815), *Fischerella muscicola* PCC 7414 (UYIDRAFT_00914 and UYIDRAFT_02600), *Fischerella thermalis* PCC 7521 (UYKDRAFT_01108 and UYKDRAFT_04037), *Chlorogloeopsis fritschii* PCC 9212 (UYEDRAFT_05579 and UYEDRAFT_06592), *Chlorogloeopsis fritschii* PCC 6912 (UYCDRAFT_05865 and UYCDRAF_02201), *Mastigocladopsis repens* PCC 10914 (Mas10914DRAFT_3891) and *Mastigocoleus testarum* BC008 (YYIDRAFT_11082).

FIS9605DRAFT_04159 MHEELKKIVSQAMNTRFEFEQKLIQQAWENETFKQELLSNPRAVYARESKEELPKELEIEVIQESANKIYLVLPNNPA

Npun_R3212 -------MSEQQAQTRKDIESRIIAKAWKNEAFKQELLTNPKPIIEQEFGVELPAELNVSVYEENSTSLYFVLPILPQ

Npun_R3211 ------MSEQEQAQTRKNIEARIVAKAWKDEGYKQELLTNPKAIIEREFGVEFPAEVSVQVLEENSTSLYFVLPISPV

Npun_R3207 ------MSEQEQAQTRQDIEARIIAKAWKDESYKQELLTNSKAVIEREFGVEFPADVTVQVLQENPTSLYFVLPLSPT

Npun_R3209 ------MSEQEQAQTRQDIEARIIAKAWKDEAYKQELVTNPKAVIEREFGVEFPADVNVQVLEENPTSLHFVLPISPV

Npun_R3208 ------MTQQEQAQTRQDIEARIIAKAWKDEAYKQELLTNPKAVIEREFGVEFPADVNVQVLEENPTSLHFVLPISPV

Npun_R3210 ------MSEQEQAQTRQDIEARIIAKAWKDEVYKQELLTNPKAVIEREFGVEFPADVNVQVLEENPTSLHFVLPISPV

.: :** ::* ::: :**::* :****::* : : :* *:* :: :.* :*. ..:::*** *

FIS9605DRAFT_04159 PATTSGELSEADLETVAGGSCRYSSREYEES-C-------------SWFSASK----KRSAEGLV------------

Npun_R3212 --IEGRELSEEELESVAGGFIGGLITIAVGV--T---PFTGDIVKAT----KKLTKK--------------------

Npun_R3211 --AIAQELSEEQLEAIAGGYMTTLASANASAKINPILPIRHSLVKTLR-----------------------------

Npun_R3207 --AIMQELSEEQLQAIAGGGISAVIGNPKLISS----ALSGLLVSVSYLASHQIRR---------------------

Npun_R3209 --AIAQELSEEQLEAIAGGKDWRVELAKGALSV----GLAITLVDTL----KRLTK---------------------

Npun_R3208 --AIAQELSEEELLALAAGVNYSAVTVAIV----------KNTVKQN---TNIITRAAVSVTALVTGASIGASSVHL

Npun_R3210 --TIAQELSEEELLAIAAGGQIKELTKIS-----------ANLV--K---NYKTTRAAVSATALISGASIGASSVHL

**** :* ::*.*

**Sequence alignment of putative NHLP precursor peptides**. The conserved region near the peptide cleavage site is highlighted in the box. Putative NHLP precursor peptides from *Fischerella* sp. PCC 9605 (FIS9605DRAFT_04159) and *Nostoc punctiforme* PCC 73102 (Npun_R3212, Npun_3211, Npun_3207, Npun_3209, Npun_3208 and Npun_3210).
