# Supplementary material for: Genome mining for natural product biosynthetic gene clusters in the Subsection V cyanobacteria
Source: BMC Genomics. 2015 Sep 3;16(1):669. doi: 10.1186/s12864-015-1855-z (PMC4558948; doi:10.1186/s12864-015-1855-z)
Supplement: Additional file 7: — A domain binding pocket of NRPS-like enzyme identified in mys gene clusters from Subsection V cyanobacteria. (DOCX 18 kb) [file 12864_2015_1855_MOESM7_ESM.docx]

| **Additional file 7: A domain binding pocket of NRPS-like enzyme identified in** *mys* **gene clusters from Subsection V cyanobacteria.** | | | | | | | | | | | | |
| --- | --- | --- | --- | --- | --- | --- | --- | --- | --- | --- | --- | --- |
| Cyanobacteria | A domain binding pocket | | | | | | | | | | | Amino acid |
|  | 235 | 236 | 239 | 278 | 299 | 301 | 322 | 330 | 331 | 517 |  | |
| Ava_3855 | D | V | W | H | I | S | L | I | D | K | Serine | |
| PCC9339DRAFT_04154 | D | V | W | H | I | S | L | I | D | K | Serine | |
| UYCDRAFT_  06620 | D | V | W | H | I | S | L | I | D | K | Serine | |
| HT291_02286 | D | V | W | H | I | S | L | I | D | K | Serine | |
| IC523_01960 | D | V | W | H | I | S | L | I | D | K | Serine | |
| Fis9431DRAFT_  4778 | D | V | W | H | I | S | L | I | D | K | Serine | |
| UYGDRAFT_  04365 | D | V | W | H | I | S | L | I | D | K | Serine | |
| YYIDRAFT_  08384 | D | V | W | H | I | S | L | I | D | K | Serine | |
| UYEDRAFT_  04554 | D | V | W | H | I | S | L | I | D | K | Serine | |
