# Supplementary material for: Genome mining for natural product biosynthetic gene clusters in the Subsection V cyanobacteria
Source: BMC Genomics. 2015 Sep 3;16(1):669. doi: 10.1186/s12864-015-1855-z (PMC4558948; doi:10.1186/s12864-015-1855-z)
Supplement: Additional file 8: — Terpene gene clusters. (DOCX 20 kb) [file 12864_2015_1855_MOESM8_ESM.docx]

**Additional file 8: Terpene gene clusters**

Abbreviations: WI HT-29-1: *W. intricata* UH strain HT-29-1, HW IC-52-3: *H. welwitschii* UH strain IC-52-3, FS PCC 9431: *Fischerella* sp. PCC 9431, FS PCC 9399: *Fischerella* sp. PCC 9339, FM SAG 1427-1: *F. muscicola* SAG 1427-1, CF PCC 6912: *Chlorogloeopsis fritschii* PCC 6912, CS PCC 9212: *Chlorogloeopsis* sp. PCC 9212, MT BC008: *M. testarum* BC008. MR PCC 10914: *Mastigicladopsis repens* PCC 10914, FS PCC 9605: *Fischerella* sp. PCC 9605, FS JSC-11: *Fischerella* sp. JSC-11, FM PCC 7414: *Fischerella muscicola* PCC 7414, FT PCC 7521: *Fischerella thermalis* PCC 7521.

| Geosmin biosynthetic gene cluster: | | | | | |
| --- | --- | --- | --- | --- | --- |
|  | WI HT-29-1 | HW IC-52-3 | FS PCC 9431 | FM SAG 1427-1 | % identity |
| Geosmin synthase | HT291_02985 (748aa) | IC523_01603 (478aa) | Fis9431DRAFT_1946 (748aa) | UYGDRAFT_01254 (750aa) | 93.8 |
| Cyclic nucleotide-binding domain | HT291_02986 (469aa) | IC523_01602 (469aa) | Fis9431DRAFT_1947 (469aa) | UYGDRAFT_01255 (469aa) | 97.5 |
| Cyclic nucleotide-binding domain | HT291_02987 (460aa) | IC523_01601 (468aa) | Fis9431DRAFT_1948 (468aa) | UYGDRAFT_01256 (468aa) | 96.0 |

| Sesquiterpene synthase gene clusters: | | | | | |
| --- | --- | --- | --- | --- | --- |
|  | FS JSC-11 | FT PCC 7521 | FM PCC 7414 | FS PCC 9605 | % identity |
| Sesquiterpene synthase  (Terpene synthase metal-binding domain-containing protein) | FJSC11DRAFT_4294 (328aa) | UYKDRAFT_00942 (328aa) | UYIDRAFT_05501 (343aa) | FIS9605DRAFT_06376 (322aa) | 72.8 |
| Cytochrome p450 (COG2124) (Unspecific monooxygenase) | FJSC11DRAFT_4293 (462aa) | UYKDRAFT_00941 (462aa) | UYIDRAFT_05502 (462aa) | FIS9605DRAFT_06377 (463aa) | 79.7 |
| Hybrid two component protein (COG3899)  (Multi-sensor signal transduction multi-kinase or Predicted ATPase) | FJSC11DRAFT_4292 (1889aa) | UYKDRAFT_00940 (1889aa) | UYIDRAFT_05503 (1889aa) | FIS9605DRAFT_06378 (1907aa) | 84.3 |

| Sesquiterpene synthase gene cluster with cytochrome p450 replaced with oxidoreductase: | | | | | | |
| --- | --- | --- | --- | --- | --- | --- |
|  | FS PCC 9339 | WI HT-29-1 | HW IC-52-3 | FS PCC 9431 | FM SAG 1427-1 | % identity |
| Sesquiterpene synthase  (Terpene synthase metal-binding domain-containing protein) | PCC9339DRAFT_04774 (322aa) | HT291_03239 (322aa) | IC523_02161 (322aa) | Fis9431DRAFT_0377 (322aa) | UYGDRAFT_00443 (322aa) | 77.4 |
| Hypothetical protein (COG0654)  ( 2-polyprenyl-6-methoxyphenol hydroxylase and related FAD-dependent oxidoreductases) | PCC9339DRAFT_04773 (468aa) | HT291_03238 (468aa) | IC523_02160 (468aa) | Fis9431DRAFT_0376 (468aa) | - | 93.8 |
| Hybrid two component protein (COG3899) (Multi-sensor signal transduction multi-kinase or Predicted ATPase) | PCC9339DRAFT_04772 (1893aa) | HT291_-3237 (1896aa) | IC523_02159 (1896aa) | Fis9431DRAFT_0375 (1896aa) | UYGDRAFT_00444 (1893aa) | 87.4 |

| Squalene synthetase and phytoene desaturase gene cluster: | | |
| --- | --- | --- |
|  | Phytoene/  Squalene synthetase (COG1562) | Phytoene desaturase (COG3349) |
| FT PCC 7521 | UYKDRAFT_01525 (310aa) | UYKDRAFT _01526 (479aa) |
| FS JCS-11 | FJSC11DRAFT_3385 (310aa) | FJSC11DRAFT _3386 (479aa) |
| FM PCC 7414 | UYIDRAFT_00336 (310aa) | UYIDRAFT _00337 (479aa) |
| FS PCC 9339 | PCC9339DRAFT_02889 (310aa) | PCC9339DRAFT _02890 (749aa) |
| FM SAG 1427-1 | UYGDRAFT_03067 (310aa) | UYGDRAFT _03066 (479aa) |
| FS PCC 9431 | Fis9431DRAFT_1150 (310aa) | Fis9431DRAFT _1151 (479aa) |
| HW IC-52-3 | IC523_01577 (310aa) | IC523 _01576 (479aa) |
| WI HT-29-1 | HT291_04864 (310aa) | HT291 _04865 (479aa) |
| FS PCC 9605 | Fis9605DRAFT_02889 (310aa) | Fis9605DRAFT _02888 (479aa) |
| CF PCC 9212 | UYEDRAFT_03716 (310aa) | UYEDRAFT _03717 (488aa) |
| CF PCC 6912 | UYCDRAFT_03548 (310aa) | UYCDRAFT _03547 (488aa) |
| MR PCC 10914 | Mas10914DRAFT_0439 (310aa) | Mas10914DRAFT _0440 (493aa) |
| MT BC008 | YYIDRAFT_07817 (300aa) | YYIDRAFT_07818 (480aa) |
| % identity | 89.7 | 89.9 |

| Squalene synthetase and squalene-hopene cyclase gene cluster: | | | |
| --- | --- | --- | --- |
|  | Phtoene/squalene synthetase (COG1562) | Squalene-hopene cyclase (COG1657) | Hopene-associated glycoslytransferase HpnB (COG1215) |
| FT PCC 7521 | UYKDRAFT_03159 (271aa) | UYKDRAFT_03160 (636aa) | - |
| FS JCS-11 | FJSC11DRAFT_0758 (271aa) | FJSC11DRAFT_0759 (636aa) | - |
| FM PCC 7414 | UYIDRAFT_03635 (283aa) | UYIDRAFT_03634 (636aa) | - |
| FS PCC 9339 | PCC9339DRAFT_00383 (283aa) | PCC9339DARFT_00384 (636aa) | - |
| FM SAG 1427-1 | UYGDRAFT_05343 (283aa) | UYGDRAFT_05344 (636aa) | - |
| FS PCC 9431 | Fis9431DRAFT_2903 (283aa) | FisDRAFT_2904 (636aa) | - |
| HW IC-52-3 | IC523_03209 (283aa) | IC523_03208 (636aa) | - |
| WI HT-29-1 | HT291_00955 (283aa) | HT291_00956 (636aa) | - |
| FS PCC 9605 | FIS9605DRAFT_04621 (273aa) | FIS9605DRAFT_04622 (636aa) | - |
| CF PCC 9212 | UYEDRAFT_06118 (272aa) | UYEDRAFT_06117 (636aa) | UYEDRAFT_06116 (394aa) |
| CF PCC 6912 | UYCDRAFT_05467 (272aa) | UYCDRAFT_05468 (636aa) | UYCDRAFT_05469 (394aa) |
| MR PCC 10914 | - | Mas10914DRAFT_2871 (661aa) | Mas10914DRAFT_2870 (423aa) |
| % identity | 89.6 | 88.3 | 76.8 |

| MT BC008 terpene cluster: | | | | | |
| --- | --- | --- | --- | --- | --- |
|  | Hopene-associated glycosyltransferase HnpB (COG1215) | Phytoene/  Squalene synthetase (COG1562) | Phytoene/  Squalene synthetase (COG1562) | Phytoene desaturase (COG3349) | Squalene-hopene cyclase (COG1657) |
| MT BC008 | YYIDRAFT_05404 (397aa) | YYIDRAFT_05405 (310aa) | YYIDRAFT_05406 (312aa) | YYIDRAFT_05407 (420aa) | YYIDRAFT_05408 (655aa) |

| Additional single squalene synthetase genes: | |
| --- | --- |
| MR PCC 10914 | Mas10914DRAFT_2774 (273aa) |
| MT BC008 | YYIDRAFT_06629 (281aa) |
